# Systematic re-structure and new species of Sphaerodoridae (Annelida) after morphological revision and molecular phylogenetic analyses of the North East Atlantic fauna

**DOI:** 10.3897/zookeys.845.32428

**Published:** 2019-05-15

**Authors:** María Capa, Arne Nygren, Julio Parapar, Torkild Bakken, Karin Meißner, Juan Moreira

**Affiliations:** 1 University of the Balearic Islands, Department of Biology, Palma, Spain University of the Balearic Islands Palma Spain; 2 Norwegian University of Science and Technology, NTNU University Museum, Trondheim, Norway Norwegian University of Science and Technology Trondheim Norway; 3 Sjöfartsmuseet Akvariet, Göteborg, Sweden Sjöfartsmuseet Akvariet Göteborg Sweden; 4 University of A Coruña, Department of Biology, A Coruña, Spain University of A Coruña A Coruña Spain; 5 Forschungsinstitut Senckenberg, Deutsches Zentrum für Marine Biodiversitätsforschung (DZMB), Hamburg, Germany Forschungsinstitut Senckenberg, Deutsches Zentrum für Marine Biodiversitätsforschung Hamburg Germany; 6 Autonomous University of Madrid, Departament of Biology (Zoology), Madrid, Spain Autonomous University of Madrid Madrid Spain

**Keywords:** 16S rRNA, 18S rRNA, 28S rRNA, classification, COI, identification key, integrative taxonomy, morphology, new genus, new species, phylogeny, systematics

## Abstract

Detailed morphological study of more than 2600 North East Atlantic (NEA) sphaerodorids (SphaerodoridaeAnnelida) and phylogenetic analyses of DNA sequences of representatives of several identified morphospecies enforced changing the current systematic classification within the family allowed the discovery of new species provided new information about the morphological and genetic characterisation of members of this group and increased the species occurrence data to better infer their geographic and bathymetric distribution ranges. Phylogenetic analyses of nuclear (18S rRNA and 28S rRNA) and mitochondrial sequences (COI and 16S rRNA) of NEA short-bodied sphaerodorids revealed outstanding results including paraphyly of the genera *SphaerodoropsisSphaerodoridium*, and *Sphaerephesia*. The number of longitudinal and transverse rows of dorsal macrotubercles is proposed as potential synapomorphies for the main clades and are consequently herein used for the genera delimitation. The new classification proposed here implies nomenclatural changes and the erection of a new genus *Geminofilum***gen. n.** to accommodate the species previously considered as *Sphaerodoropsis* with two transverse rows of dorsal macrotubercles per segment. Four species are being described herein: *Euritmianordica* Capa & Bakken **sp. n.***Sphaerephesiamultichaeta* Capa Moreira & Parapar **sp. n.***Sphaerephesiaponsi* Capa Parapar & Moreira **sp. n.** and *Sphaerodoridiumceliae* Moreira Capa & Parapar **sp. n.** Characterisation of the other 21 species including updated iconography and an identification key to all NEA short-bodied sphaerodorids are provided.

## Introduction

Sphaerodoridae Malmgren, 1867 is a relatively small group (approximately 110–120 nominal species) of benthic marine worms, reported worldwide from intertidal to abyssal depths (Capa et al. 2014, 2016a). The monophyly of the group has been assessed recently and is evidenced by their well-defined external morphology (Capa et al. 2016a). Sphaerodorids are characterised by the presence of conspicuous epithelial tubercles arranged in more or less clear rows (longitudinal and transverse) and a thick cuticle without collagen (e.g., Ruderman 1911, Reimers 1933, Hausen 2005, Filippova et al. 2010, Capa et al. 2014, 2016a). Within Sphaerodoridae, two distinct groups with substantial morphological differences have been distinguished: the long-bodied forms (with elongate and slender bodies, clear segmentation, two longitudinal rows of macrotubercles with terminal papillae and presence of reduced dorsal cirri or microtubercles) and the short-bodied forms (usually measuring less than 5 mm long, with poorly delineated segments and a great variety of number and arrangement of epithelial dorsal tubercles) (Fauchald 1974, Helm and Capa 2015, Capa et al. 2016a).

Long-bodied sphaerodorids included members of *Ephesiella* Chamberlin, 1919, *Ephesiopsis* Hartman & Fauchald, 1971 and *Sphaerodorum* Ørsted, 1843, but it has recently been reviewed and all species transferred into the *Sphaerodorum* (Capa et al. 2018). Monophyly of some short-bodied genera (including *Clavodorum* Hartman & Fauchald, 1971, *Commensodorum* Fauchald, 1974, *Euritmia* Sardá-Borroy, 1987, *Sphaerephesia* Fauchald, 1972, *Sphaerodoridium* Lützen, 1961, and *Sphaerodoropsis* Hartman & Fauchald, 1971) still need assessment (Capa and Bakken 2015, Capa et al. 2016a).

The North East Atlantic (NEA), which includes the European part of the Atlantic, is dominated by deep ocean basins, including the Greenland, Lofoten, and Norwegian Basins, with depths down to 5000 m, and a shallow continental shelf along the European coast (Celtic Sea, Bay of Biscay and Iberian coast). This marine region holds a large diversity of Sphaerodoridae (Annelida) compared with other world geographic areas, with 26 species described or reported herein to date (Table 1). This diversity may reflect the collecting effort put into this biogeographic region during the last two centuries and the taxonomic expertise gathered in European countries. Nevertheless, this species list needs revision.

**Table 1.** Species of Sphaerodoridae (Annelida) (with nomenclature as in Read and Fauchald 2018) reported from the North Eastern Atlantic, with type locality and depth.

Species	Type locality	Depth
*Clavodorumfauchaldi* Desbruyères, 1980	Banc Le Danois, Bay of Biscay	1913 m
*Commensodorumcommensalis* (Lützen, 1961)	Kristineberg, Gullmarfjord, Sweden	35 m
*Ephesiellaabyssorum* (Hansen, 1882)	Off Møre og Romsdal, Norway	960 m
*Ephesiellaramosae* Desbruyères, 1980	Meriadzek Terrace, Bay of Biscay	2156 m
*Euritmiahamulisetosa* Sardá-Borroy, 1986	Tarifa, Gibraltar Strait	0.5 m
*Sphaerodoridiumclaparedii* Greeff, 1866	Dieppe, English Channel	(?)
*Sphaerodoridiumfauchaldi* Hartmann-Schröder, 1993	North Sea	172 m
*Sphaerodoridiumguerritai* Moreira & Parapar, 2015	Iceland	600 m
*Sphaerodoropsisamoureuxi* Aguirrezabalaga & Ceberio, 2005	Capbreton Canyon, Bay of Biscay	984–1029 m
*Sphaerodoropsisartabrensis* Moreira & Parapar, 2007	Artabro Gulf, NW Iberian Peninsula	209 m
*Sphaerodoropsisbaltica* Reimers, 1933	Kiel, Baltic Sea	6–8 m
*Sphaerodoropsischardyi* Desbruyères, 1980	Bay of Biscay	2430 m
*Sphaerodoropsisdistichum* (Eliason, 1962)	Skagerrak	460 m
*Sphaerodoropsisgarciaalvarezi* Moreira et al., 2004	Baiona, NW Iberian Peninsula	7 m
*Sphaerodoropsisgudmunduri* Moreira & Parapar, 2012	North Iceland	97 m
*Sphaerodoropsishalldori* Moreira & Parapar, 2012	Western Iceland	1162 m
*Sphaerodoropsislaureci* Desbruyères, 1980	Meriadzek Terrace, Bay of Biscay	2325 m
*Sphaerodoropsislongipapillata* Desbruyères, 1980	Bay of Biscay	4150 m
*Sphaerodoropsismartinae* Desbruyères, 1980	Banc Le Danois, Bay of Biscay	1913 m
Sphaerodoridiumcf.minutum (Webster & Benedict, 1887)	Off New England, USA,	continental shelf
*Sphaerodoropsisphilippi* (Fauvel, 1911)	Kara Sea	0–220 m
*Sphaerodoropsissibuetae* Desbruyères, 1980	Banc Le Danois, Bay of Biscay	1913 m
*Sphaerodoropsisstellifer* Aguirrezabalaga & Ceberio, 2005	Capbreton Canyon, Bay of Biscay	990
Sphaerodoropsiscf.parva (Ehlers, 1913)	Eastern Antarctica	380–3423 m
*Sphaerodorumflavum* Ørsted, 1843	Denmark	intertidal (?)
*Sphaerodorumophiuretos* Martín & Alvà, 1988	Pas-de-Calais, English Channel	intertidal

Some of the species described and reported from the NEA have a wide distribution range. For instance, *Sphaerodoridiumminutum* (Webster & Benedict, 1887) has been reported in both eastern and western coasts of the North Atlantic and in NEA, from the Arctic to temperate waters and from coastal and shelf habitats (Fauchald 1974). Contrarily, there are other species that seem to be uncommon, and a few that only have been reported once or twice, such as *Clavodorumfauchaldi* Desbruyères, 1980, *Euritmiahamulisetosa* Sardá-Borroy, 1987, *Sphaerodoropsisdistichum* (Eliason, 1962), *Sphaerodoropsislaureci* Desbruyères, 1980 or *Sphaerodoropsisstellifer* Aguirrezabalaga & Cebeiro, 2005. Some descriptions of the early-discovered species also need to be updated with additional morphological features and comments on intraspecific variation.

The aim of the present paper is to provide an accurate list of species of the so-called short- bodied sphaerodorids inhabiting the NEA sea floor, with updated descriptions, illustrations and a key for identification of morphospecies. DNA sequence data have been used to assess the evolutionary relationships between members of this family, evaluate the traditional classification, and better understand the boundaries between species and the genetic diversity within some of them.

## Materials and methods

Access to the following museum collections have allowed the revision of the type material of all available species and examination of additional non-type material (a total of over 2600 specimens): NTNU University Museum, Norwegian University of Science and Technology, Trondheim (**NTNU-VM**); Natural History Collections, University of Bergen (**ZMBN**); Museo de Historia Natural, Universidade de Santiago de Compostela (**MHN-USC**); Museo Nacional de Ciencias Naturales (**MNCN**); Museum national d’Histoire naturelle, Paris (**MNHN**); Zoological Museum Hamburg (**ZMH**); and Deutsches Zentrum für Marine Biodiversitätsforschung (**DZMB**), Hamburg; Senckenberg Museum Frankfurt (**SMF**); National Museum of Ireland (**NMI**); Icelandic Institute of Natural History, Reykjavik (**IINH**); Natural History Museum of Denmark, University of Copenhage (**NHMD**, previously ZMUC).

Some of the contemporary expeditions that have contributed with material to this project are: BIOICE project (1991–2004) and the IceAGE project (ongoing since 2011) around Iceland (Omarsdottir et al. 2013), the MAREANO Programme (2005-present) in Norwegian waters (Buhl-Mortensen et al. 2015), and the “Brattegard-Sneli” sampling programme (1980–87) (Oug et al. 2017).

### Morphological studies

Material examined was fixed in formalin and preserved in 70–80% ethanol or was directly preserved in 70–100% ethanol. Specimens were studied under dissecting and compound microscopes. Some dissected parapodia were mounted on a microscopic slide with glycerine. Drawings were made with an Olympus BX51 compound microscope with a drawing tube.

Micrographs were taken with a Dino-Lite digital microscope (AnMo Electronics Corporation, Taiwan) attached to the microscopes or with a LEICA DFC 420 camera attached to a Leica MZ 16A stereo microscope and a Leica DM 6000B compound microscopes (Leica Microsystems, Wetzlar, Germany). Stacks of multi-focus shots were merged into a single photograph to improve resolution with Leica APPLICATION SUITE v3.7 software (Leica Microsystems, Wetzlar, Germany).

Scanning electron micrographs were taken on specimens after dehydrating them in a series of 80, 90 and 100% ethanol before critical point or in a series of mixtures of absolute ethanol and Hexamethyldisilazane (HMDS) with the following ratios 2:1, 1:1, 1:2, and then into pure HMDS. The prepared samples were mounted on holders, sputter-coated with gold (10 nm thickness). The micromorphology and topography were determined using a Philips FEI INSPECT (Hillsboro, Oregon, USA) Scanning Electron Microscope (SEM) of the Museo Nacional Ciencias Naturales (Madrid, Spain), at the Cellular and Molecular Imaging Core Facility at NTNU. The samples were observed with the Back Scattering Electron Detector (BSED) with a resolution at high vacuum of 4.0 nm at 30 kV. Additional micrographs were taken in the Servicios de Apoio á Investigación-SAI (Universidade da Coruña-UDC, Spain); specimens were dehydrated in a graded ethanol series, prepared by critical-point drying using CO2, coated with gold in a BAL-TEC SCD 004 evaporator and examined and photographed under a JEOL JSM-6400.

Types of NEA species and others for comparison have been revisited when possible. This, together with the examination of additional material, provided additional information about the species distribution range.

Abbreviations used in figures:

**al** acicular lobe

**ap** antenniform papilla

**bp** basal papillae

**CH** chaetiger

**dhp** dorsal head papilla

**dp** dorsal papilla

**go** genital opening

**gp** genital pores

**gs** genital structure

**la** lateral antenna

**ma** median antenna

**mo** mouth

**mt** macrotubercle

**no** nuchal organ pits

**pa** palp

**pp** parapodial papilla

**s** spur

**st** stalked papilla

**tc** tentacular cirrus

**vc** ventral cirrus

**1^st^** parapodia from first chaetiger

### DNA sequence analyses

A selection of specimens (86) of a variety of morphospecies collected in different localities in the North East Atlantic and some other Atlantic localities, and fixed in 100% ethanol were included in the analyses. DNA was extracted with QuickExtract DNA Extraction (Epicentre); a small piece, usually one or two parapodia, were put in 50–100 µl QuickExtract, and treated with 65 °C for 45 min followed by 2 min in 95 °C in a dry block thermostat. We used the primers 16SANNF (GCGGTATCCTGACCGTRCWAAGGTA) (Sjölin et al. 2005) or 16SARL (CGCCTGTTTATCAAAAACAT), together with 16SBRH (CCGGTCTGAACTCAGATCACGT) (Palumbi 1996) for 16S rDNA; LCO1490 (GGTCAACAAATCATAAAGATATTGG) and HCO2198 (TAAACTTCAGGGTGACCAAAAAATCA) (Folmer et al. 1994) for COI; 28SC1 (ACCCGCTGAATTTAAGCAT) and 28SD2 (TCCGTGTTTCAAGACGG) (Lê et al. 1993) for 28S rDNA (D1-D2 region); and 18SAL (AACCTGGTTGATCCTGCCAGT and CCAACTACGAGCTTTTTAACTG), 18SBO (TGATCCTTCCGCAGGTTCACCT and AAGGGCACCACCAGGAGTGGAG), and 18SCY (CGGTAATTCCAGCTCCAATAG and CAGACAAATCGCTCCACCAAC) (Apakupakul et al. 1999), amplifying three overlapping fragments, for 18S rDNA. PCR mixtures contained 0.33 µl of each primer (10µM), 1 µl of DNA template, and 10 µl of RedTaq 1.1x MasterMix 2.0 mM MgCl2 (VWR). Temperature profile was as follows: 96 °C/1min – (95 °C/30s – 52 °C (for COI, 16S rDNA, and 18S rDNA) or 60 °C (for 28S rDNA)/30s – 72 °C/60s) x 29 cycles – 72 °C/7min. PCR products were visualized with UV-light (312 nm) following electrophoresis for c. 15 minutes on a 1% agarose gel (1 g Agarose DNA Pure Grade (VWR) in a TAE Buffer Ultra Pure Grade (Amresco)) containing 1 µl GelRed Nuclear Acid Stain (Bioticum) in 50 ml agarose. Each PCR product was purified with 2 µl cleaning solution made from 500 µl mQ-H20, 40 µl FastAP (EF0651), 45 µl Buffer FastAP, and 20 µl Exonuclease (EN0581) (ThermoScientific). PCR products with added cleaning solution were run for 37 °C in 60 minutes, followed by 75 °C in 15 minutes. Sequencing was performed at Eurofins Genomics, DNA Sequencing Department in Ebersberg, Germany.

Overlapping sequence fragments were merged into consensus sequences using Geneious version 7.0.6 available from http://www.geneious.com/. We used MAFFT v7.017 (Katoh et al. 2002) within Geneious 7.0.6 with the following settings: algorithm=E-INS-i, scoring matrix=200PAM / k=2, gap open penalty=1.53 to align the sequences. We used the online GBlocks server v. 0.91b (Castresana 2002), using the options ‘Allow gap positions within the final blocks’ and ‘Allow less strict flanking positions’, to detect alignment-ambiguous sites (Talavera and Castresana 2007). Analyses were performed both with and without these alignment-ambiguous sites. Gene partitions were concatenated using Mesquite v. 2.75 (Maddison and Maddison 2008).

The mitochondrial (COI and 16S rRNA) and nuclear data sets (18S rRNA and 28S rRNA) were analysed separately and combined using Bayesian inference (BA), and Maximum Likelihood (ML). Bayesian analyses (BAs) of separate and combined data sets were run in MrBayes 3.2 (Ronquist and Huelsenbeck 2003), and the best-fit models were selected using the Akaike information criterion in JModel (Darriba et al. 2012). The protein-coding gene COI was further divided into two partitions, one with the first and second positions, and one with the third positions. The selected best-fit models were a general time reversible model with gamma distributed rate across sites and a proportion of the sites invariable (GTR+I+G) for the COI-partition with first and second positions, 16S, 18S, and 28S, while a Hasegawa, Kishino and Yano model, with gamma distributed rate across sites (HKY+G) was selected for the COI-partition with third positions.

Partitions were unlinked for the parameters statefreq, revmat, shape, and pinvar. Rateprior for the partition rate multiplier was set to be variable. Number of generations was set to 3 million, with four parallel chains (three hot, one cold), sample frequency was set to 1000, and number of runs set to two. One fourth of the samples were discarded as burn-ins. Maximum likelihood analyses (ML) were performed in raxmlGUI (Silvestro and Michalak 2012). In RAxML, the analyses were run with the GTRGAMMAI model, the combined data set was partitioned as in BA, and clade support was assessed using 1000 bootstrap replicates.

## Results

### Phylogenetic analyses

The selected best-fit models were a general time reversible model with gamma distributed rate across sites and a proportion of the sites invariable (GTR+I+G) for the COI-partition with first and second positions, 16S, 18S, and 28S, while a Hasegawa, Kishino, and Yano model with gamma distributed rate across sites (HKY+G) was selected for the COI-partition with third positions.

The removal of poorly aligned positions from divergent regions of 16S, 18S and 28S from the alignments with GBLOCKS v. 0.91b did not affect the phylogenetic results, neither tree topology nor node supports (not shown); hence, all further analyses were conducted with the complete sequences for these markers.

### Atlantic sphaerodorid relationships and subsequent nomenclatural changes

**Figure 1.**
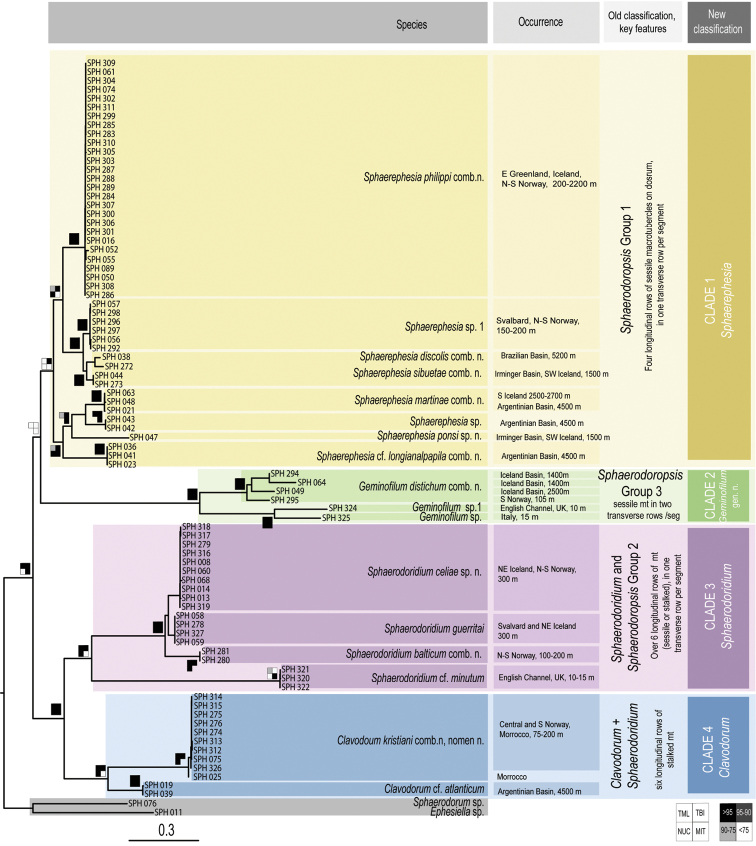
Consensus tree obtained after Bayesian analysis of the combined nuclear and mitochondrial dataset. Coloured squared on nodes (as indicated in the bottom of the figure) indicate (from top left to bottom right): TBI, Bayesian posterior probabilities of total dataset; TML, Maximum Likelihood Boostrap values of total dataset; NUC, Bayesian posterior probabilities of nuclear partition; MIT, Bayesian posterior probabilities of mitochondrial partition.

Analyses of combined mitochondrial (COI + 16S rDNA) and nuclear DNA sequence data (18S rDNA, and 28S rDNA) recover four main well-supported clades (Fig. 1, in different colours). These four groups are not congruent with the current sphaerodorid genera that mainly take into account the morphology of the dorsal tubercles (e.g., *Clavodorum* Hartman & Fauchald, 1971 and *Sphaerodoridium* Lützen, 1961 bearing stalked macrotubercles, and *Sphaerephesia* Fauchald, 1972 having macrotubercles with terminal papilla). Some of these traditional genera are therefore not recovered monophyletic herein. For instance, *Sphaerodoropsis* Hartman & Fauchald, 1971 is split into three, and *Sphaerodoridium* into two polyphyletic clades. These findings involve modifications in the current classification and also consequent nomenclatural changes. However, members of each of the four resulting clades in the molecular topology share the following morphological features related to the number of longitudinal and transverse rows of dorsal macrotubercles (Fig. 1):

**Clade 1** (Fig. 1, in yellow) comprises sphaerodorids with four longitudinal rows of sessile macrotubercles, arranged in a single transverse row per segment, regardless if they have a smooth surface or a terminal papilla, or if they are spherical or hemispherical in shape. This clade is therefore merging members of the traditionally considered *Sphaerodoropsis* Group 1 according to Borowski (1994) (with four longitudinal rows of smooth macrotubercles), and also members of *Sphaerephesia* (with macrotubercles provided with a terminal papilla). In all analyses, the specimens bearing hemispherical macrotubercles with terminal papilla (e.g., *Sphaerephesiasibuetae* (Desbruyères, 1980), *Sphaerephesiaponsi* sp. n.) are scattered within the clade together with specimens with hemispherical and smooth tubercles (e.g., *Sphaerodoropsismartinae* Desbruyères, 1980) or with spherical or pear-shaped macrotubercles (e.g., *Sphaerodoropsisphilippi* Fauvel, 1911). This provides evidence that the presence of a terminal papilla vs smooth and rounded/pear-shaped macrotubercles, has no phylogenetic implication.

The support values for Clade 1 are high after BI of the complete dataset, but moderate after ML analyses of the complete dataset or the partition of nuclear DNA. Analyses of only the mitochondrial sequence data (COI + 16S) did not recover Clade 1 as monophyletic and instead Clade 2 (Fig. 1, in green) was nested within, as sister to *S.philippi*.

The type species of both genera are not included in the present analyses, but it is expected that *Sphaerodoropsissphaerulifer* Moore, 1909, type species of *Sphaerodoropsis*, would not be recovered within this clade as it bears more than four longitudinal rows of macrotubercles over dorsum (*Sphaerodoropsis* Group 2 according to Borowski, 1994). *Sphaerephesialongisetis* Fauchald, 1972, type species of *Sphaerephesia*, bears macrotubercles arranged in four longitudinal rows. It is therefore proposed herein that the members of this clade and other sphaerodorids not include in the analyses but sharing this morphological feature, including *S.longisetis*, keep the generic name *Sphaerephesia*. It is thus herein proposed that all members of *Sphaerodoropsis* group 1 are synonymised. The main diagnostic feature for the emended *Sphaerephesia* is the presence of four longitudinal rows of macrotubercles in one transverse row per segment.

Seven subclades, congruent with the identified morphospecies, are found within Clade 1. Of these, *Sphaerodoropsisphilippi, Sphaerodoropsis* sp. 1, *S.sibuetae*, *S.martinae*, and *S.ponsi* sp. n. are present in the NE Atlantic, the other two species included in the analyses were collected from the Argentina Basin.

**Clade 2** (Fig. 1, in green) consists of specimens bearing two transverse rows of sessile macrotubercles, corresponding to the *Sphaerodoropsis* Group 3 (according to Borowski 1994). This clade is well supported after analyses of the combined as well as separate nuclear and mitochondrial datasets. However, its position in the topology varied depending of the analyses performed. Nuclear genes alone, recover this clade branching off at the base of the tree (low support) while the mitochondrial dataset recovers this clade as sister to *S.philippi* (high support). The type species of *Sphaerodoropsis, S.sphaerulifer*, bears dorsal macrotubercles in a single transverse row per segments, not in two. Therefore, a new genus needs to be erected to accommodate sphaerodorids with this feature: *Geminofilum* gen. n.

Six genetically distinct terminals showing long branches were recovered after analyses of molecular data besides the small morphological differences between them. Four of these specimens are herein identified as *Geminofilumdistichum* comb. n. (further analyses will need to determine species boundaries within this suspected species complex), and two as distinct unidentified species from the UK and Italy. All in all, this clade is morphologically homogenous, sharing the number and distribution of dorsal macrotubercles, chaetal morphology, and number and arrangement of parapodial papillae. Main differences rely on the number and distribution of epithelial papillae between dorsal transverse rows of dorsal macrotubercles. Differences in pigmentation in live specimens have been noticed (e.g., Fig. 8) and could provide valuable information for species distinction.

**Clade 3** (Fig. 1, in purple) gathers sphaerodorids with 7–12 longitudinal rows of small macrotubercles, arranged in a single transverse row per segment. In this clade, some of the previously considered species of *Sphaerodoropsis* group 2 (sensu Borowski 1994) (with sessile macrotubercles) are nested within members of *Sphaerodoridium* (with 7–12 longitudinal rows of stalked macrotubercles). This clade is well supported after analyses of the combined and nuclear datasets. However, Bayesian analysis of the mitochondrial dataset recovers Sphaerodoridiumcf.minutum branching off at the base of the ingroup (not in the ML analyses). On the basis of our findings we argue that the type species of *Sphaerodoridium*, *Sphaerodoridiumclaparedii* (Greeff, 1866), with eight longitudinal rows of macrotubercles would belong to this group. Since this name is older than the type species of *Sphaerodoropsis*, *S.sphaerulifer*, also with 7–8 longitudinal rows, we are consequently proposing to maintain the genus name *Sphaerodoridium* for members of this clade. Four species (morphospecies congruent with the lineages recovered), all from the NEA, form this clade, these are: *Sphaerodoridiumceliae* sp. n., *Sphaerodoridiumguerritai* Moreira & Parapar, 2015, *Sphaerodoridiumbalticum* (Reimers, 1933), comb. n., and Sphaerodoridiumcf.minutum (Webster & Benedict, 1887).

**Clade 4** (Fig. 1, in blue) consists of two well-defined lineages, corresponding to the diagnosis of the previously known species *Sphaerodoridiumfauchaldi* Hartmann-Schröder, 1993, now re-named as *Clavodorumkristiani* (Hartmann-Schröder, 1993), comb. n., nom. n. for the reasons given below, and Clavodorumcf.atlanticum Hartman & Fauchald, 1971. The concept of *Sphaerodoridium*, as traditionally understood (sphaerodorids with stalked macrotubercles and a short median antenna), has here been shown invalid as members within this diagnosis are split into two different clades (Clades 3 and 4, Fig. 1), containing each *Sphaerodoropsis* and *Clavodorum* species respectively. We therefore propose that *Sphaerodoridium* species with six longitudinal rows of stalked macrotubercles (and by definition, sphaerodorids with a long median antenna) are transferred to *Clavodorum* (sphaerodorids with six longitudinal rows of stalked macrotubercles and a short median antenna) and keep the latter name since *Sphaerodoridium* is occupied by members of Sphaerodoridae with more than six rows of longitudinal macrotubercles in one single row per segment (Clade 3 above).

### Taxonomic accounts

#### Clavodorum

Taxon classificationAnimaliaPhyllodocidaSphaerodoridae

Hartman & Fauchald, 1971

Sphaerodoridium Lützen, 1961: 415 (in part); Fauchald 1974: 270; Capa et al. 2014: 15.
Clavodorum Hartman & Fauchald, 1971: 63; Fauchald 1974: 262; Bakken 2002: 198; Capa et al. 2014: 15.

##### Type species.

*Clavodorumatlanticum* Hartman & Fauchald, 1971.

##### Diagnosis.

Body generally short and ellipsoid. Head appendages smooth without spurs or basal papillae. Median antenna shorter, equal, or longer than lateral antennae; antenniform papillae absent. Dorsal macrotubercles stalked, without terminal papilla, arranged in up to six longitudinal rows, one transverse row per segment; smaller tubercles, similarly stalked, may form irregular rows on ventrum. Microtubercles (small tubercles with collar and terminal papilla) absent. Stout hooks in the first chaetiger absent. Parapodia with large ventral cirri. All chaetae compound.

##### Remarks.

The relative length of the median antenna with respect to the lateral ones was the single reported morphological feature separating the traditional *Clavodorum* (with a median antenna longer than the lateral, or similar in length) and *Sphaerodoridium* (with a shorter median antenna), the two genera considered to bear stalked dorsal macrotubercles prior to the present study (e.g., Hartman and Fauchald 1971, Fauchald 1974). However, there has been some debate if this character alone was enough to split species in these two genera (Hartmann-Schröder and Rosenfeldt 1990, Borowski 1994, Capa et al. 2016a).

After analyses of molecular data performed in this study (Fig. 1) species with sessile macrotubercles (e.g., *Sphaerodoropsisbalticum*) were recovered nested within those with stalked macrotubercles (e.g., *Sphaerodoridiumminutum* and *Sphaerodoridiumguerritai*), requiring nomenclatural changes. Moreover, species with apparently shorter or longer median antenna are mixed in two different clades.

There seems to be some synapomorphies, related to the number of longitudinal rows of dorsal macrotubercles (Clade 3 and 4 in Fig. 1), characterizing these two clusters. In one clade, all the species bear up to six longitudinal rows of stalked macrotubercles (including the type species of *Clavodorum*, *Clavodorumatlanticum*), and in the other species bear more than six longitudinal rows of macrotubercles, regardless if they are sessile or stalked (including the type species of *Sphaerodoridium*, *Sphaerodoridiumclaparedii*). This pattern should be corroborated after including additional taxa in analyses, but it also requires some changes in the traditional classification.

Diagnostic features characterising *Clavodorum*, as traditionally understood, such as presence of postchaetal lobes (Fauchald 1974, Bakken 2002) or presence of nephridiopores in all chaetigers except for the first and the last three or four (Hartman and Fauchald 1971, Bakken 2002), have been omitted from the diagnosis as it has not been verified in some of the specimens examined nor are mentioned in the original description.

The species included in *Clavodorum* after this study are:

*Clavodorumadriaticum* Katzmann, 1973

Type locality: Zlarin, Adriatic Sea, 20–60 m.

*Clavodorumantarcticum* Hartmann-Schröder & Rosenfeldt, 1990

Type locality: Elephant Island, north of Antarctic Peninsula, 262 m.

*Clavodorumatlanticum* Hartman & Fauchald, 1971

Type locality: northwest of Bermuda in 4700–3800 m.

*Clavodorumclavatum* Fauchald, 1972

Type locality: Off El Segundo, California, 18–45 m.

*Clavodorumfauchaldi* Desbruyères, 1980

Type locality: Banc Le Danois, Bay of Biscay, 1913 m.

*Clavodorumfusum* (Hartman, 1967)

Type locality: Antarctic Peninsula, 128–165 m.

*Clavodorumkristiani* (Hartmann-Schröder, 1993), comb. n., nom. n.

Type locality: North Sea, off Scotland, 172 m.

*Clavodorumlongipes* Fauchald, 1974

Type locality: Off Mozambique, 5119 m.

*Clavodorumlutzeni* (Kudenov, 1987), comb. n.

Type locality: Off Florida, Gulf of Mexico, 37 m.

*Clavodorummexicanum* Kudenov, 1987

Type locality: Off Florida, Gulf of Mexico, 48 m.

#### Clavodorum fauchaldi

Taxon classificationAnimaliaPhyllodocidaSphaerodoridae

Desbruyères, 1980

**Figure 2.**
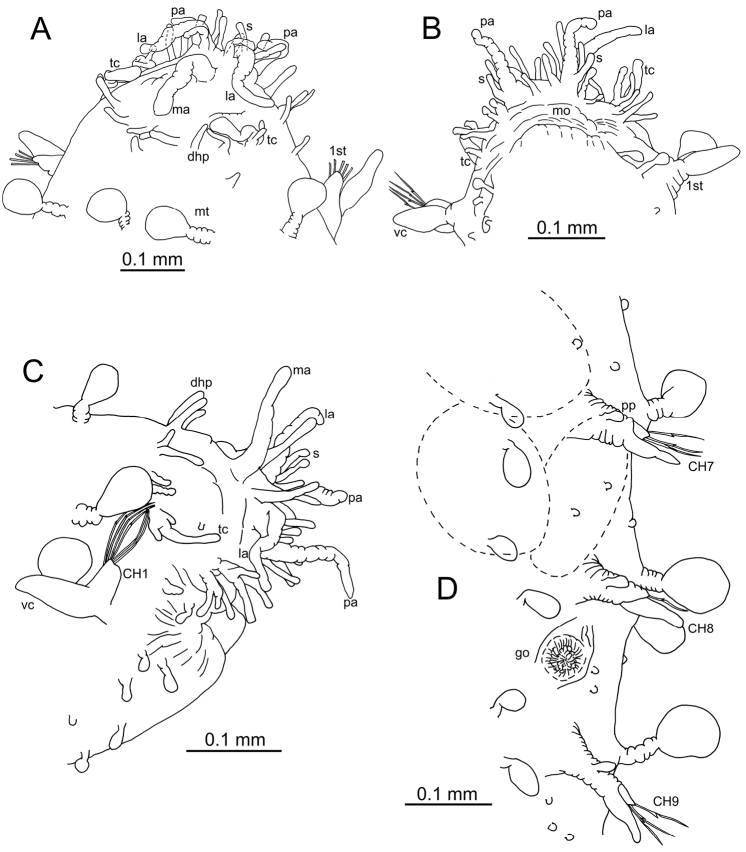
*Clavodorumfauchaldi* (IINH 38781: **A, D**; IINH 38786: **B, C**), line drawings. **A** Anterior end, dorsal view **B** anterior end, ventral view **C** anterior end, lateral view **D** female, chaetigers 7–9, ventral view, showing genital (?) opening.

**Figure 3.**
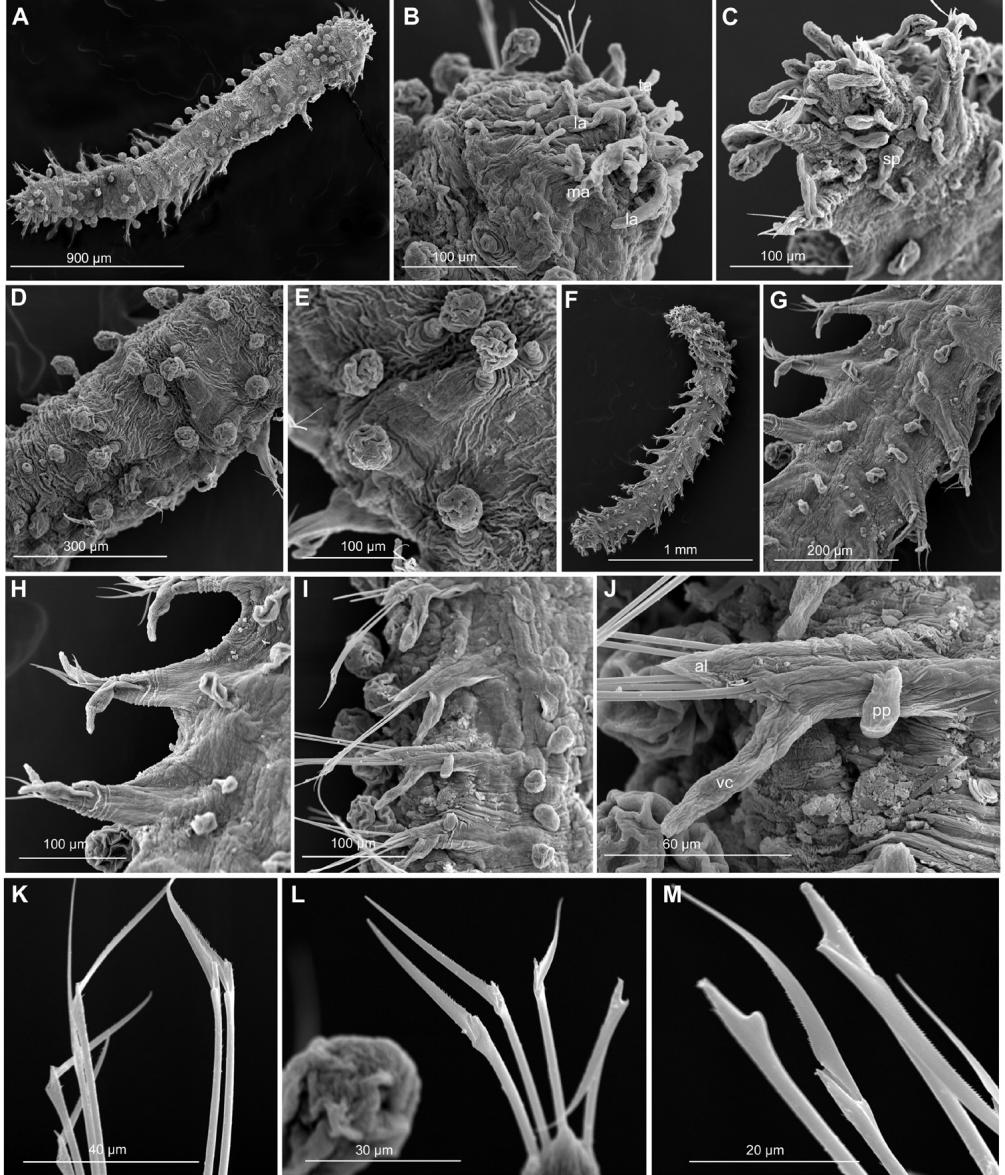
*Clavodorumfauchaldi* (IINH 38781: **A–E, K–M**; IINH 38782: **F–J**), scanning electron micrographs. **A** Complete specimen, lateral view **B** anterior end, dorsal view **C** anterior end, ventral view **D** mid-body chaetigers, arrangement of dorsal macrotubercles and papillae **E** dorsal macrotubercles and papillae, detail **F** complete specimen, ventral view **G** mid-body chaetigers, arrangement of ventral tubercles **H, I** mid-body chaetigers, parapodia and ventral tubercles **J** mid-body parapodium, anterior view **K–M** compound chaetae.

**Figure 4.**
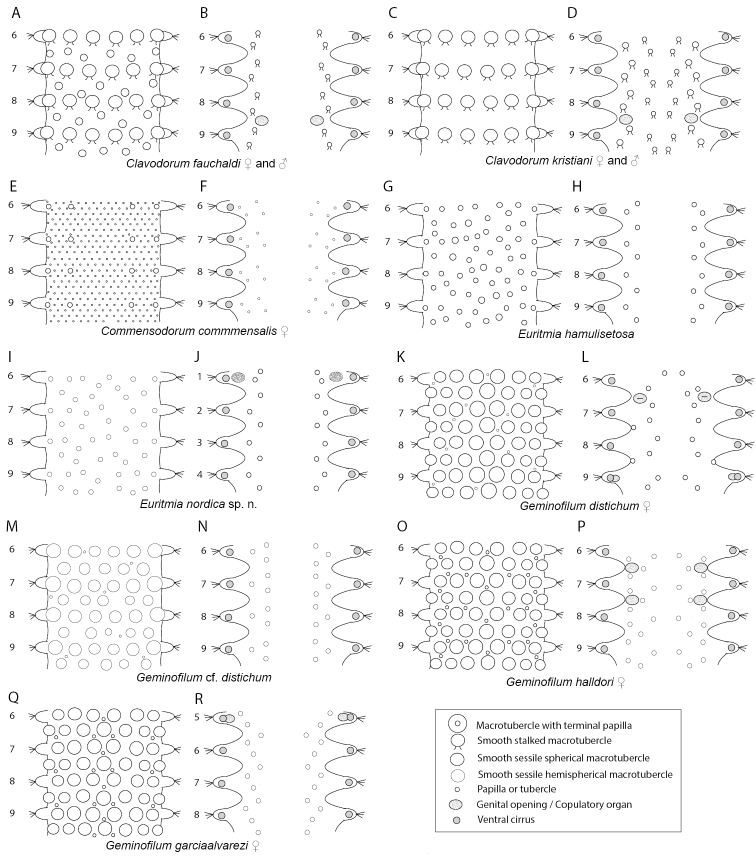
Stylized drawings of selected dorsal (left) and ventral (right) segments of species of *Clavodorum*, *Commensodorum*, *Euritmia* and *Geminofilum* gen. n., showing number and arrangement of epithelial tubercles and papillae.

**Figure 5.**
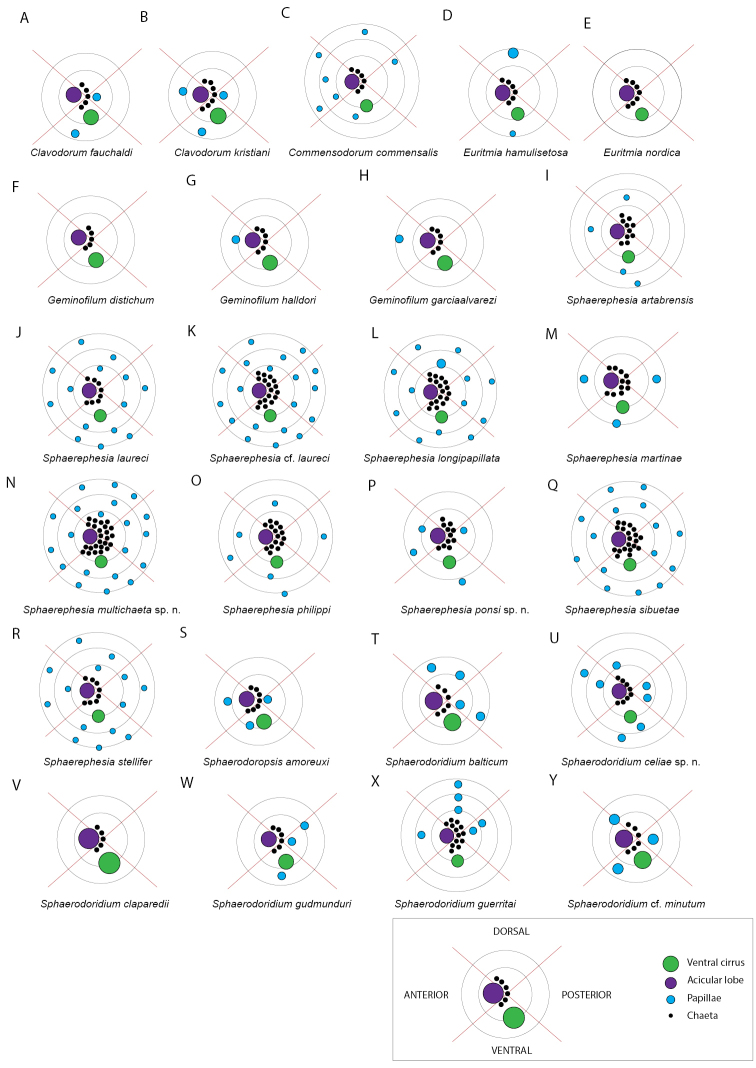
Stylized drawings of parapodia, showing the relative position and arrangement of parapodial lobes, cirri, and papillae, in mid-body chaetigers of all sphaerodorid species reported in the North East Atlantic waters.

Figs 2 , 3 , 4A, B , 5A

Clavodorum fauchaldi Desbruyères, 1980: 110–112, fig. 1.

##### Type locality.

Banc Le Danois, Bay of Biscay, 44°05.2'N, 4°19.4'W, 1913 m.

##### Material examined.

**Holotype**: MNHN POLY TYPE 1279, Bay of Biscay, Banc Le Danois, 44°05.2'N, 4°19.4'W, 1913 m, 1972.

##### Additional material.

(141 specs) **Iceland**: IINH 38781 (55 specs,5 on SEM stub), 62°20.50'N, 16°59.30'W, 2074 m, 29 Aug 1995; IINH 38782 (25 specs -6 on SEM stub-), 61°50.22'N, 16°52.86'W, 2270 m, 29 Aug 1995; IINH 38783 (5 specs), 65°15.61'N, 28°50.15'W, 1300 m, 25 Aug 1996; IINH 38784 (2 specs), 60°02.03'N, 22°27.17'W, 2537 m, 29 July 2000; IINH 38785 (3 specs), 62°24.80'N, 19°48.60'W, 1780 m, 13 Sep 2001; IINH 38786 (1 spec.), 62°48.00'N, 16°14.80'W, 1813 m, 16 Sep 2001; IINH 38787 (30 specs), 62°02.40'N, 19°38.71'W, 1678 m, 3 Sep 2002; IINH 38788 (18 specs), 62°31.14'N, 17°09.87'W, 1940 m, 7 Sep 2002; IINH 38789 (1 spec.), 62°47.19'N, 17°20.37'W, 1662 m, 8 Sep 2002; IINH 38790 (1 spec.), 63°30.22'N, 29°38.40'W, 2233 m, 5 Sep 2003; ZMBN 127252 (1 spec.), South Iceland, 61°38.2'N16°27.7'W, 2355 m, 5 Jun 1983.

##### Diagnosis.

Body ellipsoid. Median antenna similar in length to lateral antennae; lateral antennae with three or four basal papillae each; antenniform papillae absent; palps with 2–3 basal papillae each. Dorsal macrotubercles stalked, without terminal papilla, arranged in five longitudinal rows in first 2–3 and last chaetigers, and six longitudinal rows in middle chaetigers; stalk and tubercle of similar length. Additional hemispherical papillae (ca. 10–12) distributed over dorsum in two irregular transverse rows, following a zig-zag pattern. Two rows of stalked smaller tubercles along ventrum, with two tubercles near each parapodium. Parapodia with acicular lobe from chaetiger 1, and large ventral cirri, surpassing length of acicular lobe. One ventral papilla from chaetiger 2–4; and one terminal postchaetal papilla from chaetiger 7–10.

##### Re-description of holotype.

*Measurements and general morphology.* Holotype oval in shape, measuring 2.7 mm long, 0.4 mm wide for 24 chaetigers.

*Head.* Head fused to first chaetiger, with elongated and digitiform prostomial appendages, reaching the end of head (Figs 2A–C, 3A). Palps and lateral antennae similar in length, with 2–4 digitiform, shorter and slightly thinner basal papillae. Median antenna, similar in length to lateral, lacking spurs or basal papillae (Fig. 2B). Tentacular cirri ca. half of the length of prostomial appendages and thinned. Some elongated papillae distributed randomly on posterior part of head (Fig. 3B). Proboscis retracted in holotype and muscular pharynx through segments 3–6.

*Tubercles*. Body with stalked dorsal macrotubercles distributed in five longitudinal rows, on three anterior and two posterior segments and six rows in segments in between, although some detached in holotype; one transverse row per segment (Figs 3A, D, E, 4A). Stalk ca. the length of the macrotubercle, the latter oval, smooth, lacking terminal papilla (Figs 2A, D, 3D, E). Dorsal papillae in two irregular transverse rows, 4–6 papillae each per segment, rounded in shape (Fig. 4A). Ventral surface with two longitudinal rows of oval and stalked tubercles, arranged in two transverse rows per segment; anterior row with smaller tubercles than those of posterior row within each segment (Figs 2D, 3F–I, 4B).

*Parapodia*. Parapodia cylindrical, longer than wide, similar in length along body, with almost similar in length ventral cirri, digitiform or slightly tapering in width distally, well surpassing length of acicular lobe (Figs 2D, 3G–J). One large and rounded parapodial papilla from segment 2, located on the distal half of the antero-ventral surface of parapodia. One terminal papilla present from chaetiger 8, digitiform (Fig. 5A), and increasing its size to posterior chaetigers. One straight acicula supporting each parapodia, curved in first chaetigers.

*Chaetae*. All chaetae compound, ca. six in first segment to eight in middle chaetigers; with long, straight, unidentate and finally serrated blades. Blades similar in length between and within chaetigers, ca. ten times longer than maximum blade width (Fig. 3K–M).

*Pygidium*. Paired anal cirri similar to dorsal macrotubercles and ventral digitiform anal papilla similar in length to lateral cirri.

*Reproductive features*. Gametes or sexual structures not observed in holotype.

##### Variation.

Intraspecific variation was assessed by examining a number of samples collected during the BIOICE project, in Iceland. Largest specimen examined 3.75 mm long, 1 mm wide and 28 chaetigers. Most Icelandic specimens measuring ca. 2 mm in length, 0.65 mm in width with 18–24 chaetigers. Median antenna usually as long as lateral antennae, depending on the state of contraction of specimens. Tentacular cirri shorter than prostomial appendages and provided each with two short papillae near the base. One dorsal transverse row of eight longer papillae (clavate or digitiform) behind median antenna and running at level of tentacular cirri; several digitiform papillae surrounding the mouth at each side (usually including one bifid and sometimes one trifid). Muscular pharynx extending over three chaetigers (5–7). In some elongated specimens stalk seems slightly longer than macrotubercle. Postchaetal papilla present from chaetiger 7 to last chaetiger. Parapodial antero-ventral papilla present from chaetigers 2–4, becoming more lateral in chaetigers 8–10; rounded to elongated in shape. Acicula is straight and chaetae blades show some gradation in length, being ventral ones slightly shorter in middle and posterior chaetigers. Several females with oocytes and males observed; both sexes show genital openings near the base of parapodia between chaetigers 8 and 9 (Fig. 4B).

##### Remarks.

The original description indicates that at least two specimens were found (holotype and another used for SEM) but only the holotype has been deposited in a museum collection (MNHN). All the specimens examined from Iceland bear three basal papillae on lateral antennae, variation that has been added into the diagnoses, at least one or two emerge directly from the base of antennae and the rest probably from the surroundings of the base. This species was described as new due to the uniqueness of its parapodia, with postchaetal lobe absent on anterior segments. However, we consider here that the postchaetal lobe is a postchaetal papilla. Furthermore, both females and males from Iceland samples show a pair of ventral genital openings (Fig. 2D); these may be interpreted as sexual structures and therefore this is the first mention for the genus of such features.

##### Distribution.

Iceland (present study), Bay of Biscay (Desbruyères 1980).

##### Habitat.

Bathyal soft bottoms (1300–2537 m) (Desbruyères 1980; this study).

#### Clavodorum kristiani

Taxon classificationAnimaliaPhyllodocidaSphaerodoridae

(Hartmann-Schröder, 1993), comb. n. nom. n.

**Figure 6.**
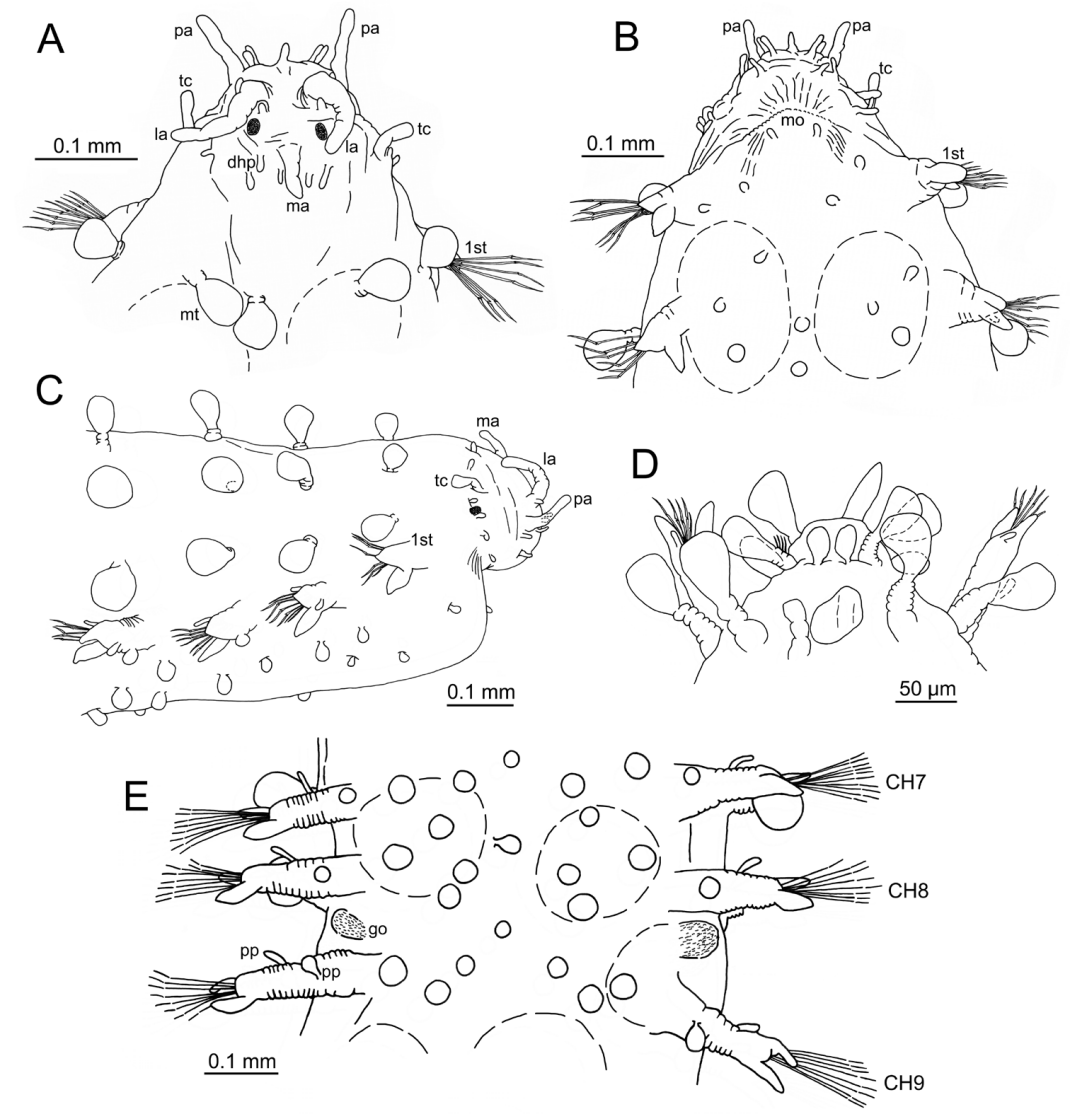
*Clavodorumkristiani* comb. n., nom. n. (MNCN 16.01/18458: **A, B**; MNCN 16.01/18459: **C, D**; MNCN 16.01/18457: **E**), line drawings. **A** Anterior end, dorsal view **B** same, ventral view **C** anterior end, lateral view **D** posterior end, ventral view **E** mid-body chaetigers, ventral view.

**Figure 7.**
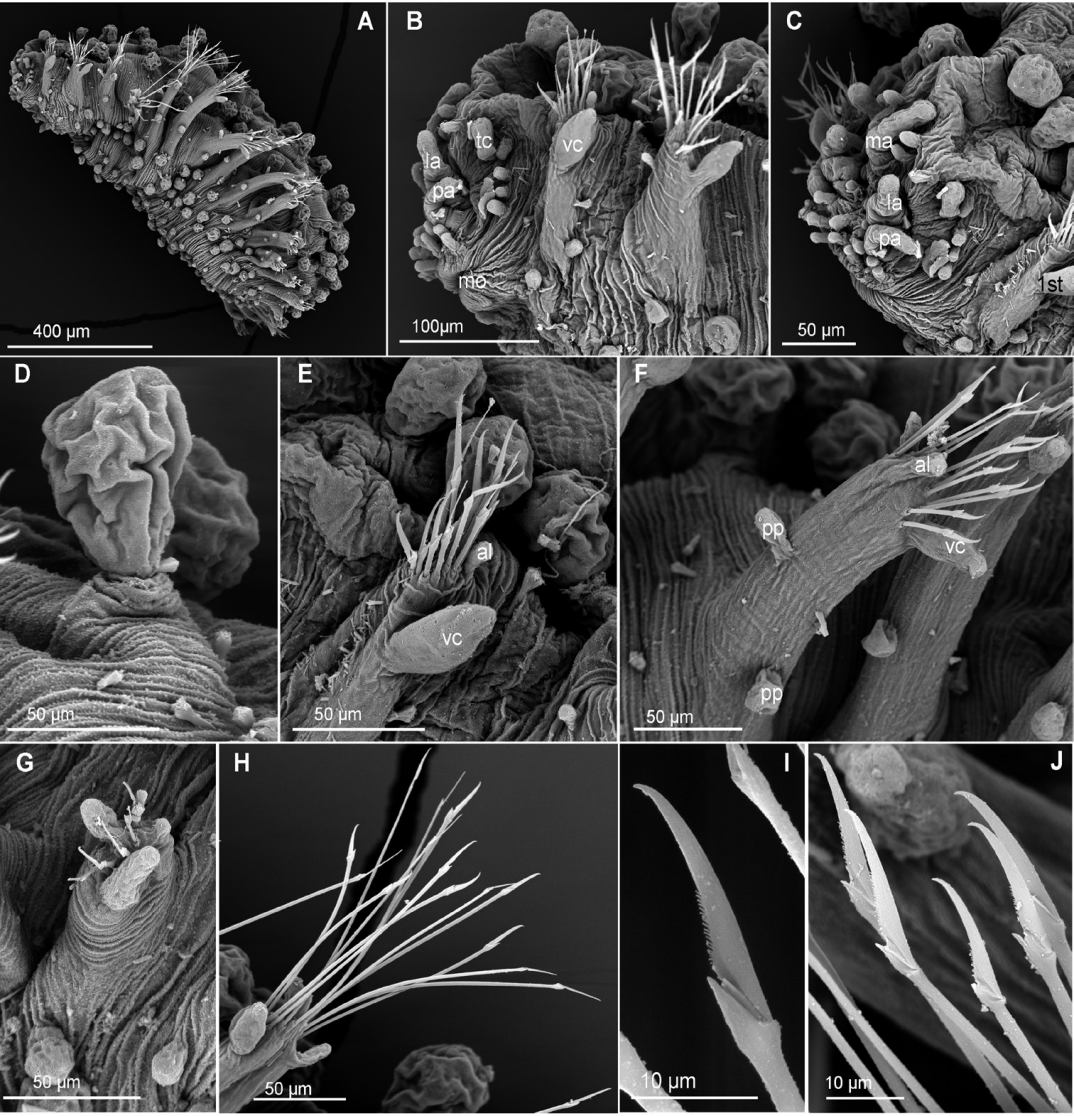
*Clavodorumkristiani* comb. n., nom. n. (Norway, NTNU-VM 74198). **A** Complete specimen, lateral view **B** anterior end, lateral view **C** same, slightly frontal **D** dorsal stalked macrotubercle, detail **E** left parapodium, chaetiger 1, latero-ventral view **F** same, chaetiger 7 **G** same, posterior chaetiger **H** chaetae fascicle, mid-body parapodium **I, J** chaetae, mid-body parapodium, detail.

**Figure 8.**
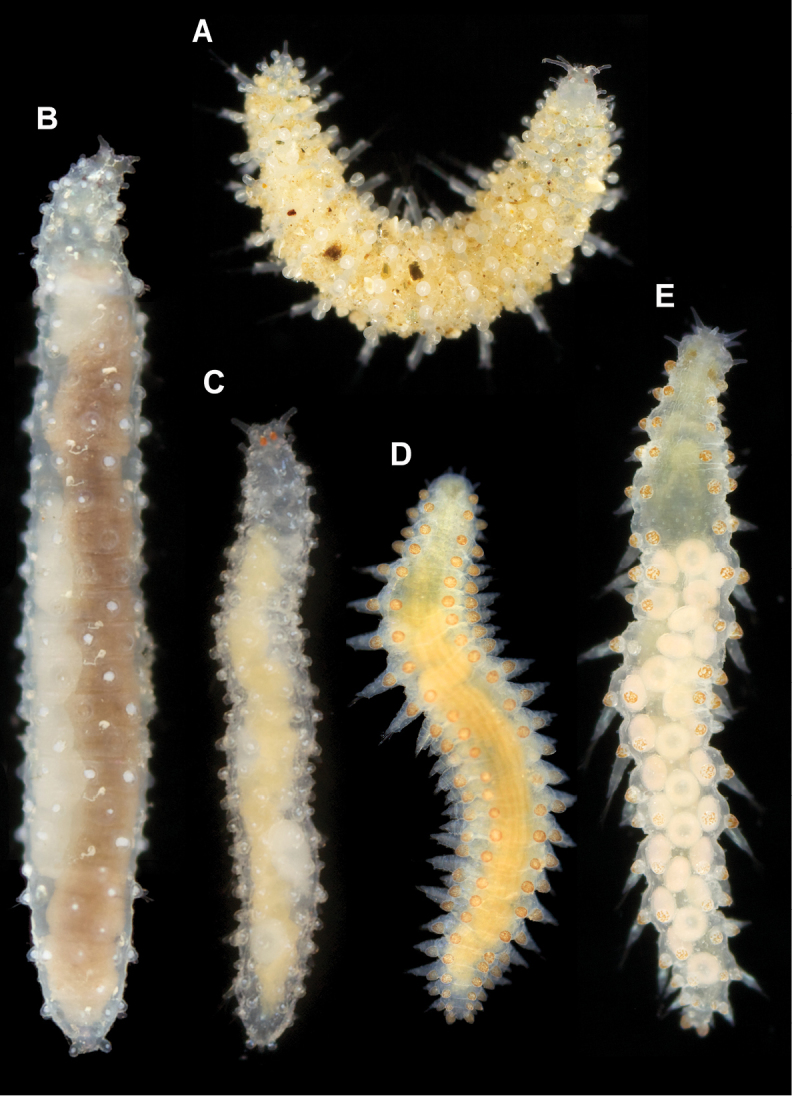
Photographs of live specimens (included in analyses shown in Fig. 1). **A***Clavodorumkristiani* comb. n., nom. n. (ZMBN 127258, SPH313) **B***Geminofilumdistichum* comb. n. from Skagerrak (ZMBN 127263, SPH295) **C***Geminofilum* sp. 1, from UK (SPH324) **D***Sphaerephesiaphilippi* comb. n., from Skagerrak (ZMBN 125432, SPH297) E *Sphaerephesiaphilippi* comb. n., from Finnmark (ZMBN 127311, SPH304).

Figs 4C, D , 5B , 6 , 7 , 8A

Sphaerodoridium fauchaldi Hartmann-Schröder, 1993: 123–125, figs 1–9; Hartmann-Schröder 1996: 234–235, fig. 104; Aguirrezabalaga and Cebeiro 2005: 16–19, figs 5, 6; Moreira and Parapar 2007: 377–378, fig. 3 B–E; Moreira et al. 2011: 26, fig. 3; Moreira 2012: 28–29, fig. 6.

##### Type locality.

North Sea, 58°16.98'N, 0°58.31'W, 172 m.

##### Material examined.

**Holotype**: ZMH P21082, North Sea, (N. Scotland) 58°16.98'N, 0°58.31'W, 172 m.

##### Additional material.

(140 specs) **Norwegian Sea**: NTNU-VM 74198 (3 specs in SEM stub), Sandsfjord, 62°12.3'N, 5°26.7'E, 85 m, 18 Oct 1987; ZMBN 127253 (1 spec.), Sandsfjord, 62°12.3'N, 5°26.7'E, 85 m, 18 Oct 1987; ZMBN 127256 (1 spec. for DNA sequencing, SPH274), Møre og Romsdal, 63°18.81'N, 6°39.24'E, 226 m, 26 Sep 2012; ZMBN 127257 (1 spec. for DNA sequencing, SPH275), Norwegian Sea, Møre og Romsdal, 63°18.81'N, 6°39.24'E, 226 m, 26 Sep 2012; NTNU VM 68172 (1 spec. used for DNA sequencing, SPH276), Ytre Mørebanken, 004°29.74'N, 62°36.86'E, 203 m, 03. Oct. 2012; ZMBN 103139 (1 spec. used for DNA sequencing, SPH075), 62°36.858'N, 4°29.742'E, Møre og Romsdal, 203 m, 3 Oct. 2012; NTNU VM 65127 (5 spec.), Halsafjord, Ytterfjorden, 63°10.17'N, 7°43.9'E, 150 m, 25 May 1884; ZMBN 127254 (1 spec. used for DNA sequencing, SPH314), Bergen, 60°16.181'N, 5°11.865'E, 120 m, 25 Jul 2014; ZMBN 127255 (1 spec. used for DNA sequencing, SPH315), Bergen, 60°16.181'N, 5°11.865'E, 120 m, 25 Jul 2014; ZMBN 125433 (1 spec. used for DNA sequencing, SPH312), Rogaland, Kvitsøy, 59°1.791'N, 5°26.929'E, 58 m, 10 Jun 2014; ZMBN 127258 (1 spec. used for DNA sequencing, SPH313 photographed alive, Fig. 8A), Rogaland, Karmøysundet, 59°17.273'N, 5°19.504'E, 74 m, 08 Jun 2014; ZMBN 127259 (1 spec. used for DNA sequencing, SPH326), Rogaland, Karmøysundet, 59°17.273'N, 5°19.504'E, 74 m, 08 Jun 2014. **Continental shelf, Galicia, NW Spain**: MNCN 16.01/18456 (27 specs), 43°35.45'N, 08°34.43'W, 152 m, 8 Sep 2002; MNCN 16.01/18457 (52 specs), 43°34.13'N, 8°36.56'W, 152 m, 14 Sep 2003; MNCN 16.01/18458 (42 specs), 42°30.39'N, 09°19.52'W, 147 m, 17 Sep 2004; MNCN 16.01/18459 (41 specs), 42°15.82'N, 09°22.68'W, 260 m, 23 Oct 2009. **Morocco**, ZMBN 103140 (1 spec. used for DNA sequencing, SPH025), Atlantic Ocean, 28°0.04836'N, 13°16.32'W, 100 m, 4 Dec 2011.

##### Diagnosis.

Body ellipsoid. Median antenna shorter than lateral antennae; lateral antennae and palps with 1–2 basal papillae each; antenniform papillae absent. Dorsal macrotubercles stalked, without terminal papilla, arranged in five longitudinal rows in first three chaetigers, and six longitudinal rows in following chaetigers; stalk and tubercle of similar length. Additional epithelial papillae on dorsum absent. Six to eight longitudinal rows of smaller tubercles with short stalk on ventrum; two tubercles near each parapodium slightly larger than others. Parapodia with long, oval acicular lobe from chaetiger 4; ventral cirri large, reaching or surpassing length of acicular lobe. Three parapodial papillae: one on antero-lateral surface from chaetiger 2, one ventral from chaetiger 3–4 and one terminal digitiform papilla from chaetiger 1, behind chaetae.

##### Re-description of holotype.

*Measurements and general morphology*. Body ellipsoid, measuring 1.0 mm long, 0.4 m wide, with nine segments; with rounded anterior and posterior ends, with slightly flat ventrum. Segmentation inconspicuous and pigmentation absent in preserved specimen (Fig. 8A).

*Head.* Head fused to first chaetiger, with elongated and digitiform prostomial appendages, reaching the end of peristomium (Figs 6A, 7C). Lateral antennae slightly longer than palps, with 1–2 digitiform, shorter and thinner basal papillae each. Median antenna shorter than lateral, lacking spurs or basal papillae. Five prostomial papillae between lateral antennae and palps (Figs 6A, B, 7C). Tentacular cirri approx. half of length of prostomial appendages and thinner, with a basal papilla each (Figs 6A, C, 7C). An irregular transverse row of ca. 6–10 elongated papillae behind median antenna and tentacular cirri. Up to eight elongated papillae surrounding mouth (Fig. 6B, C).

*Tubercles.* Body with stalked dorsal macrotubercles distributed in five longitudinal rows on three anterior segments and six rows in following segments; one transverse row per segment (Figs 4C, 6A, 8A). Stalk shorter or as long as macrotubercle, the later oval, smooth, and lacking terminal papilla (Fig. 7D). Ventral surface with 6–8 longitudinal rows of oval to rounded smaller tubercles with short stalk (Figs 4D, 6E); one (sometimes two) tubercles in between parapodia areas and six in parapodial areas; two tubercles closer to each parapodium slightly larger than others. Dorsum and lateral surfaces lacking papillae (Fig. 6C).

*Parapodia.* Parapodia cylindrical, longer than wide, increasing in length in mid-body. Acicular lobe long, oval from chaetiger 4 (Fig. 6C, E). Ventral cirri digitiform to conical, slightly tapering in width distally; as long or slightly shorter than parapodia in chaetigers 1–3; in following chaetigers at least reaching distal end of acicular lobe (Fig.&nbsp;7E, G). Parapodial papillae numbering usually up to three: one terminal digitiform papilla (“postchaetal lobe”) present from chaetiger 1 (sometimes two papillae in a few mid-body chaetigers); one large spherical to ellipsoid papilla from chaetiger 3–4, on proximal half of ventral surface of parapodia; one digitiform papilla from chaetiger 2, centred on anterior surface of parapodia (Figs 5B, 6E, 7E–G). One straight acicula supporting each parapodium.

*Chaetae.* All chaetae compound, ca. 7–8 in first segment to 12–13 in middle chaetigers; with long, straight, unidentate and finally serrated blades (Fig. 7H–J). Blades similar in length within chaetigers, slightly shorter in mid-body to posterior chaetigers; ca. 6–7 times longer than maximum width (Fig. 7I, J).

*Pygidium.* Paired anal cirri similar to dorsal macrotubercles and ventral digitiform anal papilla similar in length to lateral cirri (Figs 6D, 7A).

*Internal structures*. Muscular pharynx between segments 2 and 4. Eyes or nuchal organs not seen in holotype.

*Reproductive features.* Gametes or sexual structures not observed in holotype.

##### Variation.

Additional material measuring 1.0–3.5 mm in length and 0.33–0.37 mm wide; with 10–20 (usually 17–18) chaetigers. Live specimens unpigmented, with dorsum covered with small sediment particles, except for dorsal macrotubercles (Fig. 8A). Two oval eyes at level of median antenna or chaetiger 1–2, in some specimens (Fig. 8A). Stalk of dorsal macrotubercle is as long as the tubercle but in many specimens is contracted and therefore seems much shorter (e.g., Fig. 6C vs 6D). Usually, up to seven ventral stalked papillae in mid-body chaetigers but first two and last 2–3 chaetigers may show six (rarely five) papillae. Some specimens show the inter-parapodial tubercle displaced to parapodial areas. Ventral parapodial papilla usually appears in chaetiger 3–4 but in one specimen first appears in chaetiger 7. Otherwise, postchaetal papilla, lateral papilla, and acicular lobe appear constantly in chaetigers 1, 2, and 4 respectively. Muscular pharynx through segments 3–5. Genital openings distinguished in larger females and males (> 2 mm long) as swallowing of the tissue near the base of parapodia between chaetigers 8 and 9 (Figs 4D, 6E).

##### Etymology.

This species, originally described as *Sphaerodoridiumfauchaldi* was dedicated to our colleague and prolific annelid systematist Kristian Fauchald (Hartmann-Schröder, 1993). The new name given to it, after the genus *Sphaerodorum* is synonymised with *Clavodorum* and therefore the species is homonym to the previously described *Clavodorumfauchaldi* Desbruyères, 1980, aims to maintain tribute to Kristian, and therefore *kristiani* is proposed.

##### Remarks.

The present diagnosis is based in the original description of *Sphaerodoridiumfauchaldi* by Hartmann-Schröder (1993), additional observations (Aguirrezabalaga and Ceberio 2005, Moreira and Parapar 2007) and examination of the holotype, and several specimens from NW Spain and Nordic Seas. This species was described based on one small specimen (1.0 mm long, Fig. 2D, E); several minor differences were reported in material from the Bay of Biscay and NW Spain (Aguirrezabalaga and Ceberio 2005, Moreira and Parapar 2007) but these can be due to the size and state of maturity of the holotype. For instance, Hartmann-Schröder (1993)did not mention the presence of a ventral parapodial papilla, that is present in specimens reported by Aguirrezabalaga and Ceberio (2005) and Moreira and Parapar (2007) and in those examined in this study; however, this papilla could have been mistaken with a ventral small tubercle by Hartmann-Schröder (1993), and in fact one ventral papilla seems half-drawn in the original description (Hartmann-Schröder 1993: Fig. 8). Otherwise, this species is well characterized and can easily be distinguished from other *Clavodorum*-*Sphaerodoridium* species from the NE Atlantic, based on the number and arrangement of small ventral tubercles and lack of additional epithelial papillae.

##### Distribution.

We are reporting the species for the first time for the Norwegian Sea and Morocco. Previous records of the species include: North Sea (Hartmann-Schröder 1993); Bay of Biscay (Aguirrezabalaga and Ceberio 2005); NW Iberian Peninsula (Moreira and Parapar 2007, Moreira et al. 2011).

##### Habitat.

Continental shelf, sandy sediments (70–1000 m) (Moreira et al. 2011, and present study).

#### Commensodorum

Taxon classificationAnimaliaPhyllodocidaSphaerodoridae

Fauchald, 1974

Commensodorum Fauchald, 1974: 265–266; Capa et al. 2014: 16.

##### Type species.

*Sphaerodoridiumcommensalis* Lützen, 1961.

##### Diagnosis.

Body ellipsoid. Head with a median and a pair of lateral antennae; antenniform papillae absent; all appendages short. Tubercles sessile, conical, or pear-shaped, in four longitudinal rows, one transverse row per segment, except for first chaetiger with only two. Minute epithelial papillae on dorsal and ventral surfaces, in ca. 5–6 transverse rows per segment. Parapodia with rounded and small ventral cirri, not surpassing tip of acicular lobe. Stout hooks in anterior chaetigers absent. All chaetae simple, unidentate chaetae, enlarged subdistally, with serrated edge.

##### Remarks.

Referring to the main dorsal tubercles in *Commensodorum* as macrotubercles (e.g., Fauchald 1974) is herein avoided due to their smaller size compared to other members of the family. Members of this group have not been included in molecular analyses due to absence of material properly fixed for DNA extraction and sequencing and therefore relationships with other sphaerodorids remains unknown. The number of longitudinal rows of dorsal tubercles resembles the arrangement of macrotubercles in members of *Sphaerephesia* (as redefined in this study). However, tubercles in *Commensodorum* are smaller than in any other sphaerodorid. Shape of simple chaetae somehow resembles those of *Euritmia*, but they are thinner in the only species described in the genus, *Commensodorumcommensalis* (Lützen, 1961).

The genus is monotypic.

*Commensodorumcommensalis* (Lützen, 1961).

Type locality: Gullmarfjord, Sweden, 35 m.

#### Commensodorum commensalis

Taxon classificationAnimaliaPhyllodocidaSphaerodoridae

(Lützen, 1961)

Figs 4E, F , 5C , 9

Sphaerodoridium commensalis Lützen, 1961: 409–416, fig. 1.

##### Type locality.

Blåbergsholmen Island, Gullmarfjord, Sweden, 35 m.

##### Material examined.

**Holotype**: ZMUC-POL-1984, Sweden: Gullmarfjord, Blåbergsholmen Island, 35 m, 30 Oct 1960.

##### Additional material.

(2 specs) **Skagerrak**, NTNU-VM 73780 (1 spec. on SEM stub) (1 spec.), Lindön, 58°47.90'N, 11°09.52'E, 46 m, 7 May 2008. ZMBN 127260 (1 spec.), Tvedestrand, 58°33.929'N, 9°0.215'E, 34 m, 27 May 2011.

##### Diagnosis.

Body ellipsoid, up to 2.5 mm long. Head with short appendages, without spurs or basal papillae; antenniform papillae absent. Tubercles sessile, conical or pear-shaped, small, arranged in four longitudinal rows on dorsum, one transverse row per segment; except for first chaetiger with only two. Additional epithelial papillae minute over dorsal and ventral surfaces, in ca. 5–6 transverse rows per segment. Parapodia lacking papillae, acicular lobe, and ventral cirrus small and ellipsoid. Stout hooks in anterior chaetigers absent. All chaetae simple, unidentate, with broadened distal end, and serrated edge.

##### Re-description of holotype.

**Figure 9.**
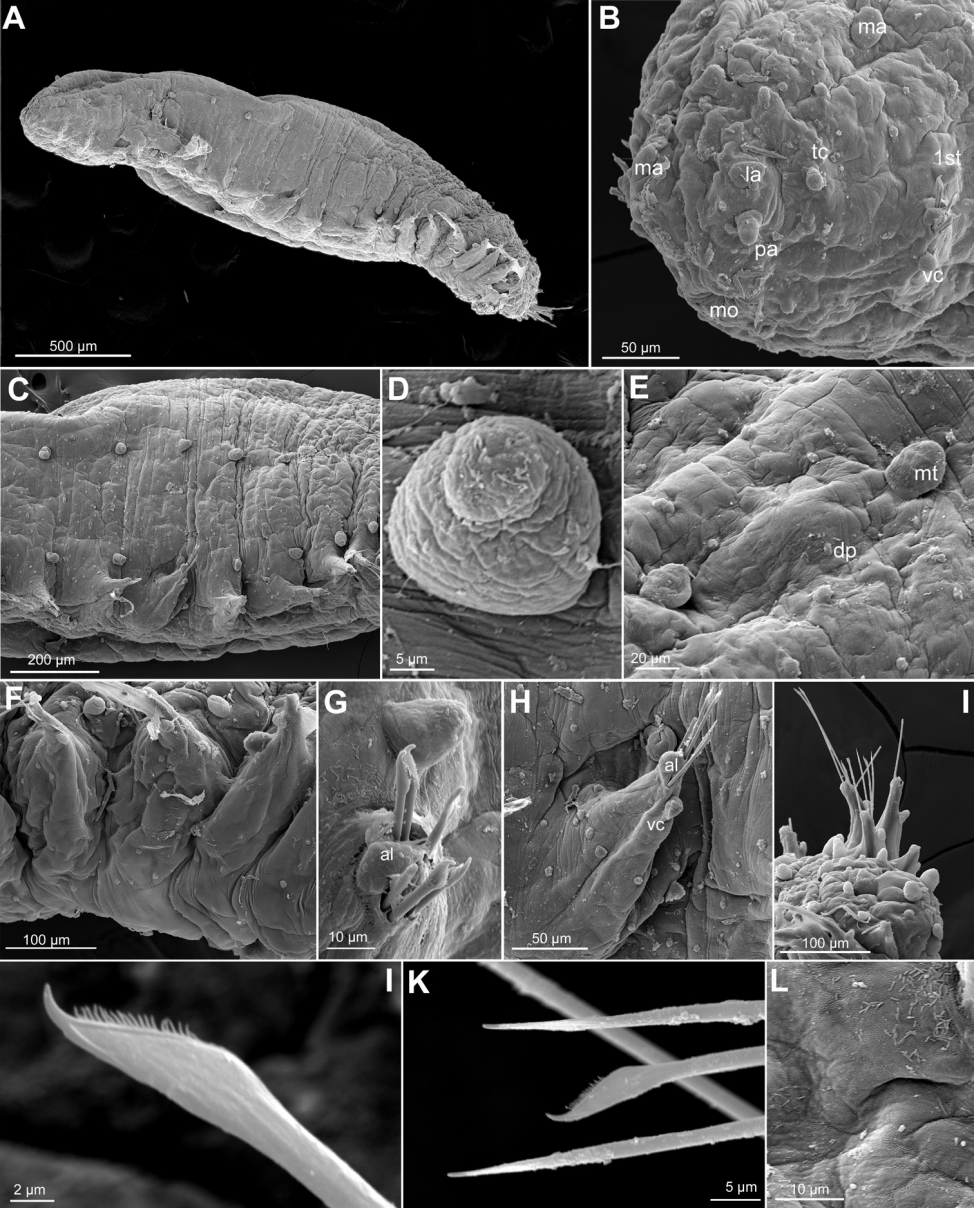
*Commensodorumcommensalis* (Skagerrak, NTNU VM 73780). **A** Complete specimen, lateral view **B** anterior end, lateral view **C** mid-body chaetigers, lateral view **D** dorsal tubercle, mid-body chaetiger, detail **E** epithelium between dorsal tubercles, detail **F** parapodia, chaetigers 12–14, lateral view **G** parapodium, anterior chaetiger, lateral view **H** parapodium, mid-body chaetiger, anterior view **I** parapodium, posterior chaetiger **J, K** simple chaetae, anterior and posterior chaetigers **L** epithelium, detail, showing granules.

*Measurements and general morphology.* Holotype 2.5 mm long, 0.7 mm wide, with 17 chaetigers; body ellipsoid, with convex dorsum and flattened ventrum; segmentation slightly noticeable, especially on ventral side (Fig. 9A, F). Pigmentation absent on preserved material.

*Head*. Head fused to first chaetiger (Fig. 9A, B). Prostomial appendages conical, slightly longer than wide. A pair of palps, bigger than lateral and median antennae (Fig. 9B). Dorsal antenniform papillae absent. Few small hemispherical papillae scattered on head surface (only noticeable under SEM, Fig. 9B). Tentacular cirri ellipsoid, smaller than palps, similar in size and shape to median antenna (Fig. 9B). Eyes not observed.

*Tubercles*. Dorsum with four longitudinal rows of larger tubercles; in one transverse row per segment (Figs 5E, 9A, C). First chaetiger only with two tubercles. Distance between dorsalmost rows larger than there and lateral rows of tubercles. Tubercles sessile, ellipsoid or pear shaped (Fig. 9C–E), with some pores on distal end (Fig. 9D). Dorsal papillae minute, hemispherical, arranged in ca. six transverse rows per segment (Figs 4E, 9C, E). Ventrum with fewer and larger papillae, more abundant near parapodial bases, than in mid-ventral line; with no clear arrangement pattern (Figs 4F, 9F).

*Parapodia*. Parapodia conical, as long as wide in three or four anterior chaetigers, 2–3 times longer in medium and posterior chaetigers. Acicular lobes ellipsoid, from first chaetiger (Fig. 9G, H). Ventral cirri similar in shape and size as acicular lobe (Fig. 9H). Parapodial papillae absent (Fig. 5C).

*Chaetae*. All parapodia with 4–5 simple chaetae (Fig. 9G–I); blade serrated on cutting edge and slight recurved distal tip; distal framed by thickened edges (Fig. 9I–K). One straight acicula per parapodium. Large, recurved hooks in the first chaetiger absent.

*Pygidium*. Pygidium with two dorsolateral and one mid-ventral ellipsoid cirri, similar in size.

*Reproductive features.* Holotype, gravid female, with oocytes measuring ca. 200 µm. Largest specimen also with oocytes, but smaller. Sexual structures or genital openings not observed.

##### Variation.

Largest specimens examined 4 mm long, 0.9 mm wide and 22 chaetigers. Smallest specimens with 19 chaetigers, 0.5 mm long. General morphology is homogenous among material studied. All specimens bear short prostomial appendages, ellipsoid or pear-shaped dorsal tubercles and minute epithelial papillae barely noticeable under stereomicroscope. One specimen (ZMUB 127260) is a gravid female with spheroid eggs occupying most of the body. Genital openings not observed in specimens examined.

##### Remarks.

Epithelium described as transparent in live specimens (Lützen 1961), allowing the observation of the nuchal organs (as pharyngeal glands) and the coiled gut. These are not visible in currently preserved specimens, with opaque epithelium. Original drawings of specimen with 22 chaetigers, does not correspond with the holotype. The body shape is ellipsoid, with blunt anterior and posterior ends, unlike the original description with tapering anterior end, probably due to contraction of specimens after fixation. Head appendages are short and ellipsoid in all specimens examined, not digitiform as in original description (Lützen 1961), also probably due to contraction. The main dorsal tubercles, four per chaetiger, were described as clavate (‘forme de massue’), additionally other mid-dorsal ‘capsules’ were described in anterior chaetigers, but they were not spotted in the material examined. Additional minute papillae are displayed forming ca. six transverse rows per segment.

*Commensodorumcommensalis* differs from other sphaerodorids in the presence of a unique combination of morphological features: four longitudinal rows of small dorsal tubercles, arranged in a single transverse row per segment, and the presence of simple chaetae. Sphaerodorids with simple chaetae comprise members of *Euritmia*, including the recently synonymised *Amacrodorum* (Capa et al. 2016b), lacking large epithelial tubercles but with epithelium covered with small papillae. In addition, members of *Sphaerodorum* typically bear simple chaetae, but macrotubercles are arranged in two longitudinal rows and have clearly defined terminal papillae, absent in *Commensodorum*, and bear in addition two longitudinal rows of dorsal microtubercles (small tubercles also provided with a terminal papilla).

##### Distribution.

Skagerrak, ? United Kingdom (Lützen 1961, Howson and Picton 1997, Frid et al. 2009, present study).

##### Habitat.

Originally described as commensal of *Terebellidesstroemii* Sars, 1835 (Lützen 1961), at 35 m. The species has only a few times been reported in the literature since and is not necessarily associated exclusively with *Terebellides* (Frid et al. 2009).

#### Euritmia

Taxon classificationAnimaliaPhyllodocidaSphaerodoridae

Sardá-Borroy, 1987

Euritmia Sardá-Borroy, 1987: 48; Moreira 2012: 41; Capa et al. 2014: 16; Capa et al. 2016a: 9.
Amacrodorum Kudenov, 1987: 917–918.

##### Type species.

*Euritmiahamulisetosa* Sardá-Borroy, 1987

##### Diagnosis.

Body short and ellipsoid. Head with short appendages, without spurs or basal papillae; antenniform papillae absent. Small tubercles or papillae spherical, sessile, smooth, without a terminal papilla, scattered over body surface and parapodia with apparent random distribution (over eight dorsal irregular longitudinal rows, and three or more transverse rows). Parapodia with short, rounded, ventral cirri, not surpassing the tip of acicular lobe. Stout hooks in anterior chaetigers absent. All chaetae simple unidentate, enlarged subdistally, with serrated edge.

##### Remarks.

The genus *Euritmia* was erected to gather sphaerodorids with tubercles scattered over the dorsum and simple chaetae, differing in morphology from chaetae present in other sphaerodorids (Sardá-Borroy 1987, Capa et al. 2014) (i.e., *Sphaerodorum* and *Commensodorum*). The lack of morphological differences across members of *Euritmia* and *Amacrodorum*, a genus erected in the same year (Kudenov 1987) concluded in the recent synonimization of *Amacrodorum* (Capa et al. 2016b). The presence of the characteristic simple chaetae is also shared by species of *Sphaerodoropsis* species belonging to the informal Group 4, sensu Borowski (1994), whose dorsal tubercles are also small compared to other sphaerodorids, but similar to those present in *Euritmia*, and arranged in several transverse rows per segment. No sequences of members of *Euritmia* or this group of *Sphaerodoropsis* have been produced to date to assess their relationships and position within the sphaerodorid tree, but it will be most interesting to test if these two groups (*Euritmia* and *Sphaerodoropsis* Group 4) are closely related. Members of this group of *Sphaerodoropsis* are not reported in the NEA, or found in the present study.

The species currently considered in the genus are:

*Euritmiabipapillata* (Kudenov, 1987)

Type locality: Akutan Island, Alaska, 59 m.

*Euritmiacapense* (Day, 1963)

Type locality: Cape Town, South Africa, unknown depth.

*Euritmiacarolensis* Capa, Osborn & Bakken, 2016.

Type locality: Off South Carolina, USA, 799 m.

*Euritmiahamulisetosa* Sardá-Borroy, 1987.

Type locality: Cádiz, Spain, 0.5–10 m.

*Euritmianordica* sp. n.

Type locality: Greenland, Denmark Strait, 321 m.

#### Euritmia hamulisetosa

Taxon classificationAnimaliaPhyllodocidaSphaerodoridae

Sardá-Borroy, 1987

Figs 4G, H , 5D

Euritmia hamulisetosa Sardá-Borroy, 1987: 48–49, fig. 1, 2; Moreira 2012: 41–43, fig. 12F, 14.

##### Type locality.

Cádiz, South of Iberian Peninsula, 0.5–10 m.

##### Diagnosis.

Body short and ellipsoid, up to 0.6 mm long. Head with short appendages, without spurs or basal papillae; antenniform papillae absent. Epithelial papillae sessile, spherical, arranged in four transverse rows per segment. Ventrum with a pair of papillae near the parapodial bases and two additional longitudinal rows. Ventral papillae, in four transverse rows per segment. Microtubercles (small tubercles with a collar and terminal papillae) absent. Parapodia with a large dorsal papilla, digitiform acicular lobe, and spherical ventral cirrus. Stout hooks in anterior chaetigers absent. Six simple chaetae with serrated edge, enlarged subdistally, with a distal spine and filament in opposite directions.

##### Material examined.

No specimens were available for this study.

##### Remarks.

In the original description, the dorsal epithelial tubercles were termed macrotubercles (Sardá-Borroy 1987). These have later been interpreted as papillae due to their size and arrangement in comparison to other sphaerodorids (e.g., Capa et al. 2016b). The parapodia were originally described with double parapodial lobes and a small globular cirrus (Sardá-Borroy 1987). These were interpreted differently as it was clear the large lobes represent the acicular lobe and ventral cirrus, and ventral papillae placed at the base of parapodia (Fig. 5D; Capa et al. 2014).

*Euritmiahamulisetosa* is distinguished from other congeners by the unique combination of two features: the arrangement of dorsal papillae in four transverse rows per segment (Fig. 4G) and the presence of parapodial papillae on the anterior and posterior surfaces (Fig. 5D). *Euritmiacapense* (Day, 1963) bears two transverse rows of papillae per segment and five parapodial papillae (one dorsal, one ventral and three smaller ones on anterior and posterior parapodial surfaces). *Euritmiabipapillata* (Kudenov, 1987) has three transverse rows of dorsal papillae and one papilla on anterior surface of parapodia; *Euritmiacarolensis* (Capa, Osborn & Bakken, 2016) has three transverse rows of papillae and no parapodial papillae (Capa et al. 2016b). *Euritmianordica* sp. n. bears three transverse rows per segment (with a characteristic longitudinal mid-dorsal bare area) and no papillae on parapodia (present study).

##### Distribution.

Gibraltar Strait and Mediterranean coast of the Iberian Peninsula (Sardá-Borroy 1987, Moreira 2012).

##### Habitat.

Littoral algae and dentritic bottoms to 10 m (Moreira 2012).

#### Euritmia nordica

Taxon classificationAnimaliaPhyllodocidaSphaerodoridae

Capa & Bakken sp. n.

http://zoobank.org/77E0A063-122F-46C9-93CB-E7DDD04DA297

Figs 4I–J , 5E , 10

##### Type locality.

Greenland Sea, off eastern Greenland in Denmark Strait, 321 m.

##### Material examined.

**Holotype**: SMF 25281, Greenland, Denmark Strait, 67°38.77'N, 26°44.78'W, 321 m, 14 Sep 2011. **Paratype**: ZMBN 127261 (1 spec. on SEM stub), Norwegian Sea, 63°2.232'N, 4°41.34'E, 760 m, 30 Sep 2013.

##### Diagnosis.

Body short and ellipsoid, up to 1.5 mm long. Head with short appendages, without spurs or basal papillae; antenniform papillae absent. Dorsum with sessile spherical papillae arranged in three transverse rows, and up to 18, per segment. Ventrum with a pair of papillae near each parapodial bases and two additional longitudinal rows. Parapodia without papillae; digitiform acicular lobe and spherical ventral cirrus. Six or seven simple chaetae with serrated edges, enlarged subdistally, with a distal spine and filament in opposite directions.

##### Description.

**Figure 10.**
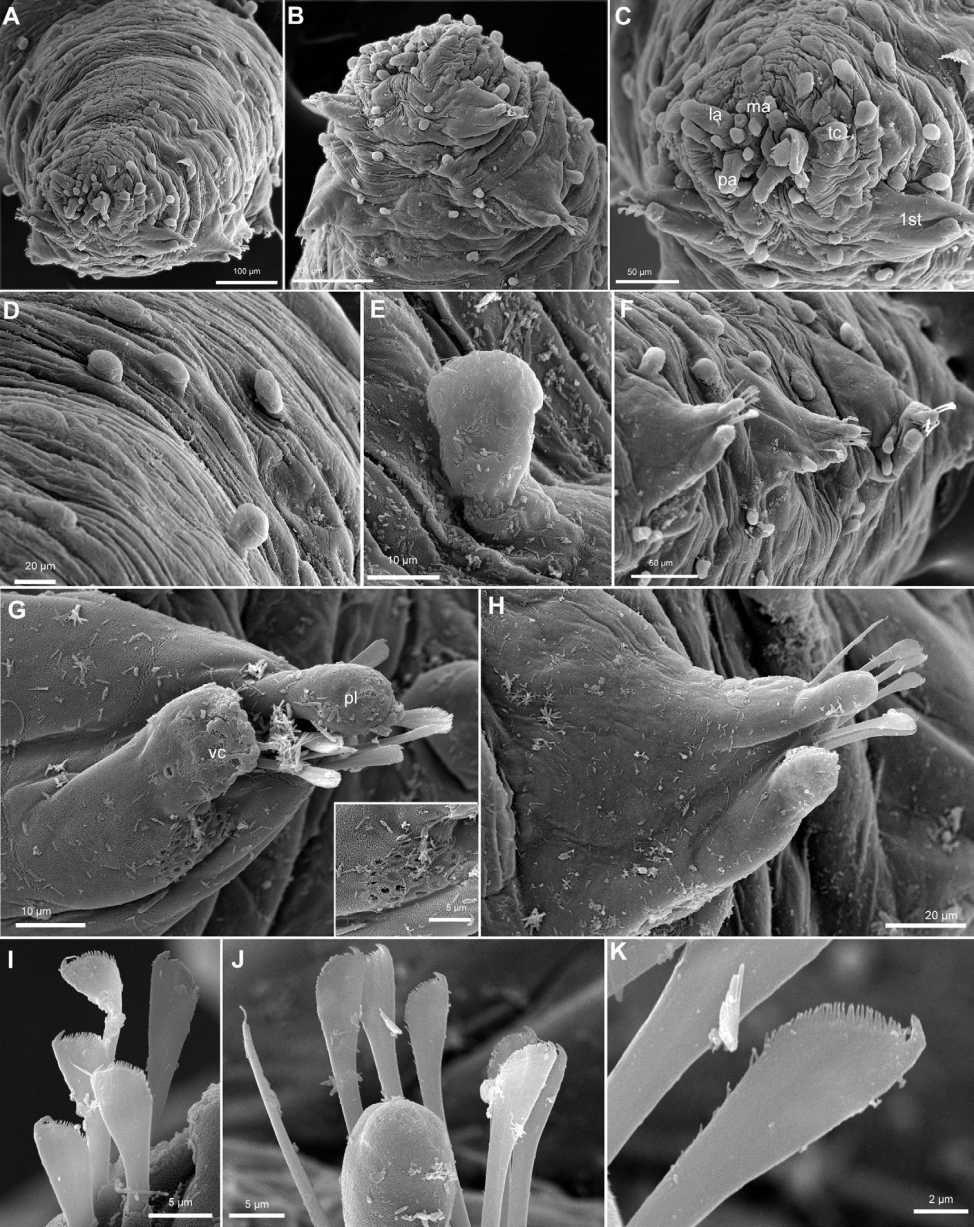
*Euritmianordica* sp. n., paratype (ZMBN 127261), scanning electron micrographs. **A** Anterior end, frontal view **B** anterior end, ventral view **C** anterior end with head appendages, frontal view **D**&nbsp;epithelial dorsal papillae, chaetiger 3, detail **E** dorsal papilla, chaetiger 2 **F** parapodia, left side, chaetigers 3–5, anterior view **G** parapodium, left side, chaetiger 1, anterior view (insert: detail of pores ventral to ventral cirrus) **H** parapodium, left side, chaetiger 2, anterior view **I** simple chaetae, anterior chaetiger **J&nbsp**;simple chaetae, anterior chaetiger **K** simple chaetae, detail of distal spines on blade.

*Measurements and general morphology*. Holotype 0.9 mm long, 0.5 mm wide, with 12 chaetigers. Body ellipsoid, with strongly convex dorsum and flattened ventrum (Fig. 10A). Epithelium with transverse wrinkles, segmentation not noticeable (Fig. 10A, D). Pigmentation absent on preserved material.

*Head*. Head fused to first chaetiger (Fig. 10B, C). Prostomial appendages digitate, with a pair of palps, a pair of lateral antennae slightly longer than palps, a median antenna half as long as lateral antennae (Fig. 10B, C). Dorsal antenniform papillae absent or not conspicuous. Few small hemispherical papillae scattered on head surface, ca. 5–6 confined among palps and antennae. Tentacular cirri, digitate, shorter than palps, similar in size and shape to median antenna (Fig. 10C). Eyes not observed.

*Tubercles*. Dorsum with about eight rows of similar sized and ellipsoid papillae, in three irregular transverse rows of per segment (Fig. 10A, D–F); adding up to ca. 15–18 per segment on mid-body chaetigers (Fig. 4I). Ventrum with fewer spherical papillae, arranged at base of parapodia with two parallel papillae (Fig. 10B, F), and additional two longitudinal rows (10B, C), in total ca. six papillae per segment (Figs 4J, 10B, C). Mid-dorsal body bare of papillae (Fig. 10B).

*Parapodia*. Parapodia conical, as long as wide in all chaetigers (Fig. 10F, H), similar throughout. Acicular lobe digitate longer than ventral cirri; ventral cirri ellipsoid (Fig. 10F–H). Most segments with no additional papillae or parapodial appendages, exceptionally one or two small parapodial papillae near the base of ventral cirrus (Fig. 5E, 10F).

*Chaetae*. Large, recurved hooks in the first chaetiger absent. All parapodia with 6–7 simple chaetae; blade serrated on cutting edge and a recurved distal tip (Fig. 10C, G–K).

*Pygidium*. Pygidium with two small and spherical tubercles and a small digitiform ventral cirrus.

*Internal features*. A pair of rounded eyes observed under the epithelium, in the head. Muscular pharynx not observed.

*Reproductive features*. Two large eggs are visible in the coelom of the holotype.

##### Variation.

Paratype 1.5 mm long, 0.6 mm wide, with 19 chaetigers (anterior end on SEM stub). Papillae on ventrum in three transverse rows per segment, positioned as two parallel at base of parapodia and one towards mid-body (Fig. 10B). Parapodia are smooth in most chaetigers of both holo- and paratype but variation has been observed, with one parapodium with one small papilla and the following with two papillae, located at the base of the ventral cirrus (Fig. 10F). Sexual structures (unknown gender) observed as inflated ventral cirri with pore openings in first chaetiger in the paratype (Figs 4J, 10G).

##### Etymology.

The epithet of this species, *nordica*, refers to the geographical region where this species has currently been found. Nordic or The North gathers the north-western European countries, including Scandinavia and Fennoscandia.

##### Remarks.

*Euritmianordica* sp. n. is characterized by a unique combination of features: dorsum provided with similar sized, ellipsoid tubercles arranged in three transverse rows per segment, leaving a conspicuous longitudinal bare mid-dorsal area; two longitudinal zig-zag rows of small and ellipsoid papillae on ventrum (with ca. six papillae per segment); parapodia lacking papillae (with some exceptions). *Euritmianordica* sp. n. resembles *E.carolensis* due to the arrangement of dorsal papillae in three transverse rows per segment and the absence of parapodial papillae. Differences between members of these two species rely on size and number of dorsal epithelial papillae, larger and more abundant in *E.carolensis* (covering most of the dorsum, while in *E.nordica* sp. n. the dorsum is mainly smooth); chaetae in *E.nordica* sp. n. are broader subdistally and recurved at the distal end, while *E.carolensis* has a slim appearance and a straighter distal tip. Moreover, *E.nordica* sp. n. presents an inflated cirrus in the first chaetiger, with pore openings on its base (Fig. 10G), similar to what was described in *Sphaerodoropsishalldori*, on chaetiger 6 of males (Moreira and Parapar 2012). This is the first time that potentially sexual structures are described in members of *Euritmia*, and therefore it is unknown if the attributes described herein are species specific.

*Euritmianordica* sp. n. is distinguished from *E.bipapillata*, described from Alaska, in the absence of parapodial papillae, while *E.bipapillata* has one papilla on anterior surface of parapodia (Kudenov, 1987). *Euritmiahamulisetosa* from southern Spain has dorsal papillae in four transverse rows per segment and parapodial papillae (Sardá-Borroy 1987), unlike *E.nordica* sp. n. with three transverse rows of dorsal papillae and smooth parapodia. *Euritmiacapense*, from South Africa, bears two transverse rows of papillae per segment and five parapodial papillae (Day 1963).

##### Distribution.

The species is known from Denmark Strait, East of Greenland (holotype), and the Norwegian Sea.

##### Habitat.

The specimens were found in soft bottom, in areas influenced with cold water (approximately 0 °C). The paratype was found inside the tube of an undescribed species of *Ampharete*.

#### Geminofilum gen. n.

Taxon classificationAnimaliaPhyllodocidaSphaerodoridae

http://zoobank.org/35550293-C24E-44D9-8748-D56C437BCB97

Sphaerodoropsis Hartman & Fauchald, 1971: 69 (in part); Fauchald 1974: 261 (in part); Borowski 1994: 23 (in part); Moreira 2012: 30 (in part); Capa et al. 2014: 18 (in part).

##### Type species.

*Sphaerodorumdistichum* Eliason, 1962.

##### Diagnosis.

Body short and cylindrical. Head with a median and a pair of lateral antennae; antenniform papillae absent or present; all appendages short. Tubercles sessile, spherical or hemispherical, arranged in two transverse rows per segment. Additional epithelial papillae on dorsal (sometimes absent) and ventral surfaces. Parapodia with elongated ventral cirri, as long as acicular lobe. Stout hooks in anterior chaetigers absent. All chaetae compound, unidentate, with serrated edge.

##### Remarks.

Analyses of molecular data presented here reveal that members of previously considered *Sphaerodoropsis* Group 3, according to Borowski (1994), form a monophyletic group, morphologically quite distinct (with dorsal macrotubercles, arranged in two transverse rows per segment and genetically (long branch compared to other clades; Fig. 1). This clade is sister to *Sphaerephesiasensu stricto* in the present study (sphaerodorids with four dorsal longitudinal rows of sessile macrotubercles). Since the type species of *Sphaerodoropsis, Sphaerodoropsissphaerulifer* does not possess the main diagnostic features of this clade, and instead bears dorsal macrotubercles in a single transverse row (e.g., Moore 1909, Kudenov 1987), the erection of a new genus for accommodating these other species is needed. *Geminofilumdistichum* (Eliason, 1962), comb. n., is the selected type species of the new genus because it is the oldest described species in the group that has been included in the present molecular analyses.

It is here assumed that *Geminofilum* gen. n. includes all sphaerodorids presenting two transverse rows of macrotubercles, but confirmation of this hypothesis is needed, since several of the species with this morphological feature have not been included in the analyses. *Geminofilum* gen. n. would therefore be represented by the following 15 species, all of which require nomenclatural changes:

*Geminofilumarctowskyensis* (Hartmann-Schröder & Rosenfeldt, 1988), comb. n.

Type locality: South Shetland Islands, Antarctica, 265 m.

*Geminofilumbisphaeroserialis* (Hartmann-Schröder, 1974), comb. n.

Type locality: South of Durban, South Africa, 20 m.

*Geminofilumdistichum* (Eliason, 1962), comb. n.

Type locality: Skagerrak, North East Atlantic, 460 m.

*Geminofilumfauchaldi* (Hartmann-Schröder, 1979), comb. n.

Type locality: Pt. Hedland, Western Australia, shallow depth.

*Geminofilumgarciaalvarezi* (Moreira, Cacabelos & Troncoso, 2004), comb. n.

Type locality: Baiona, NW Spain, 7 m.

*Geminofilumhalldori* (Moreira & Parapar, 2012), comb. n.

Type locality: Western Iceland, 1162 m.

*Geminofilumheteropapillatum* (Hartmann-Schröder, 1987), comb. n.

Type locality: Geelong, Victoria, Australia, shallow depth (coralline algae).

*Geminofilummultipapillatum* (Hartmann-Schröder, 1974), comb. n.

Type locality: Mtwara, Tanzania, shallow depth (in coral reef).

*Geminofilumoculatum* (Fauchald, 1974), comb. n.

Type locality: Antarctic Peninsula, 412 m.

*Geminofilumparacapense* (Hartmann-Schröder, 1974), comb. n.

Type locality: Diaz Point, Namibia, SW Africa, unknown depth.

*Geminofilumpycnos* (Fauchald, 1974), comb. n.

Type locality: Antarctic Peninsula, 650 m.

*Geminofilumsexantennellum* (Kudenov, 1993), comb. n.

Type locality: Southern California, ca. 150 m.

*Geminofilumsolis* (Reuscher & Fiege, 2015), comb. n.

Challenger Plateau, Tasman Sea, 1523–1526 m.

*Geminofilumspissum* (Benham, 1921), comb. n.

Type locality: Macquarie Island, Southern Ocean, unknown depth.

*Geminofilumtranslucidum* (Borowski, 1994), comb. n.

Type locality: Peru Basin, 4162 m.

##### Etymology.

The name of this genus, *Geminofilum*, refers to the particular organization of macrotubercles in members of this genus in double (*Geminus* in Latin, gender: masculine) rows (*filum*, in Latin, gender: neuter).

#### Geminofilum distichum

Taxon classificationAnimaliaPhyllodocidaSphaerodoridae

(Eliason, 1962) comb. n.

**Figure 11.**
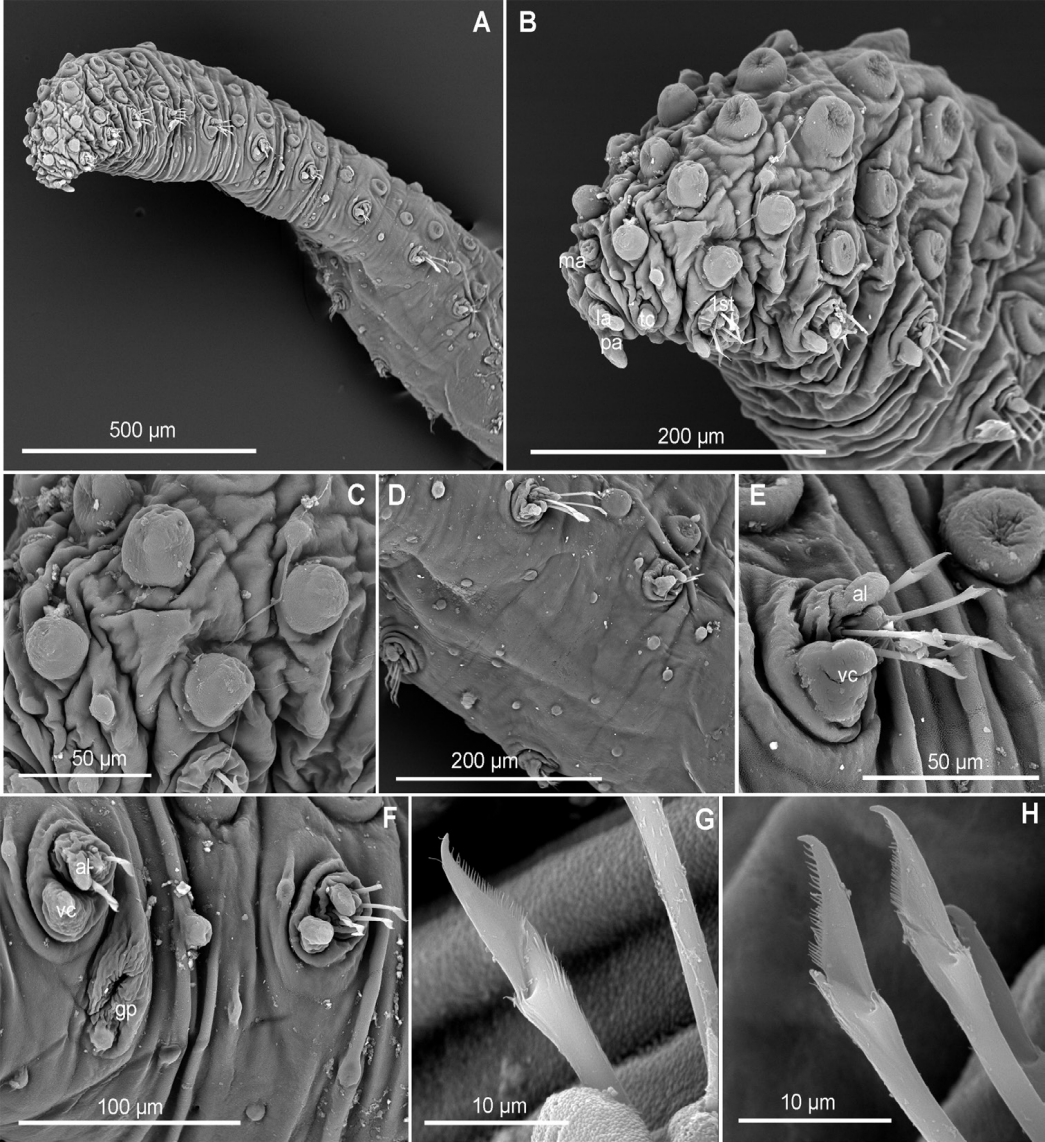
*Geminofilumdistichum* comb. n. (ZMBN 127263), scanning electron micrographs. **A** Anterior end, lateral view **B** head and anterior segments, dorsal view **C** dorsal macrotubercles, anterior chaetigers **D** chaetigers 9 and 10, ventral view **E** parapodium, chaetiger 5, anterior view **F** genital opening between parapodia of chaetiger 6 and 7 **G** chaeta, chaetiger 5 **H** chaetae, mid-body chaetiger.

**Figure 12.**
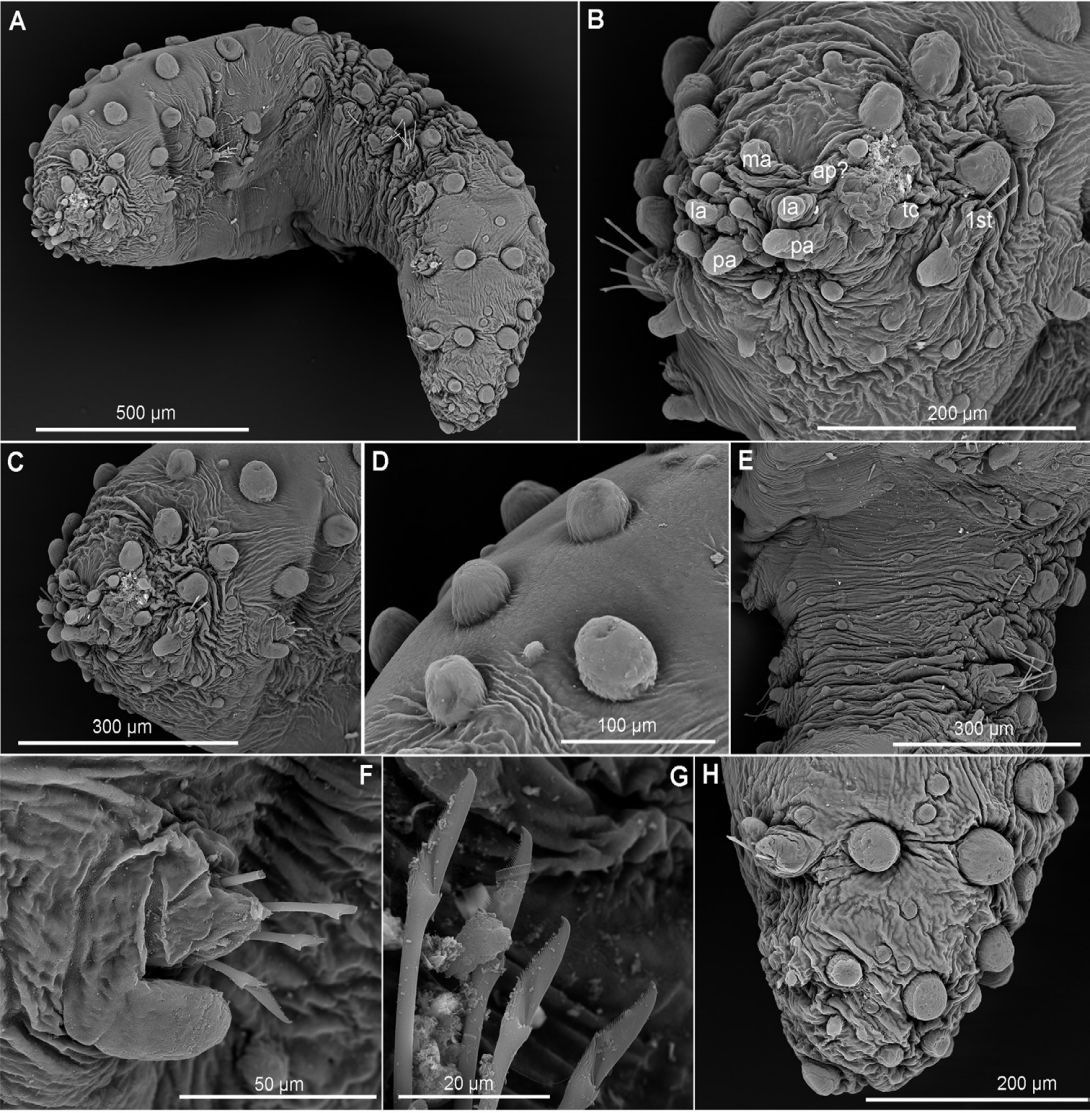
*Geminofilumdistichum* comb. n. (SMF 23898), scanning electron micrographs. **A** complete specimen, lateral view **B** head, frontal view **C** head and anterior segments, lateral view **D** dorsal macrotubercles, chaetiger 2 **E** mid-body chaetigers, ventral view **F** parapodium, chaetiger 2, anterior view **G**&nbsp;chaetae, mid-body chaetigers **H** posterior end and pygidium, lateral view.

Figs 4K–N , 5F , 8B–C , 11 , 12

Sphaerodorum distichum Eliason, 1962: 247–248, fig. 12.
Sphaerodoropsis distichum (Eliason, 1962). Hartmann-Schröder 1996: 236.
? Sphaerodoropsischardyi Desbruyères, 1980: 115–117, fig. 4; ? Böggemann 2009: 388–389, fig. 120, 123, 124, 137.

##### Type locality.

Skagerrak, 58°05'N, 8°32'E, 460 m.

##### Material examined.

**Holotype**: UUZM 203, Skagerrak, 58°05'N, 8°32'E, 460&nbsp;m, 4 July 1933.

Holotype of *Sphaerodoropsischardyi*: MNHN TYPE 1282, Bay of Biscay, 44°11.3'N, 4°15.4'W, 2430 m.

##### Additional material.

(6 specs) **Iceland**: DZMB-HH 28574 (1 spec. used for DNA sequencing, SPH 294), South Iceland, Iceland Basin, 62°33.50'N, 020°21.18'W, 1390 m, 02 Sep 2011; SMF 23898 (1 spec., used for SEM and DNA sequencing, SPH 064), Iceland Basin, 62°33.1'N, 20°23.71'W, 1384 m, 2 Sep 2011; SMF 23899 (1 spec. used for DNA sequencing, SPH 049) South Iceland, Irminger Basin, 61°36.19'N, 031°22.60'W, 2537 m, 07, Sep 2011. **Norwegian Sea**: ZMBN 127262 82.11.27.1 (1 spec.), 62°59.1'N, 3°13.1'E, 804 m, 27 Nov 1982; **Skagerrak**: ZMBN 127263 (1 spec. used for SEM and DNA sequencing, SPH295, photographed alive Fig. 8B), Drøbak, 59°38.664'N, 10°37.152'E, 106 m, 24 Oct 2014.

##### Diagnosis.

Body short and cylindrical, up to 2.5 mm long. Prostomial appendages smooth, lacking spurs or basal papillae. Dorsal macrotubercles sessile, hemispherical, arranged in two transverse rows per segment, with five and six macrotubercles each, from segment 2. Dorsum with 4–6 additional papillae per segment in mid body. Ventrum with 6–8 hemispherical papillae per segment, arranged in nearly Ʌ-shaped. Females with a pair of large ventral papillae, or sexual structures, between chaetigers 6 and 7. Parapodia without papilla. Acicular lobe from chaetiger 2. Stout hooks absent in anterior chaetigers. Compound chaetae in all parapodia, 4–7, with short blades (up to four times as long as wide).

##### Re-description of holotype.

*Measurements and general morphology.* Holotype short and cylindrical, 2.2 mm long and 0.4 mm wide, with 16 chaetigers. Dorsum convex, ventrum flattened. Segmentation inconspicuous, tegument smooth. Live specimens with some whitish macrotubercles (Fig. 8B); preserved specimen lacking pigmentation.

*Head.* Anterior end bluntly rounded. Prostomium and peristomium indistinct, appendages not observed in holotype, due to contraction of specimen. Additional material with small and digitiform prostomial appendages, without spurs or basal papillae (Figs 11A, B, 12A–C). Antenniform papillae could be considered present (due to the position behind lateral antennae), but similar in shape and size to other prostomial papillae (Fig. 12B). Tentacular cirri, similar in size and shape to prostomial appendages. Either five or six rounded prostomial papillae between antennae (Figs 11B, 12B).

*Tubercles.* Dorsal macrotubercles sessile and ovoid (Figs 11C, 12D). First chaetiger with two anterior macrotubercles and a posterior with four (Figs 11B, 12B). Following chaetigers with double transverse rows of macrotubercles per segment, with five and six tubercles on each of the anterior and posterior rows, arranged in zig-zag pattern; lateralmost tubercles smaller (Fig. 4K). Few additional hemispherical papillae scattered on dorsum in no clear pattern, maximum of five or six papillae on mid segments (Fig. 12A). Ventrum with 6–8 small, hemispherical papillae arranged in a more or less Ʌ-shaped, on each segment (Figs 4L, 11D, 12E). One hemispherical papilla between parapodia, larger than ventral papillae, forming one row on each side on some segments (Fig. 11A).

*Parapodia.* Parapodia sub-conical, slightly longer than wide. Chaetigers with digitiform acicular lobe, present from chaetiger 2, projecting as long as ventral cirrus (Fig.&nbsp;11B); ventral cirri bluntly rounded (Figs 11D–F, 12F). Parapodia lacking papillae (Figs 5F, 11D–F, 12F).

*Chaetae.* Compound chaetae present in all chaetigers, arranged in a straight or curved row posterior to acicular lobe, numbering 4–7 per fascicle (Fig. 11E, F). Shaft with slender distal end, blades slender, ranging 3–4 times longer than maximum width (Figs 11G, H, 12G).

*Pygidium.* Pygidium blunt with a pair of rounded terminal papillae (Fig. 11H).

*Internal features.* Eyes or muscular pharynx not seen in opaque holotype.

*Reproductive features.* Holotype female with few oblong eggs measuring 200 µm in length. A female with a flat tubercle (genital opening) between parapodia 6 and 7 (Figs 4L, 11F).

##### Variation.

Specimens studied measured between 1.5 and 2.5 mm long and 0.3–0.5 mm wide. Live specimens translucent with white spots in macrutubercles (Fig. 8B, C). Eyes seen (dark red) in one live specimen (Fig. 8B), pigmented (bright orange) nuchal organs observed in another similar specimen (Fig. 8C). Fixed specimens lacking any pigmentation pattern. Muscular pharynx, occupying ca. three segments, observed in translucent live specimens. All specimens identified as *Geminofilumdistichum* comb. n. bear a similar pattern of dorsal hemispherical-oval macrotubercles (5+6), on each midbody segment, but in one individual, and also in the holotype of *S.chardyi* the lateral most tubercles are larger than the mid-dorsal ones, contrary to the holotype and some other specimens with smaller macrotubercles near parapodia (Fig. 4K). This specimen also presented a slightly different arrangement of ventral papillae, more aligned into four longitudinal rows (Fig. 4L).

##### Remarks.

*Sphaerodoropsisdistichum* was described from 450 m depth in the Skagerrak (Eliason 1962), and has never been found again. The single specimen acknowledged, the holotype, is not in optimal condition and some of the features referred to in the original description (Eliason 1962) may be inaccurate. For example, the anterior appendages were reported as absent even if the specimen looked like presenting a contracted anterior end. Re-examination of the holotype indicates parapodia lack papillae (although reported as bearing one papilla in the posterior surface), and instead there seem to be some papillae over the ventrum of the specimen, that was reported as smooth, except for a longitudinal row near parapodia. Additional material collected nearby the type locality confirmed these new findings: the specimen presents head appendages, that are short and digitiform; parapodia do not bear papillae; dorsum has a few scattered papillae in addition to the hemispherical macrotubercles and ventrum has 6–8 small spherical papillae. With these changes, the description of *G.distichum*comb. n. resembles that of *S.chardyi* Desbruyères, 1980 described from the Bay of Biscay, ca. 2500 m depth. Differences would be the presence of a curved acicula in *S.chardyi*, a feature that is herein questioned as it has not been observed in any sphaerodorid. Moreover, Desbruyères (1980) most likely unintentionally overlooked the existence of *S.distichum* and did not compare both species. It is the first time that sexual structures are reported in members of these two species.

Additional individuals found at 1400 m in Iceland (Fig. 12) also match the description of *G.distichum* comb. n. It is therefore here proposed the synonymy of *S.chardyi* and *G.distichum* (with some caution), based on morphological similarity between the types and additional material reported herein. There is, however, considerable genetic difference between specimens collected at different localities and depths (Fig. 1) that may indicate a large population structure within the species, or else that we are dealing with a species complex with clear geographical or bathymetrical boundaries between them. Furthermore, this species was reported from the Angola Basin also lacking parapodial papillae, apart from a globular papilla near anterior base (Böggemann 2009). In order to assess the species boundaries of *G.distichum* comb. n., and the distribution range more material, and DNA sequences, will be needed.

*Geminofilumdistichum* comb. n. is clearly recognised from other congeners, by the arrangement of macrotubercles in the first segment (2+4), the scarce and randomly arranged additional papillae over dorsum and the lack of parapodial papillae.

##### Distribution.

This is the first record for this species in Iceland and the Norwegian Sea. It had previously been reported from Skagerrak and English Channel (Eliason 1962, Desbruyères 1980). Species reported also in Angola and Guinea Basins, 3900–5500 m (Böggemann 2009).

##### Habitat.

Sediments from 100 to 2500 m (at least) (Eliason 1962, Desbruyères 1980, and present study)

#### Geminofilum halldori

Taxon classificationAnimaliaPhyllodocidaSphaerodoridae

(Moreira & Parapar, 2012) comb. n.

**Figure 13.**
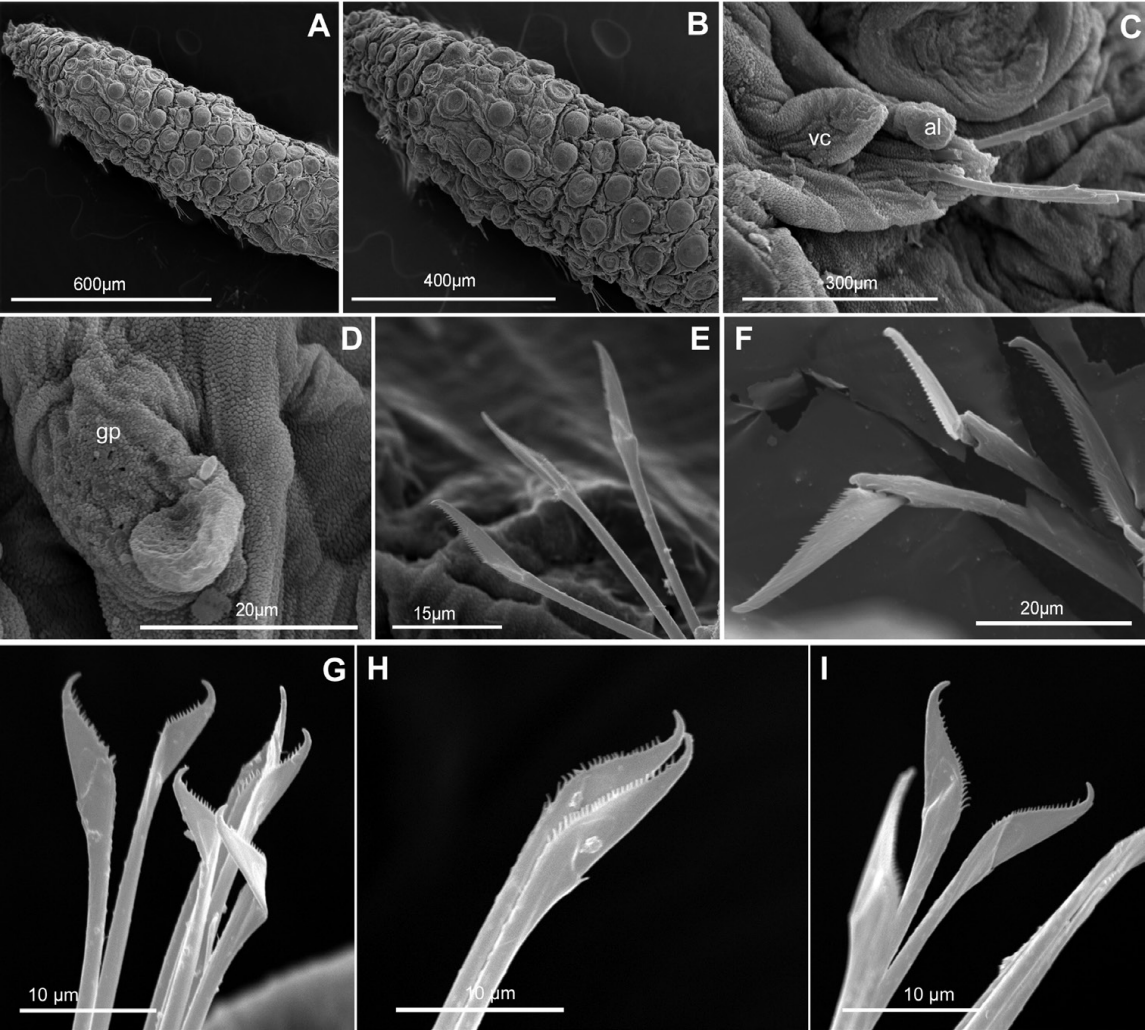
*Geminofilumhalldori* comb. n., scanning electron micrographs (IINH 38793). **A** anterior end, dorsal view **B** disposition of dorsal macrotubercles, anterior chaetigers **C** parapodium, mid-body chaetiger, ventral view **E, F** chaetae, mid-body chaetiger. *Geminofilumgarciaalvarezi*, scanning electron micrographs (MNCN 16.01/18460, NW Spain) **G–I** chaetae, mid-body chaetiger.

Figs 4O, P , 5G , 13A–F

Sphaerodoropsis halldori Moreira & Parapar, 2012: 588–591, figs 1B, 4–5, 6D–F.

##### Type locality.

West Iceland, 64°26'N, 28°15'W, 1162 m.

##### Material examined.

**Iceland** (13 specs): IINH 38791 (3 specs), 65°15.61'N, 28°50.15'W, 1300 m, 28 Aug 1996; IINH 38792 (8 specs), 65°11.01'N, 29°04.18'W, 1456 m, 25 Aug 1996; IINH 38793 (2 spec. on SEM stub), 62°23.15'N, 28°16.91'W, 1558 m, 7 Sep 2003.

##### Diagnosis.

Body short and cylindrical, up to 3.5 mm long. Prostomial appendages smooth, lacking spurs or basal papillae. Dorsal macrotubercles sessile, almost spherical, arranged in two transverse rows per segment, with six and seven macrotubercles each, from segment 3. Dorsum with seven additional rounded papillae per segment in mid body, arranged in seven longitudinal rows. Ventrum with up to eight papillae per segment in mid body, arranged in six longitudinal rows and forming a V on each segment. Females with a pair of larger tubercles in chaetigers 7–9. Parapodia with one papilla on anterior surface from chaetiger 3. Acicular lobe from chaetiger 1–2. Compound chaetae, 4–8, with short blades (up to five times as long as wide), showing some intra-fascicle variation in size.

##### Reproductive features.

Some males filled with sperm and females with oocytes. Sexual structures of males as ventral cirri basally inflated, and with pores on ventral surface on chaetiger 6. Females with pair of oval, distally opened tubercle located ventro-laterally to parapodia on chaetigers 6–7 (Fig. 4P); in addition, ventral cirri of chaetigers 4–7 basally inflated and with ventral pores.

##### Variation.

Size range (type series): 2.5–3.1 mm long, 0.4 mm wide, with 17–20 chaetigers. Pigmentation absent in fixed specimens.

##### Remarks.

*Geminofilumhalldori* comb. n. resembles *G.bisphaeroserialis* (Hartmann-Schröder, 1974), comb. n., *G.arctowskyensis* (Hartmann-Schröder & Rosenfeldt, 1988), comb. n., and *G.garciaalvarezi* (Moreira et al. 2004), comb. n. in the general appearance, number and arrangement of dorsal macrotubercles (Fig. 13A, B), and the presence of one papilla on the anterior surface of parapodia (Fig. 5G). However, they can be distinguished by the number and arrangement of dorsal and ventral papillae. *Geminofilumhalldori* comb. n. is characterised by presenting seven small and hemispherical papillae per segment from chaetiger 3 arranged in a transverse row between segments (Fig. 4O), and three in a row between parapodia (these features are not visible in Fig. 13, probably due to the contraction of animal and wrinkled epithelium). Chaetae show slight variation in size of blades within parapodia, but anterior chaetigers bear longer blades (Fig. 13E, F).

##### Distribution.

West Iceland (Moreira and Parapar 2012, present study).

##### Habitat.

Sandy sediments, at depths of 1162–1558 m (Moreira and Parapar 2012, this study).

#### Geminofilum garciaalvarezi

Taxon classificationAnimaliaPhyllodocidaSphaerodoridae

(Moreira, Cacabelos & Troncoso, 2004) comb. n.

Figs 4Q, R , 5H , 13G–I

Sphaerodoropsis garciaalvarezi Moreira, Cacabelos & Troncoso, 2004: 995–999, figs 1–3, 4A, D.

##### Type locality.

Ensenada de Baiona, NW Iberian Peninsula, 42°08.83'N, 8°50.25'W, 7 m.

##### Material examined.

(2 specs) **NW Spain**: MNCN 16.01/18460 (2 specs, on SEM stub), Ría de Ferrol, 08°14.37'N, 43°27.88'W, 15 m, 19 Jul 2010.

##### Diagnosis.

Body short and cylindrical, up to 2.5 mm long. Head appendages short, smooth, lacking spurs or basal papillae. Median antenna shorter than lateral antennae and palps. Antenniform papillae absent. Dorsal macrotubercles sessile, almost spherical, arranged in two transverse rows per segment, with six and seven macrotubercles each in midbody segments. Additional five papillae, hemispherical and small, per segment. Ventrum with six papillae per segment, hemispherical and small, arranged in a V-shape. Parapodia with one papilla on anterior surface. Acicular lobe from chaetiger 2, digitiform. Ventral cirri digitiform reaching acicular lobe tip. Compound chaetae (3–6) with short blades (up to five times as long as wide); all similar. Females with large tubercle near ventral edge of parapodia of chaetiger 6 and inflated ventral cirri of chaetigers 4–7. Males with a pair of oval tubercles, ventral cirri of chaetiger 6 also basally inflated.

##### Remarks.

The recent description of this species is complete and re-examination of material, even under the SEM did not provide further information about morphological features. Range of variation among chaetae of different specimens and segments show moderate variation and all studied blades range between 3–5 times as long as wide, are unidentate, and finely serrated (Fig. 13G–I). *Geminofilumgarciaalvarezi*&nbsp;comb. n. is similar to *G.bisphaeroserialis* (Hartmann-Schröder, 1974), comb. n. (South Africa), *G.arctowskyensis* (Hartmann-Schröder & Rosenfeldt, 1988), comb. n. (Antarctica), and *G.halldori* (Moreira & Parapar, 2012), comb. n. (Iceland) but they can be distinguished according to the number and arrangement of ventral papillae (Moreira et al. 2004, Moreira and Parapar 2012).

##### Distribution.

NW Iberian Peninsula (Moreira et al. 2004, Cacabelos et al. 2008).

##### Habitat.

From gravel to muddy sand and sandy mud and seagrass (*Zosteramarina* L.), 7–28 m depth (Moreira et al. 2004, Cacabelos et al. 2008).

#### Sphaerephesia

Taxon classificationAnimaliaPhyllodocidaSphaerodoridae

Fauchald, 1972, emended

Sphaerodoropsis Hartman & Fauchald, 1971: 69 (in part); Fauchald 1974: 261 (in part); Borowski 1994: 23 (in part); Moreira 2012: 30 (in part); Capa et al. 2014: 18 (in part).
Sphaerephesia Fauchald, 1972: 197 (in part); Fauchald 1974: 271 (in part); Capa and Bakken 2015: 238 (in part), Capa et al. 2016b: 14 (in part).

##### Type species.

*Sphaerephesialongisetis* Fauchald, 1972.

##### Diagnosis.

Body generally short and ellipsoid, some species slender. Head with short appendages, with or without spurs or basal papillae; antenniform papillae absent or present. Four longitudinal rows of dorsal macrotubercles, one transverse row per segment. Macrotubercles sessile, spherical or hemispherical, pear-shaped or with terminal papilla. Microtubercles (small tubercles with a collar and terminal papillae) absent. Additional papillae over body surface and parapodia. Parapodia with cylindrical or pear-shaped ventral cirri, not surpassing the tip of acicular lobe. Stout hooks in anterior chaetigers absent. All chaetae compound.

##### Remarks.

*Sphaerephesia* has been, up to know, diagnosed by the presence of terminal papillae on dorsal macrotubercles (e.g., Fauchald 1972, 1974, Capa et al. 2014). These papillae are not discrete nor separated by a constriction. Instead, they appear to be as a continuous thinner tip of the tubercles, in most cases. The most conspicuous example of well-developed terminal papillae is the recently described *Sphaerephesiaamphorata* Capa, Osborn & Bakken, 2016. There were nine species considered within this genus (Capa et al. 2016b). Some members of *Sphaerodoropsis* (prior to this study) present pear-shaped macrotubercles on some dorsal tubercles, or even with subtle terminal papillae. It was previously indicated that the validity of both genera and their boundaries are unclear and require assessment (Capa and Bakken 2015, Capa et al. 2016b). Analyses of molecular data in this study recover *Sphaerephesia* and members of *Sphaerodoropsis* Group 1 (Borowski 1994) both paraphyletic and together forming Clade 1 (Fig. 1). We are proposing the transfer of members of *Sphaerodoropsis* group 1 to *Sphaerephesia* and expanding the diagnosis of the latter to incorporate those sphaerodorids with four longitudinal rows of sessile macrotubercles, regardless their shape (hemispherical, spherical, pear-shaped or with terminal papilla). Members of *Sphaerephesia* were described lacking antenniform papillae and with four longitudinal rows of dorsal macrotubercles. However, there are congeners presenting antenniform papillae. *Sphaerephesiagesae* Moreira & Parapar, 2011, provided with eight longitudinal rows of macrotubercles, needs validation and it is for the time being considered with an uncertain position.

The species included in the genus after this study are:

*Sphaerephesiaamphorata* Capa, Osborn & Bakken, 2016

Type locality: North Carolina, USA, 640 m.

*Sphaerephesiaanae* (Aguado & Rouse, 2006), comb. n.

Type locality: Pacific Antarctic Ridge, 2216–2334 m.

*Sphaerephesiaartabrensis* (Moreira & Parapar, 2007), comb. n.

Type locality: Artabro Gulf, NW Iberian Peninsula, 209 m.

*Sphaerephesiabiserialis* (Berkeley & Berkeley, 1944), comb. n.

Type locality: Dease Strait, northern Canadian Arctic, 82 m.

*Sphaerephesiachilensis* Fauchald, 1974

Type locality: Seno and Estero de Reloncaví, Chile, intertidal to 80 m.

*Sphaerephesiacorrugata* (Hartman & Fauchald, 1971), comb. n.

Type locality: Off New England, USA, 400–1500 m.

*Sphaerephesiadiscolis* (Borowski, 1994), comb. n.

Type locality: Peru Basin, 4152 m.

*Sphaerephesiaelegans* (Hartman & Fauchald, 1971), comb. n.

Type locality: Off Brazil, 3730–3783 m.

*Sphaerephesiaexmouthensis* (Hartmann-Schröder, 1981), comb. n. Type locality: Exmouth, Western Australia, ? intertidal.

*Sphaerephesiafauchaldi* Kudenov, 1987

Type locality: Florida, Gulf of Mexico, 54 m.

*Sphaerephesiafurca* (Fauchald, 1974), comb. n.

Type locality: Chile-Peru Trench, Peru, 1296–1317 m.

*Sphaerephesiagesae* Moreira & Parapar, 2011.

Type locality: Bellingshausen Sea, Antarctica, 612–620 m.

*Sphaerephesiahutchingsae* Capa & Bakken, 2015

Type locality: East of Malabar, Sydney, Australia, 82 m.

*Sphaerephesiakitazatoi* (Shimabukuro et al., 2017), comb. n.

Type locality: São Paulo Ridge, South Atlantic, 4204 m.

*Sphaerephesialaevis* (Fauchald, 1974), comb. n.

Type locality: Chile-Peru Trench, Peru, 1296–1317 m.

*Sphaerephesialaureci* (Desbruyères, 1980), comb. n.

Type locality: Terrasse de Meriadzek, Bay of Biscay, 2325 m.

? *Sphaerephesialongesetosa* (Averincev, 1972), comb. n. (incertae sedis)

Type locality: Antarctica, 1000 m.

*Sphaerephesialongipalpa* (Hartman & Fauchald, 1971), comb. n.

Type locality: Off Bermuda, NW Atlantic, 1700 m.

*Sphaerephesialongipapillata* (Desbruyères, 1980), comb. n.

Type locality: Bay of Biscay, 4150 m.

*Sphaerephesialongiparapodium* (Katzmann, 1973), comb. n.

Type locality: Adriatic Sea, 20–60 m.

*Sphaerephesialongisetis* Fauchald, 1972

Type locality: Baja California, 957 m.

*Sphaerephesiamalayana* (Augener, 1933), comb. n.

Type locality: Banda, Indonesia, unknown depth.

*Sphaerephesiamamalaensis* Magalhães, Bailey-Brock & Barrett, 2011

Type locality: Oahu Island, Hawaii, 68 m.

*Sphaerephesiamartinae* (Desbruyères, 1980), comb. n.

Type locality: Banc Le Danois, Bay of Biscay, 1913 m.

*Sphaerephesiaparva* (Ehlers, 1913), comb. n.

Type locality: Eastern Antarctica, 380–3423 m.

*Sphaerephesiaphilippi* (Fauvel, 1911), comb. n.

Type locality: Kara Sea, Artic Ocean, 220 m.

*Sphaerephesiaprotuberanca* (Böggemann, 2009), comb. n.

Type locality: Guinea Basin, South Atlantic, 5048–5443 m.

*Sphaerephesiaregularis* Böggemann, 2009

Type locality: Guinea and Angola basins, South Atlantic, 5048–5051 m.

*Sphaerephesiasibuetae* (Desbruyères, 1980), comb. n.

Type locality: Banc Le Danois, Bay of Biscay, 1913 m.

*Sphaerephesiasimilisetis* Fauchald, 1972.

Type locality: Baja California, 461 m.

*Sphaerephesiastellifer* (Aguirrerezabalaga & Ceberio, 2005), comb. n.

Type locality: Capbreton Canyon, Bay of Biscay, 990–1040 m.

? *Sphaerephesiasimplex* (Amoureux, Rulllier & Fishelson, 1978), comb. n.

Type locality: Gulf of Suez, 30 m.

*Sphaerephesiatriplicata* (Fauchald, 1974), comb. n.

Type locality: off Durban, South Africa, 715–675 m.

*Sphaerephesiavittori* (Kudenov, 1987), comb. n.

Type locality: Gulf of Mexico, USA, 37–121 m.

*Sphaerephesiawilsoni* (Capa & Bakken, 2015), comb. n.

Type locality: Jervis Bay, Australia, 1–40 m.

#### Sphaerephesia artabrensis

Taxon classificationAnimaliaPhyllodocidaSphaerodoridae

(Moreira & Parapar, 2007) comb. n.

**Figure 14.**
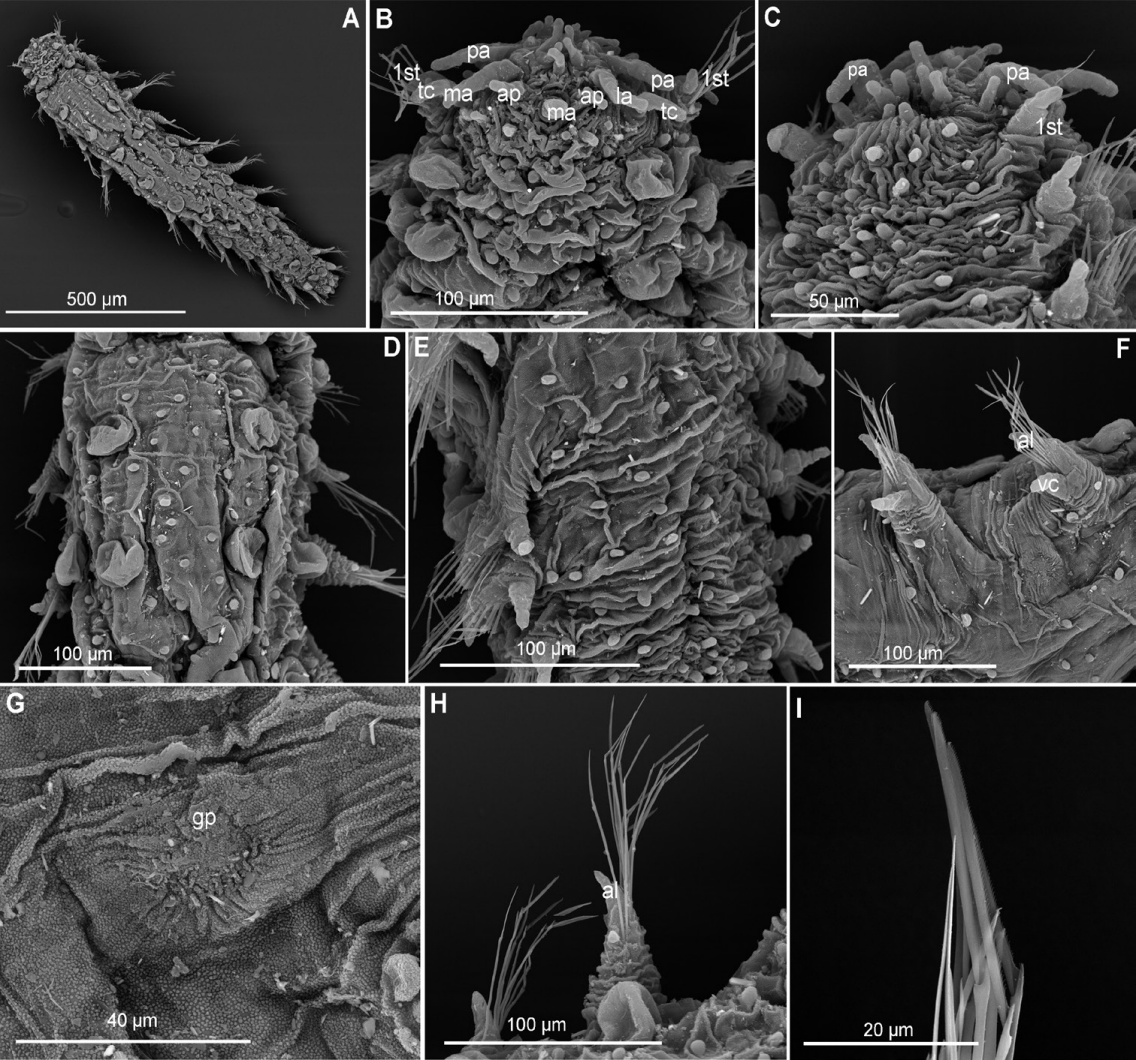
*Sphaerephesiaartabrensis* comb. n., scanning electron micrographs (MNCN 16.01/18461). **A** Complete specimen, dorsal view **B** head and chaetiger 1, dorsal view **C** anterior end, ventral view **D**&nbsp;chaetigers 4 and 5, dorsal view **E** anterior chaetigers, ventral view **F** parapodia, chaetigers 7 and 8, ventral view (female) **G** genital opening, between chaetigers 7 and 8, detail (female) **H** parapodium, chaetiger 4, dorsal view **I** chaetal fascicle, mid-body chaetiger.

**Figure 15.**
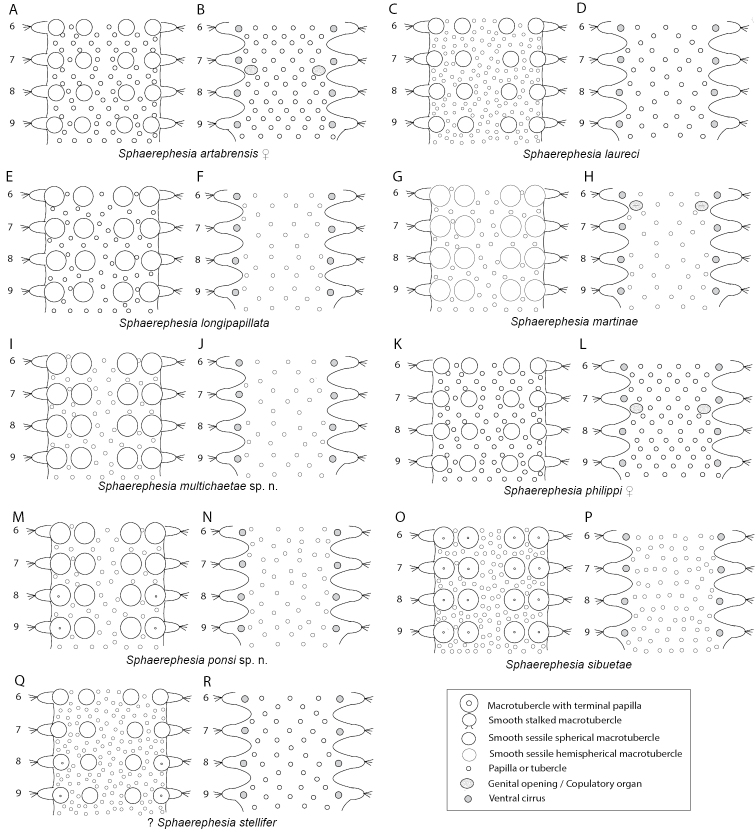
Stylized drawings of selected dorsal and ventral segments of species of *Sphaerephesia*, showing number and arrangement of epithelial tubercles and papillae. Epithelial papillae in Fig. 15E and F may not be accurate and the dorsal and ventral surfaces are covered by a thin layer of sediment.

Figs 5I , 14 , 15A, B

Sphaerodoropsis artabrensis Moreira & Parapar, 2007: 374–377, figs 1–2, 3A; Moreira et al. 2011: 30.

##### Type locality.

Ártabro Gulf, NW Iberian Peninsula, 43°40.192'N, 8°43.760'W, 209 m.

##### Material examined.

**Paratypes**: (3 specs) SMF 16881/3, Golfo Ártabro, NW Spain, DIVA-Artabria I-02, sample EBS-250, 43°41.11'N, 08°44.23'W, 257 m, 14 Sep 2002.**Additional material** (119 specs): **NW Spain**: MNCN 16.01/13270 (40 specs), Golfo Ártabro, 43°40.25'N, 08°43.75'W, 197–207 m, 12 Sep 2003. MNCN 16.01/18461 (79 specs), 42°30.39'N, 09°19.52'W, 147 m, 17 Sep 2004.

##### Diagnosis.

Body short and ellipsoid, up to 1.75 mm long. Palps and antennae smooth, lacking spurs or basal papillae. Median antenna shorter than palps and lateral antennae. Antenniform papillae present. Four longitudinal rows of macrotubercles in a single transverse row per segment. Macrotubercles sessile, small, spherical to pear shaped. Additional small spherical papillae on dorsum (arranged in four irregular transverse rows with ca. 20 papillae per segment) and ventral surfaces. Parapodia conical, with 3–4 sub-equal papillae (1–2 ventral, one anterior, one dorsal). Acicular lobe from chaetiger 2. Ventral cirri digitiform as long as acicular lobe tip, or shorter. Compound chaetae with long blades (8–20 times as long as wide), unidentate and with serrated edge. Some live and fixed specimens have pigmented orange to brown macrotubercles. Some females with oocytes, without visible nucleus; genital pores observed between chaetiger 7 and 8.

##### Remarks.

*Sphaerodoropsisartabrensis* was described based on the unique combination of the following morphological features: spherical to pear-shaped macrotubercles arranged in four longitudinal rows, 3–4 sub-equal parapodial papillae, chaetae with long blades (8–20 times as long as wide), showing gradation within each fascicle. The original description of this species is complete and re-examination of Iberian material, even under the SEM, did not provide additional information about morphological features, but allowed to verify some of the attributes. Palps and antennae are smooth, lacking spurs or basal papillae (Fig. 14B, C); median antenna shortest (Fig. 14B). Antenniform papillae present (Fig. 14B). Small spherical papillae over dorsum forming four irregular transverse rows with about 20 papillae per segment in addition to the four macrotubercles (Fig. 15A), similar number and arrangement on ventrum (Fig. 15B). Parapodia conical, with 3–4 sub-equal papillae (1–2 ventral, one anterior, one dorsal) (Figs 5I, 14E, F, H). Acicular lobe from chaetiger 1–2. Ventral cirri digitiform as long as acicular lobe tip, or shorter (Fig. 14F). Compound chaetae with long blades (8–20 times as long as wide), unidentate and with serrated edge (Fig. 14F, H–J). It is the first time that genital pores are reported in the species. These have only been observed in females, as a flat, porous area, between parapodia of chaetiger 7 and 8 (Fig. 14G).

This species is herein transferred to the genus *Sphaerephesia* due to the number and arrangement of dorsal macrotubercles in four longitudinal rows.

Specimens of *S.artabrensis* comb. n., resemble those assigned herein as *Sphaerephesiaphilippi* comb. n. from Nordic waters but they present subtle but consistent differences. Northern specimens are generally larger (Iberian specimens are up to 1.75 mm long and northern ones almost double in size), and bear a few more papillae in the prostomium, dorsum and parapodia (6–7 instead of the 3–4 in Iberian specimens). Specimens from northern localities present an acicular lobe from segment 1, instead of segment 2. Ventral cirri do not surpass the acicular lobe. It would be most interesting to confirm that these differences can be attributed to different lineages and not to the intraspecific range of variation of a species with a broad distribution from Spain to the Kara Sea. However, we have been unable to find specimens collected all along the coasts, and instead only in NW Spain and then from Skagerrak to the North. Moreover, extraction and amplification of DNA in specimens collected in the NW of Spain was unsuccessful and were therefore not included in analyses.

##### Distribution.

NW Iberian Peninsula (Moreira and Parapar 2007, Moreira et al. 2011).

##### Habitat.

Continental slope, in sandy-muddy sediments, 200–2200 m (Moreira and Parapar 2007, Moreira et al. 2011).

#### Sphaerephesia laureci

Taxon classificationAnimaliaPhyllodocidaSphaerodoridae

(Desbruyères, 1980) comb. n.

**Figure 16.**
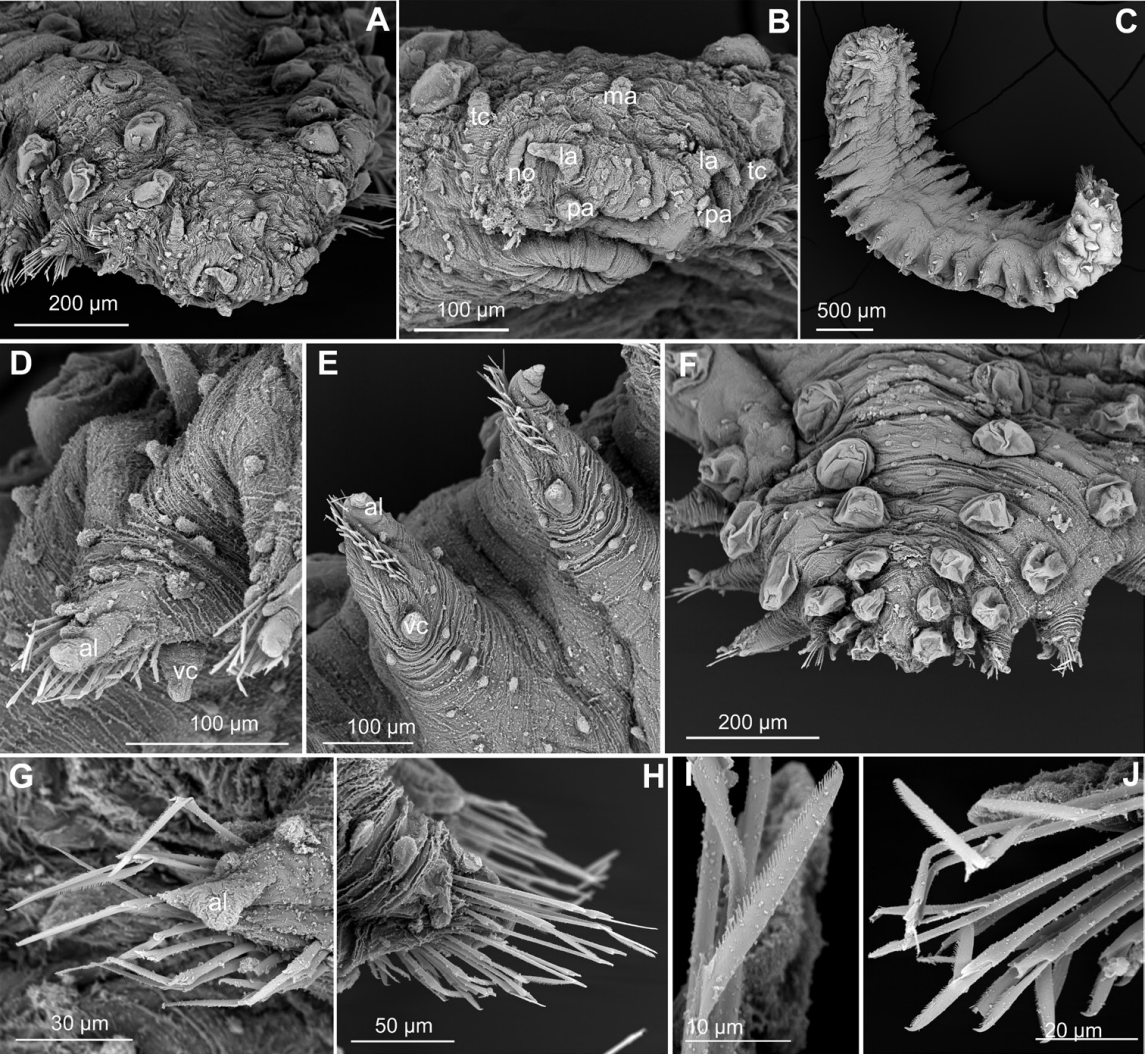
Sphaerephesiacf.laureci comb. n., scanning electron micrographs (NTNU-VM 73789). **A**&nbsp;Anterior end, frontal view **B** detail of head, frontal view **C** complete specimen, ventral view **D** anterior parapodium, anterior view **E** midbody parapodia ventral view **F** posterior end, dorsal view **G** chaetal bundle, first chaetiger **H** chaetae, midbody chaetiger **I** detail of chaeta **J** chaetae posterior chaetiger.

Figs 5J, K , 15C, D , 16

Sphaerodoropsis laureci Desbruyères, 1980: 219, pl. 5A–C.

##### Type locality.

Meriadzek, Terrace, Bay of Biscay, 47°31'N, 9°35'W, 2325 m.

##### Material examined.

**Holotype**: MNHN TYPE 1286, Meriadzek, Terrace, Bay of Biscay, 47°31'N, 9°35'W, 2325 m.

##### Additional material.

(2 specs) **Barents Sea**: NTNU-VM 73789, (2 specs, one on SEM stub), Hopenbanken, 75°21.940'N, 26°37.120'E, 191 m, 29 Apr 2008.

##### Diagnosis.

Body cylindrical, with blunt anterior end, up to 4 mm long. Head appendages smooth, without spurs, median antenna shorter than other appendages. Antenniform papillae present. Dorsum with four longitudinal rows of macrotubercles in a single transverse row per segment, from segment 2. Macrotubercles sessile, spherical in anterior and pear-shaped in posterior segments. Additional small hemispherical papillae on dorsum, in four irregular transverse rows per segment, each segment with ca. 30 papillae. Ventrum with four slightly irregularly arranged transverse rows of papillae per segment, each segment with ca. 30–40 papillae. Parapodia digitiform from chaetiger 3, with ca. 12–14 rounded sub-equal papillae. Acicular lobe from segment 2. Ventral cirri digitiform surpassing acicular lobe tip. Compound chaetae with medium length blades (6–8 times as long as wide), showing slight gradation within fascicles.

##### Variation.

For this species only the holotype is known. However, some specimens from Norway are herein considered as potentially belonging to the same species. There are, however, some differences between the holotype and the Norwegian specimens. Norwegian specimens lack antenniform papillae (Fig. 16A, B). Dorsal papillae (additional to the macrotubercles) are arranged in four transverse rows per segment, each with 14–18 papillae on mid-body segments (near half of those in the original description, Fig. 15C). Ventral papillae also about half as many as in the holotype (Figs 15D, 16C). Interestingly, the parapodia bear ca. 16–18 rounded sub-equal papillae (Figs 5J, 16D, E) instead of the 14 reported in the original description. The acicular lobe is present from segment 1 (instead of 2). Ventral cirri are digitiform and do not surpass the acicular lobe tip (Fig. 16D, E). Otherwise, the general aspect, number and relative length of prostomial appendages and chaetal morphology, is similar between specimens examined.

##### Remarks.

Re-examination of the holotype resulted in a different interpretation of some morphological attributes with respect of the original description. We now state the prostomial appendages to be small and simple, instead of the bifurcated median antenna described originally. The dorsal macrotubercles are not invaginated and neither dorsoventrally flattened anymore in the preserved specimen. The number and arrangement of papillae is not clear from the original description. Approximately 12–14 spherical papillae randomly distributed over parapodial surface have been counted after re-examination (Fig. 5J).

*Sphaerephesialaureci* comb. n. is distinguished from other species in the genus by a unique combination of features: head appendages smooth, without papillae or spurs, dorsal macrotubercles spherical to pear-shaped, ca. 30 additional papillae, arranged in four irregular transverse rows both in dorsum and ventrum, 12–14 parapodial papillae and compound chaetae with blades up to eight times as long as wide.

##### Distribution.

Bay of Biscay and the western Barents Sea (Desbruyères, 1980, present study).

##### Habitat.

No details were provided in the original description.

#### Sphaerephesia longipapillata

Taxon classificationAnimaliaPhyllodocidaSphaerodoridae

(Desbruyères, 1980) comb. n.

Figs 5L , 15E, F

##### Type locality.

Bay of Biscay, 47°31'N, 9°35'W, 4150 m.

##### Material examined.

**Holotype**: MNHN TYPE 1283, Bay of Biscay, 47°31'N, 9°35'W, 4150 m.

##### Diagnosis.

Body elongated, almost rounded in cross section, with blunt anterior end. Head appendages smooth, without spurs, median antenna shorter than other appendages. Antenniform papillae not conspicuous. Dorsum with four longitudinal rows macrotubercles in a single transverse row per segment, from segment 2. Macrotubercles large, sessile, spherical. Additional small spherical papillae on dorsum with unclear arrangement due to sediment covering epithelium. Ventrum with small hemispherical papillae. Parapodia digitiform from chaetiger 3, with 7–8 elongated papillae, larger papilla in dorso-distal position. Acicular lobe from segment 2. Ventral cirri digitiform surpassing acicular lobe tip. Approximately 20–25 compound chaetae with long blades (ca. 8–12 times as long as wide), showing slight gradation within fascicles.

##### Remarks.

The holotype has large, turgid, and almost spherical dorsal macrotubercles, but it is covered by a thin layer of sediment that makes the assessment of the number and arrangement of the small epithelial papillae over the dorsal and ventral body surface difficult (therefore Fig. 15E and F not necessarily accurate). A feature not highlighted in the original description is the number of chaetae, that can reach up to 30 in some mid-body fascicles, all with relatively long blades, measuring 8–12 times as long as wide. *Sphaerephesialongipapillata* comb. n. is distinguished from other congeners in the combination of three features: elongated and almost cylindrical body, presence of large (occupying most of the dorsum surface) and spherical macrotubercles, not pear-shaped (Fig. 15E) and chaetae with medium-long blades (ca.ten times longer than wide). The parapodia and parapodial papillae in the holotype are stretched, but it could be due to muscular relaxation of this particular specimen. The sigmoid acicula could not be verified in the opaque specimen. Two other NE Atlantic *Sphaerephesia* species with similar body shape and chaetae are *S.philippi* comb. n. and *S.artabrensis* comb. n., but both present pear-shaped macrotubercles in at least posterior chaetigers. Moreover, *S.artabrensis* comb. n. has less parapodial papillae (3–4 on each parapodium). *Sphaerephesialongipapillata* comb. n. was also characterised by the length of a dorso-distal parapodial papilla, a feature that could vary after fixation (Helm and Capa 2015). However, the dorso-distal papilla is longer than the others in the material examined, and could be a distinct diagnostic feature together with the high number of chaetae per fascicle.

##### Distribution.

Only known from type locality.

##### Habitat.

No details were provided in the original description.

#### Sphaerephesia martinae

Taxon classificationAnimaliaPhyllodocidaSphaerodoridae

(Desbruyères, 1980) comb. n.

**Figure 17.**
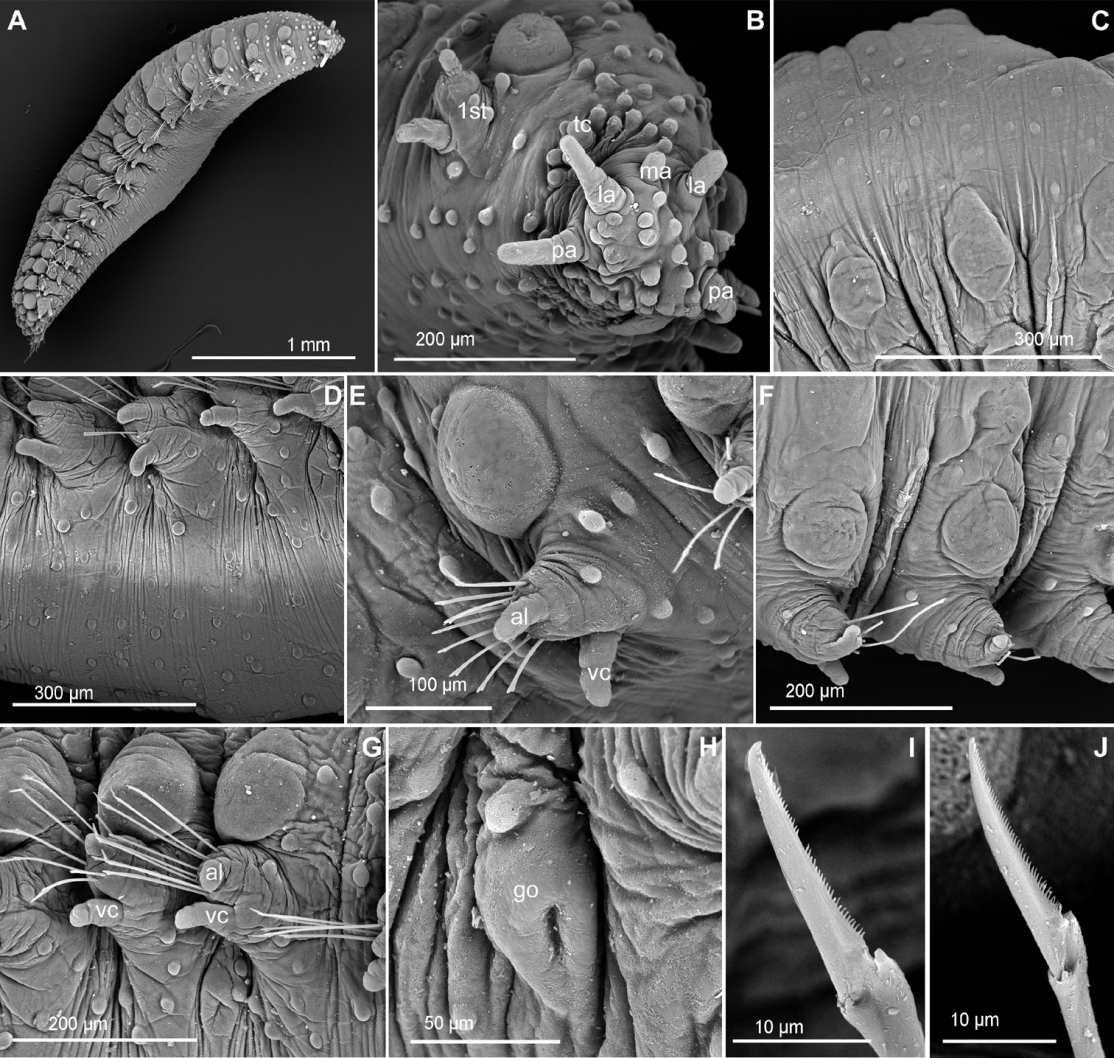
*Sphaerephesiamartinae* comb. n., scanning electron micrographs (SMFDZMB HH 31236 and SMF 25286). **A** Complete specimen, lateral view **B** head, frontal view **C** dorsal epithelial tubercles, mid-chaetigers **D** mid-body chaetigers, ventral view **E** parapodium and macrotubercle third chaetiger, anterior view **F** chaetiger mid-body segment, dorsal view **G** chaetiger mid-body segment, ventral view **H**&nbsp;genital opening **I, J** chaetae mid-body segments.

Figs 5M , 15G, H , 17

##### Type locality.

Banc Le Danois, Bay of Biscay, 44°05.2'N, 4°19.4'W, 1913 m.

##### Material examined.

**Holotype**: Banc Le Danois, Bay of Biscay, 44°05.2'N, 4°19.4'W, 1913 m.

##### Additional material.

(18 specs) **Argentinian Basin**: SMFDZMB HH 21466 (2 specs, 1 spec. used for DNA sequencing, SPH021), 036°00.61'S, 049°01.54'W, 4607 m, 16 Jul 2009; **Iceland**: SMF 25283 (1 spec.), South Iceland, Iceland Basin, 60°02.73'N, 021°28.06'W, 2749 m, 28 Aug 2011; SMF 25286 (8 specs, 2 on SEM stub), South Iceland, 60°02.73'N, 4 021°28.06'W, 2749 m, 28 Aug 2011; SMF 23911 (1 spec., DNA SPH063.), South Iceland, 60°02.73'N, 021°28.06'W, 2749 m, 28 Aug 2011; SMF 23910 (1 spec. used for DNA sequencing, SPH048), Iceland, 036°00.61'S, 049°01.54'W, 2749 m, 7 Sep 2011; SMFDZMB HH 31236 (1 spec. on SEM stub), South Iceland, Iceland Basin, 60°21.48'N, 018°08.24'W, 2567 m, 30 Aug 2011; SMF 25285 (2 specs) South Iceland, Iceland Basin, 62°33.10'N, 020°23.71'W, 1384 m, 2 Sep 2011. **Barents Sea**: NTNU-VM 68189 (1 spec. used for DNA sequencing, SPH 293), Finnmark, 72°18.588'N, 32°20.478'E, 313 m, 4 Aug 2013.

##### Diagnosis.

Body ellipsoid, with convex dorsum and slightly flattened dorsoventrally, up to 3 mm long. Head appendages smooth, without spurs, median antenna slightly shorter than other appendages. Antenniform papillae shorter than lateral antennae. Dorsum with four longitudinal rows of large, hemispherical sessile macrotubercles in a single transverse row per segment, from segment 2. Additional papillae on dorsum arranged in four irregular transverse rows. Ventrum with three transverse rows of papillae similar in shape and size to dorsal. Parapodia short and conical, with 2–3 small, rounded papillae: one on each anterior and posterior surfaces, one in the ventrum of some parapodia. Acicular lobe from segment 1. Ventral cirri digitiform reaching acicular lobe tip. Approx. 7–10 compound chaetae with medium length blades (ca. 5–6 times as long as wide); unidentate and with fine spinulation along its margin. Several paratypes were described as possessing orange macrotubercles (Desbruyères, 1980). One pair of genital structures between the base of parapodia 6 and 7.

##### Re-description of holotype.

*Measurements and general morphology.* Holotype 15 chaetigers, 2.9 mm long, 0.5 mm maximum width. Body almost cylindrical, slightly more rounded anteriorly and tapering posteriorly (Fig. 17A). Segmentation not distinct, pigmentation absent in preserved material examined.

*Head.* Prostomium fused to peristomium (Fig. 17B). Palps and lateral antennae digitiform, slightly wider at base, similar in shape and size. Median antenna digitiform, shorter than other appendages. Antenniform papillae present, slightly larger than other head papillae. Head with more than ten hemispherical papillae. Tentacular cirri digitiform, half of the length of lateral antennae (Fig. 17B).

*Tubercles.* Dorsal macrotubercles, hemispherical, wide and low, sessile and smooth, arranged in four rows, occupying most of dorsum; one transverse row per segment, except for the first segment where only two are present (Figs 15G, 17A). Dorsal papillae low and rounded, distributed in four transverse rows approximately, more numerous and conspicuous in anterior segments (Fig. 17C). Ventral papillae similar in shape and size to dorsal, arranged in three transverse rows per segment (Figs 15H, 17D).

*Parapodia.* Parapodia short and wrinkled, twice as long as wide, conical in shape. Ventral cirri conical, shorter than the width of parapodia. Acicular lobe, shorter than ventral cirri, present from first chaetiger (Fig. 17A). Two or three small, rounded papillae: one on each anterior and posterior surfaces, one in the ventrum of some parapodia (Figs 5M, 17E–G).

*Chaetae.* Seven to ten chaetae per parapodia. All chaetae compound medium in length (5–6 times as long as wide), similar within and between parapodia, unidentate and with fine serrated edge (Fig. 17E, G, I, J).

*Pygidium.* Two globular cirri, similar to dorsal macrotubercles and two ventral small cirri (perhaps the ventral cirri of last segment). Median papilla not observed.

*Internal features.* Pigmented nuchal organs or eyes not seen. Pharynx not observed.

*Reproductive features.* Sexual structures, genital openings or gametes not observed in holotype, paratypes or additional material.

##### Variation.

Studied specimens measured 2–3 mm long. In all specimens macrotubercles are hemispherical. Some variation regarding the number of parapodial papillae has been observed. Most specimens present one hemispherical papilla on the anterior surface and a similar one on the posterior side, but a smaller ventral papilla may be also present. One pair of genital openings between the base of parapodia 6 and 7 in one specimen (SMFDZMB HH 31236, Fig. 17H).

##### Remarks.

The shape of macrotubercles is remarkable: hemispherical, low and wide, with a rounded and smooth surface and no papillae. This feature, together with the presence of almost inconspicuous additional epithelial papillae is one of its main diagnostic features (Desbruyères, 1980), that distinguishes this species from other congeners.

The invaginations described behind the lateral antennae in the original description, are here considered as the openings of the nuchal organs. The parapodial papillae were not described in the original description, and at least one hemispherical papilla was observed in the anterior parapodial surface, from chaetiger 6 in the holotype, and additional material present up to three parapodial papillae. Holotype is full of gametes but sexual structures or genital openings were not detected. Sexual structures are described in this species for the first time in additional material from Iceland.

##### Distribution.

The species is here newly reported for Iceland and the Barents Sea. It had previously been reported in Bay of Biscay (Desbruyères, 1980).

##### Habitat.

Sediments from 30 to 2750 m (Desbruyères, 1980, present study).

#### Sphaerephesia multichaeta

Taxon classificationAnimaliaPhyllodocidaSphaerodoridae

Capa, Moreira & Parapar sp. n.

http://zoobank.org/416D3A0B-25AE-48CC-B68B-0A2B917142F4

**Figure 18.**
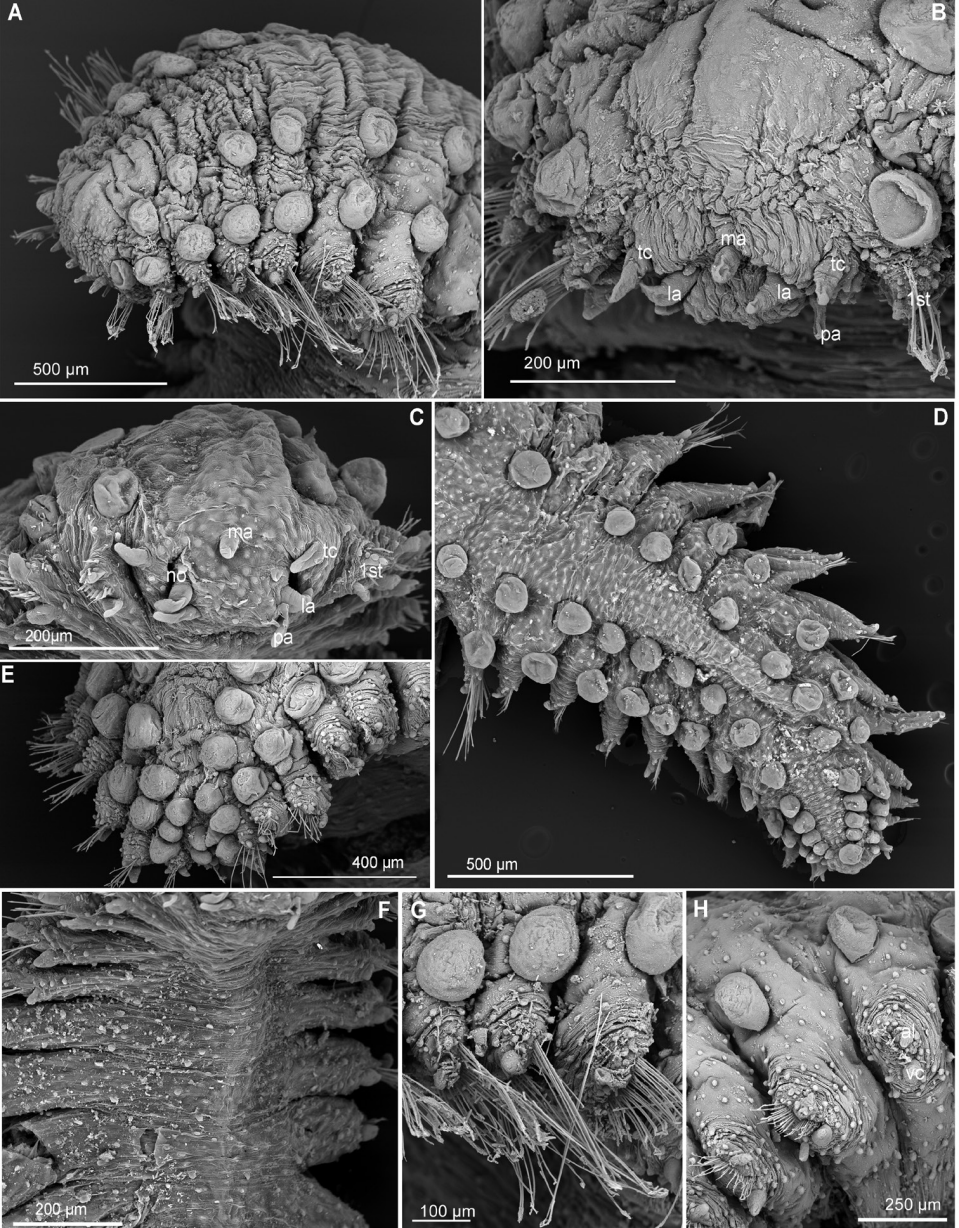
*Sphaerephesiamultichaeta* sp. n. (NTNU-VM24809, ZMH P13351), scanning electron micrographs. **A** Anterior end, side view (NTNU-VM 24809) **B** head, frontal view (NTNU-VM 24809) **C** head, antero-ventral view (ZMH P13351) **D** posterior end, dorsal view (ZMH P13351) **E** posterior end, dorsal view (NTNU-VM 24809) **F** ventrum, showing arrangement of ventral papillae (ZMH P13351) **G** Chaetigers 4–6, antero-dorsal view (NTNU-VM 24809) **H** mid-body parapodia, lateral view (NTNU-VM 24809).

**Figure 19.**
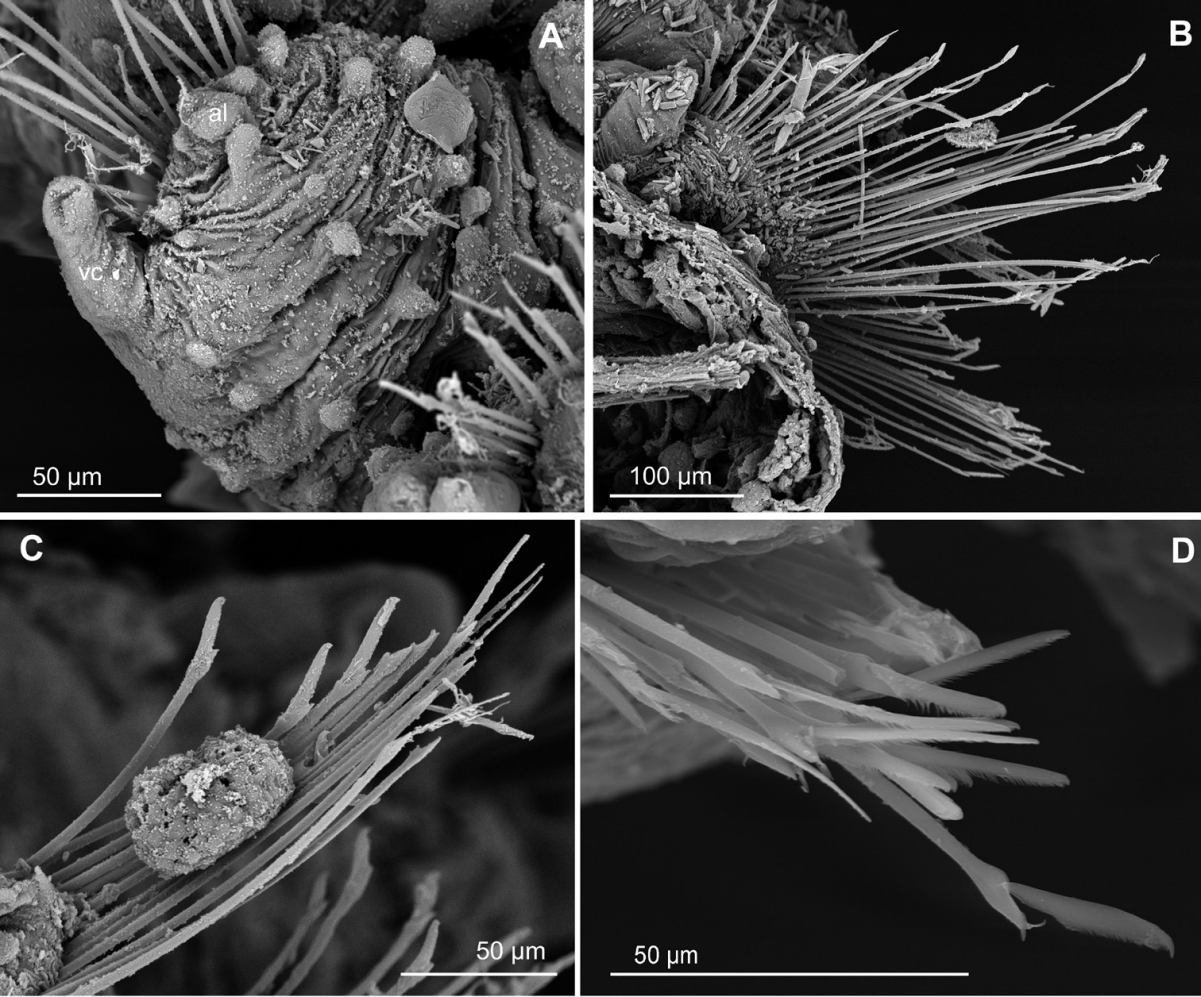
*Sphaerephesiamultichaeta* sp. n. (NTNU-VM 24809, NTNU-VM 24810), scanning electron micrographs. **A** posterior parapodia, anterior view (VM 24809) **B** complete chaetal fascicle, mid-body chaetiger (VM 24809) **C** chaetiger 1, anterior view (NTNU-VM 24809) **D** chaetae, mid-body chaetiger (NTNU-VM 24810).

Figs 5N , 15I, J , 18 , 19

##### Type locality.

Borgenfjorden, Trondheimsfjord, 25 m.

##### Material examined.

**Holotype**: NTNU VM 24856, Norway, Trondheimsfjord, Borgenfjorden, 63°53'N, 11°20'E, 25 m, 14 July 1970. **Paratypes** (10 specs): **Norwegian Sea**, Trondheimsfjord, Borgenfjorden NTNU-VM 24809, 63°53'N11°20'E, 10 m, 04 May 1971 (1 spec. on SEM stub); NTNU-VM 24810 (2 specs, 1 on SEM stub), 10 m, 11 Aug 1970; NTNU-VM 24852 (1 spec.), 10 m, 02 Jun 1971; NTNU-VM 24854 (1 spec.), 20 m, 11 Aug 1970; NTNU-VM 24851 (1 spec.), 10 m, 13 May 1970; NTNU-VM 24853 (1 spec.), 15 m, 06 Oct 1970; NTNU-VM 24855 (1 spec.), 20 m, 06 Oct 1970; NTNU-VM 24857 (1 spec.), 25 m, 16 Jun 1970; NTNU-VM 24858 (1 spec.), 25 m, 09 Feb 1971; NTNU-VM 24859 (1 spec.), 25 m, 02 June1971.

##### Additional material.

(1 spec.) **Skagerrak**ZMH P13351, 58°07'N, 10°34'E, 196m, (1 spec. on SEM stub).

##### Diagnosis.

Body ellipsoid, with convex dorsum and slightly flattened dorsoventrally, up to 1 mm long. Palps and antennae smooth, lacking spurs. Four longitudinal rows of dorsal macrotubercles, in a single transverse row per segment. Macrotubercles sessile, spherical to pear-shaped. Additional minute spherical papillae scattered on dorsum (approx. seven transverse rows with ca. 100 low papillae per segment), often inconspicuous. Ventrum with even smaller papillae in four transverse rows per segment, often inconspicuous. Parapodia with 20–40 spherical papillae. Acicular lobe from segment 1, small and rounded. Compound chaetae, numerous (up to 40 per fascicle), with blades slightly decreasing in length dorso-ventrally (3–8 times their width), unidentate, with finely serrated edge.

##### Description.

*Measurements and general morphology.* Holotype with ellipsoid body, 0.7 mm long, 0.1 mm wide and with 27 segments; with blunt ends, with a convex dorsum and flattened ventrum. Segmentation not conspicuous. Pale, with brownish granules in some macrotubercles, in fixed material.

*Head.* Head fused to first chaetiger (Fig. 18A–C). Palps and lateral antennae conical, 2–3 times longer than wide, wrinkled, and lacking spurs or basal papilla (Fig. 18A–C). Median antenna conical, slightly shorter than lateral antennae (Fig. 18A–C). Antenniform papillae absent (Fig. 18A–C). Head papillae rounded, apparently randomly arranged. Tentacular cirri conical, similar to lateral antenna (Fig. 18A–C).

*Tubercles.* Medium-sized dorsal macrotubercles arranged in four longitudinal rows, one transverse row per segment, with exception of first chaetiger with only two macrotubercles (Fig. 18A–C). Macrotubercles sessile, spherical and smooth in anterior segments, and pear-shaped in posterior segments (Fig. 18A–E). Distance between dorsalmost rows is larger than these to lateralmost longitudinal rows. Additional dorsal papillae, low, rounded, arranged in approximately seven irregular transverse rows per segment along dorsal surface (Figs 15I, 18D), and in four transverse rows per segment in ventrum (Figs 15J, 18F). Papillae are not conspicuous in all segments; consequently epithelium seems smooth in some parts of the body.

*Parapodia.* Parapodia subtriangular, with wide dorso-ventral base as long as wide at mid-body segments (Figs 18D–H, 19A). Ventral cirri conical, small, not protruding from parapodia (Fig. 18H). Acicular lobe from chaetiger 1. Numerous (ca. 20 in mid-chaetigers) small and spherical papillae distributed randomly over parapodial surfaces (Fig. 5N).

*Chaetae.* All chaetae compound, with unidentate and finely serrated blades. First chaetiger with blades 7–9 times longer than wide. Midbody chaetigers with blades ranging 3–6 times longer than wide within each parapodia. Number of chaetae from 11–13 in first segment to 25–30 in mid-body segments (Figs 18G–H, 19B–D). One straight acicula per parapodia.

*Pygidium.* A pair of piriform anal cirri, similar to posterior macrotubercles and digitiform medio-ventral anal papilla (Fig. 18D, E).

*Internal features*. Pharynx slightly protruded though mouth in some specimens. Pharynx and internal organs not discernible.

*Reproductive features*. Sexual structures or genital openings not observed. Preserved specimens opaque and gametes not detected.

##### Variation.

Size range of material examined: 0.6–0.9 mm long, 0.08–0.15 mm wide, with 21–30 chaetigers. The holotype is somehow more inflated and elongated than the paratypes. These are more flattened dorso-ventrally and bodies seem to be more ellipsoid. This could be due to body collapse or perhaps the holotype is inflated from preservation. Head appendages are small and conical in all specimens examined; antenniform papillae not observed. Paratypes are homogenous in the general shape of the body, presence of large parapodia, small ventral cirri and presence of numerous, medium length blade falcigers. They differ in the number of epithelial papillae observed, in part probably due to the different conditions of the epithelium. We suspect the body and parapodia bear numerous small spherical papillae but these are only conspicuous in well-preserved specimens, otherwise they look almost smooth. Nuchal organs pits observed in several specimens (e.g., Fig. 18C). Sexual structures not seen.

##### Etymology.

The specific epithet, *multichaeta* (masculine), refers to the extraordinary number (*multi*) of bristles (*chaetae*, Greek origin) present in the fascicles of, at least, some specimens.

##### Remarks.

*Sphaerephesiamultichaeta* sp. n. belongs to the group of spherodororids with four longitudinal rows of dorsal and macrotubercles (i.e., *Sphaerephesia* after the present study). It is characterised by a unique combination of features: macrotubercles at least in posterior segments are pear-shaped, parapodia bear a great number of papillae (20–40) and chaetae (20–40). Only *Sphaerephesiasibuetae* and *S.similisetis* have been reported with 20–25 chaetae per fascicle (Fauchald 1972, Desbruyères 1980, Moreira et al. 2011). Likewise, also these two species have been reported with 20–30 parapodial papillae (Fauchald 1972, Desbruyères 1980, Moreira et al. 2011), but these numbers do not reach those accounted in the new species. *Sphaerephesiamultichaeta* sp. n. is distinguished from these two congeners in the chaetal morphology (with blades up to eight times as long as wide) while *S.sibuetae* and *S.similisetis* have longer blades (7–13 and 20–25 times as long as wide, respectively).

##### Distribution.

Most specimens were collected in the Trondheimsfjord, but also from Skagerrak.

##### Habitat.

Habitat soft bottom with mud, 10–25 m deep.

#### Sphaerephesia philippi

Taxon classificationAnimaliaPhyllodocidaSphaerodoridae

(Fauvel, 1911) comb. n.

**Figure 20.**
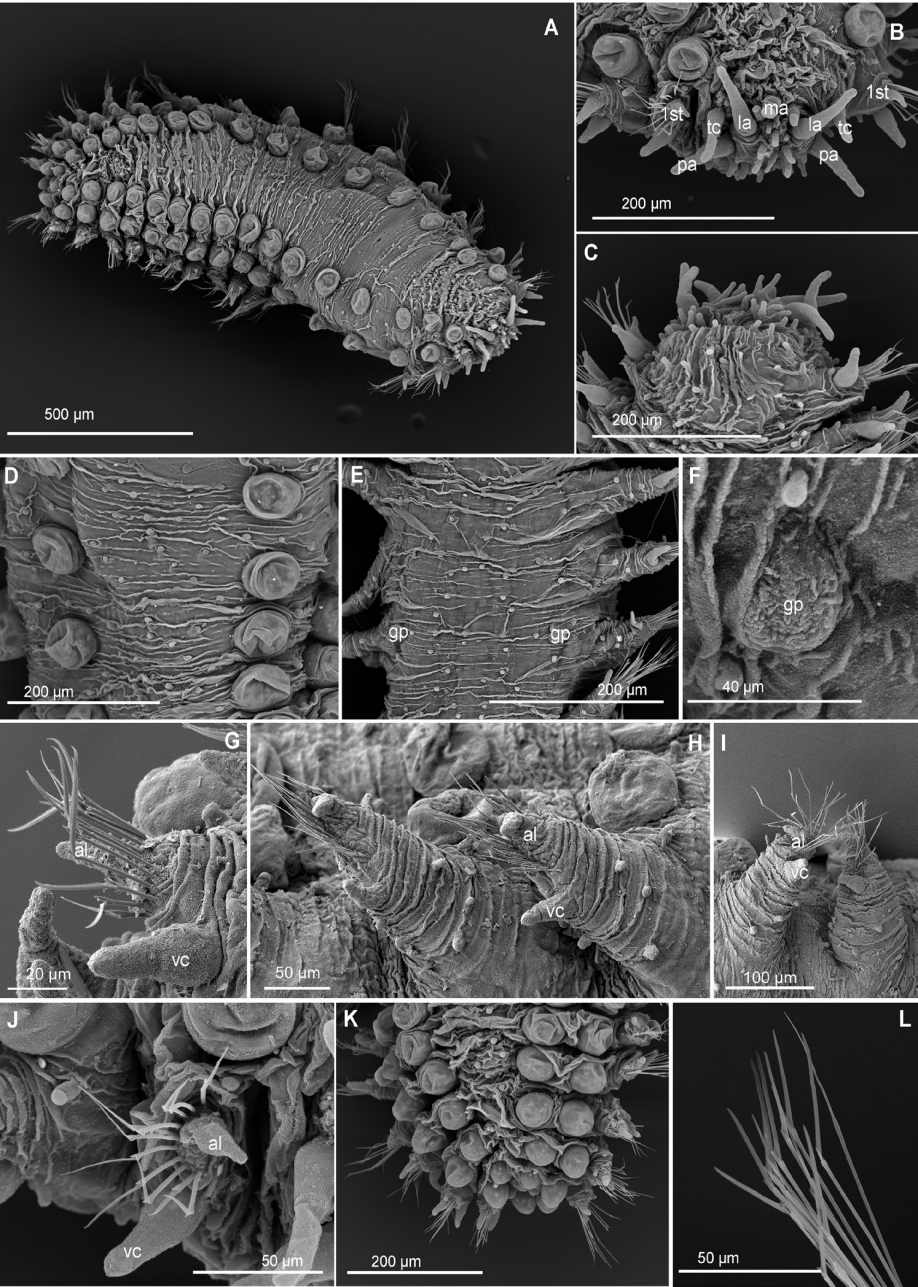
*Sphaerephesiaphilippi* (ZMBN 129496; **G–I**ZMBN 129497), scanning electron micrographs. **A** Complete specimen, dorsal view **B** head, frontal view **C** head, antero-ventral view **D** mid-body chaetigers, dorsal view **E** mid-body chaetigers, ventral view **F** genital pores, between parapodia of chaetiger 7 and 8 **G** parapodium, chaetiger 1, posterior view **H** mid-body parapodia, anterior view **I** mid-body parapodia, lateral view **J** chaetal fascicle, chaetiger 1 **K** posterior end, dorsal view **L** chaetae, mid-body chaetiger, detail.

Figs 5O , 8D , 15K, L , 20

Sphaerodorum philippi Fauvel, 1911: 19–21, fig. 16–20 (not S.philippi Hartmann-Schröder, 1971).

##### Type locality.

Kara Sea, Russia, 71°32'N, 57°10'E, 220–0 m.

##### Material examined.

(ca 1480 specs) **Greenland Sea**: ZMBN 127275 (~25 spec.), 68°53.5'N, 14°14.3'W, 1588 m, 15 Mar 1984; ZMBN 127287 (15 spec.), Jan Mayen, 70°48.6'N, 09°43.7'W, 886 m, 27 Jul 1986; ZMBN 127295 (4 spec.), 69°57.3'N, 18°08.9'W, 1618 m, 12 Jun 1987; NTNU-VM 32347 (2 spec.), Jan Mayen, 70°36.19'N, 9°20.72'W, 31 m, 17 Sep 1999; NTNU-VM 32348 (15 spec.), Jan Mayen, 71°06.41'N, 9°35.26'W, 514 m, 15 Sep 1999; NTNU-VM 32349 (35 spec.), Jan Mayen, 70°45.07'N, 7°57.74'W, 771 m, 16 Sep 1999; NTNU-VM 32350 (496 spec., 1 on SEM), Jan Mayen, 70°38.87'N, 9°22.33'W, 599 m, 17 Sep 1999; SMF 24843 (1 spec. for DNA sequencing, SPH052), East Greenland, Denmark Strait, 67°12.81'N, 026°14.50'W, 696.9 m, 14 Sep 2011; SMF 25287 (7 specs), East Greenland, Denmark strait, 67°50.79'N, 023°41.76'W, 1248 m, 15 Sep 2011; SMF 25288DZMB-HH 30452 (3 specs), East Greenland, Denmark Strait, 67°50.79'N, 023°41.76'W, 1248 m, 15 Sep 2011; SMF 25289 (4 specs), East Greenland, Denmark Strait, 67°50.790'N, 23°41.760'W, 1248 m, 15 Sep 2011; SMF 25290 (1 spec.), East Greenland, Denmark Strait 67°38.77'N, 026°44.78'W, 320 m, 14 Sep 2011; **Iceland**: SMF 25291 (6 specs), East Iceland, Norwegian Sea, 66°18.06'N, 012°22.38'W, 730,8m¸ 22 Sep 2011; SMF 25292 (1 specs), East Iceland, 66°18.06'N, 012°22.38'W, 730,8m¸ 22 Sep 2011; SMF 23903 (1 for DNA sequencing), North East Iceland, 69°6.66'N, 9°55.02'W, 2202 m , 17 Sep 2011; SMF 25293 (3 specs), North East Iceland, 69°6.66'N, 9°55.02'W, 2202 m , 17 Sep 2011; SMF 23903 (1 spec. For DNA sequencing, SPH061), North East Iceland, 69°6.66'N, 9°55.02'W, 2202 m , 17 Sep 2011; SMF 25294; (2 specs), South Iceland, Iceland Basin, 62°33.50'N, 020°21.18'W, 1392m, 2 Sep 2011; **Barents Sea**: ZMBN 127298 (5 spec.), Finnmark, 71°16.53'N, 27°0.94'E, 276 m, 16 Apr 2011; ZMBN 127305 (6 spec.), Finnmark, 71°11.415'N, 32°14.991'E, 226 m, 10 Aug 2013; ZMBN 127323 (10 spec.), Finnmark, 70°17.79'N, 31°18.83'E, 217 m, 18 Aug 2013; **Norwegian Sea**: ZMBN 127299 (10 spec.), Nordland, 67°48.276'N, 9°41.126'E, 823 m, 22 Sep 2011; ZMBN 127302 (18 spec.), Nordland, 67°57.337'N, 9°35.556'E, 1315 m, 6 May 2012; ZMBN 127301 (18 spec.), Nordland, 68°3.937'N, 9°28.129'E, 1712 m, 6 May 2012; ZMBN 127304 (24 spec.), Nordland, 67°17.06'N, 8°7.982'E, 1117 m, 8 May 2012; ZMBN 129496 (1 spec. on SEM stub), 67°17.0604'N, 8°7.9824'E, 1117 m, 8 May 2012; ZMBN 127264 (~30 spec.) 67°47.0'N, 07°43.9'E, 2025 m, 03 Jun 1981; ZMBN 127265 (~40 spec.), 65°39.5'N, 02°38.0'E, 2019 m, 07Jun 1981; ZMBN 127267 (~50 spec.), 63°35.6'N, 00°23.0'E, 2090 m, 15 Aug 1981; ZMBN 127266 (2 spec.), 64°16.9'N, 00°11.7'W, 2630 m, 14 Aug 1981; ZMBN 127268 (3 spec.), 62°29.5'N, 01°43.3'E, 604 m, 21 Jan 1982; ZMBN 127269 (10 spec.), 63°12.8'N, 03°07.3'E, 1003 m, 23 Aug 1982; ZMBN 127270 (6 spec.) 62°59.1'N, 03°13.1'E, 804 m, 27 Nov 1982; ZMBN 127272 (1 spec.), 64°26.1'N, 11°10.2'W, 400 m, 07 Jun 1983; ZMBN 127274 (2 spec.), 68°42.4'N, 10°29.5'W 2168 m, 13 Mar 1984; ZMBN 127276 (~100 spec.), 67°39.5'N, 11°36.7'W, 1811 m, 16 Mar 1984; ZMBN 127277 (~50 spec.), 62°35.1'N, 1°47.6'E, 656 m, 23 May 1984; ZMBN 127278 (1 spec.), 62°35.4'N, 01°47.7'E, 650 m, 23 May 1984; ZMBN 127279 (~ 25 spec.), 62°33.2'N, 01°49.2'E, 625 m, 21 Nov 1984; ZMBN 127280 (~50 spec.), 62°31.5'N, 01°26.6'E, 701 m, 08 Jan 1985; ZMBN 127281 (2 spec.), 63°45.2'N, 00°08.0'W, 2304 m, 11 Jan 1985 ; ZMBN 127282 (~20 spec.), 63°24.4'N, 00°20.3'E, 1880 m, 12 Jan 1985; ZMBN 127283 (~50 spec.), 63°02.9'N, 00°47.8'E, 1293 m, 12 Jan 1985; ZMBN 127284 (1 spec.), Faroes, 62°44.7'N, 06°46.9'W, 2538 m, 24 Jul 1986; ZMBN 127285 (~100 spec.), 69°01.4'N, 08°24.6'W, 879 m, 25 Jul 1986; ZMBN 129497 (1 spec. on SEM stub), 1243m, 70°40.68'N, 7°37.86'W, 27. Jul 1986, ZMBN 127286 (1 spec.), 69°36.4'N, 09°54.6'W, 2212 m, 26 Jul 1986; ZMBN 127289 (2 spec.), 70°26.2'N, 06°31.8'W, 2525 m, 28 Jul 1986; ZMBN 127290 (~20 spec.), 62°36.6'N, 01°34.4'E, 654 m, 15 Aug 1986; ZMBN 127292 (~25 spec.), 63°42.7'N, 00°09.7'W, 2259 m, 17 Aug 1986; ZMBN 127293 (~25 spec.), 63°35.1'N, 00°06.0'W, 2150 m, 17 Aug 1986; ZMBN 127294 (~50 spec.), 62°41.5'N, 01°45.4'E, 750 m, 17 Aug 1986; ZMBN 127291 (7 spec.), 63°28.8'N, 00°14.5'E, 1957 m, 16 Aug 1986; ZMBN 127306 (3 spec.) Aktivneset, 62°44.89'N, 3°1.98'E, 569 m, 24.09.2013; ZMBN 127314 (~20 spec.), 63°2.23'N, 4°41.34'E, 760 m, 30 Sep 2013.

##### Diagnosis.

Body ellipsoid, flattened dorsoventrally, up to 3 mm long. Body unpigmented; orange macrotubercles in live specimens, or brownish in fixed material. Head appendages smooth and digitiform. Tentacular cirri smaller than prostomial appendages. Dorsum with four longitudinal rows of large, spherical or pear-shaped sessile macrotubercles in a single transverse row per segment, from segment 2. Additional papillae on dorsum. First parapodia short and digitiform, with two rounded papillae on dorsal surface. Acicular lobe from segment 1. Eight to ten parapodial papillae. Ventral cirri digitiform shorter than acicular lobe tip. About eight compound chaetae with medium length blades (ca. ten times as long as wide); unidentate.

##### Description of specimens from the Nordic Seas.

*Measurements and general morphology.* Ellipsoid body, flattened dorsoventrally, up to 2.8 mm long, mm 0.6 wide and with up to 20 chaetigers. Segmentation not conspicuous. Live specimen with some orange granules in macrotubercles (Fig. 8D, E), brownish in fixed material.

*Head.* Head fused to first chaetiger (Figs 8D, 20A). Palps and lateral antennae conical, near five times longer than wide, lacking spurs or basal papilla (Fig. 20A–C). Median antenna conical, shorter than lateral antennae (Fig. 20A–C). Antenniform papillae present (Fig. 20B). Approximately 8–10 papillae confined by prostomial appendages. Tentacular cirri digitiform, similar to lateral antenna (Fig. 20B).

*Tubercles.* Medium-sized dorsal macrotubercles arranged in four longitudinal rows, one transverse row per segment, with exception of first chaetiger with only two macrotubercles (Figs 8D, E, 20A, B). Macrotubercles sessile, spherical and smooth in anterior segments, and pear-shaped in posterior segments (Figs 8D, 20A). Distance between dorsalmost rows is larger than these to lateralmost longitudinal rows (Figs 8D, 20A). Additional dorsal papillae, spherical, arranged in about four irregular transverse rows per segment along dorsal surface, with ca. 20 papillae per segment, and in four transverse rows per segment in ventrum (Fig. 15K, L, 20D, E).

*Parapodia.* Parapodia conical, with acicular lobe from chaetiger 1 (Fig. 20G). Ventral cirri conical, not protruding from parapodia. Six or seven spherical parapodial papillae (two in anterior surface, two in ventral side, two in posterior side and one or two dorsally; Fig. 5O)

*Chaetae*. All chaetae compound, with unidentate and finely serrated blades 8–20 times longer than wide. Up to 18–20 chaetae per fascicle (Fig. 20G, J, L). One straight acicula per parapodium.

*Pygidium*. A pair of piriform anal cirri, similar to posterior macrotubercles and digitiform medio-ventral anal papilla.

*Internal features*. Eyes not seen. Muscular pharynx not evident in live and fixed material (e.g., Fig. 8D).

*Reproductive features*. The holotype seems to be a female with some eggs inside the coelom (Fauvel 1911). Some females with oocytes (Fig. 8D); genital openings observed in females, between chaetigers 7 and 8 (Fig. 20 E, F).

##### Remarks.

The single type specimen of this species (in the Museum natural d’Histoire naturelle in Paris) is apparently lost, and the original description and drawings (Fauvel 1911) are simple and inadequate to describe all characters. The median antenna is not mentioned by Fauvel (1911), indicating it is probably small and similar to other prostomial papillae. The shape and number of dorsal and epithelial papillae are not cited either. The number of parapodial papillae is not explicitly mentioned and has been interpreted differently in the literature (e.g., Fauchald 1974, Aguado and Rouse 2006, Shimabukuro et al. 2017). There is indication of *S.philippi* comb. n. bearing 10–11 parapodial papillae (Fauchald 1974), or 20–22 parapodial papillae (e.g., Aguado and Rouse 2006, Shimabukuro et al. 2017). The first chaetigers bear at least two spherical dorsal papillae. According to original drawings, mid-body segments bear four dorsal parapodial papillae, and four are visible from the ventral side (Fauvel 1911, Figs 18, 19). Ventral cirri do not protrude from the acicular lobe, although both are well developed.

Several of the specimens found and re-examined from museum collections had previously been identified as belonging to this species. However, they were misidentified in several cases, as there are other similar species in the North East Atlantic (as reported herein) and, as mentioned, different interpretation of the parapodial papillae and chaetal morphology according to previous records. The description and drawings by Hartmann-Schröder (1971) do not correspond to this species since the original drawings of *S.philippi* comb. n. indicate that blades of chaetae are long (Fauvel 1911: Fig. 16) and these other records described, refer to or have illustrated short-blade chaetae.

The numerous specimens found from different localities in northern latitudes (and herein assigned to *S.philippi* comb. n.) resemble *Sphaerephesiaartabrensis* comb. n. in the overall appearance, the shape and distribution of dorsal macrotubercles and dorsal and ventral papillae, the chaetal morphology, the presence and arrangement of sexual structures/genital pores (Moreira and Parapar 2007). However, there are some consistent minor differences between the specimens examined from southern and northern localities. Specimens of *S.artabrensis* comb. n. are generally smaller (Iberian specimens are up to 1.75 mm long and Nordic specimens almost double in size), and bear a few more papillae in the prostomium, dorsum and parapodia (6–7 in specimens assigned to *S.philippi* comb. n., and 3–4 in *S.artabrensis* comb. n.). Specimens identified as *S.philippi* bear a well-developed acicular lobe from segment 1, instead of segment 2 as in members of *S.artabrensis* comb. n. (Moreira and Parapar 2007).

It would therefore be most interesting to study in detail more material from intermediate localities (not found so far), as well as the genetic structures of these populations to test if they actually belong to a single lineage with a range of morphological features, or if the northern and southern forms (herein *S.artabrensis* comb. n. and *S.philippi* comb. n.) truly belong to separate species. Given that the types of *S.philippi* comb. n. from the Kara Sea are lost, and that the description of the species is not very detailed, it is not guaranteed that the broadly distributed lineage found in Nordic waters (e.g., Fig. 1) belongs to this species. However, based in the presence of acicular lobe from first segment (bifurcated in original description), approximate number of parapodial papillae, and chaetal morphology (Fauvel 1911), we have opted for this possibility.

##### Distribution.

Arctic and Nordic Seas (Fauvel 1911, present study).

##### Habitat.

Sediments, at shelf to slope depths (Fauvel 1911, present study).

#### Sphaerephesia ponsi

Taxon classificationAnimaliaPhyllodocidaSphaerodoridae

Capa, Parapar & Moreira sp. n.

http://zoobank.org/40F740FF-6DFF-4083-8135-1A907657426E

**Figure 21.**
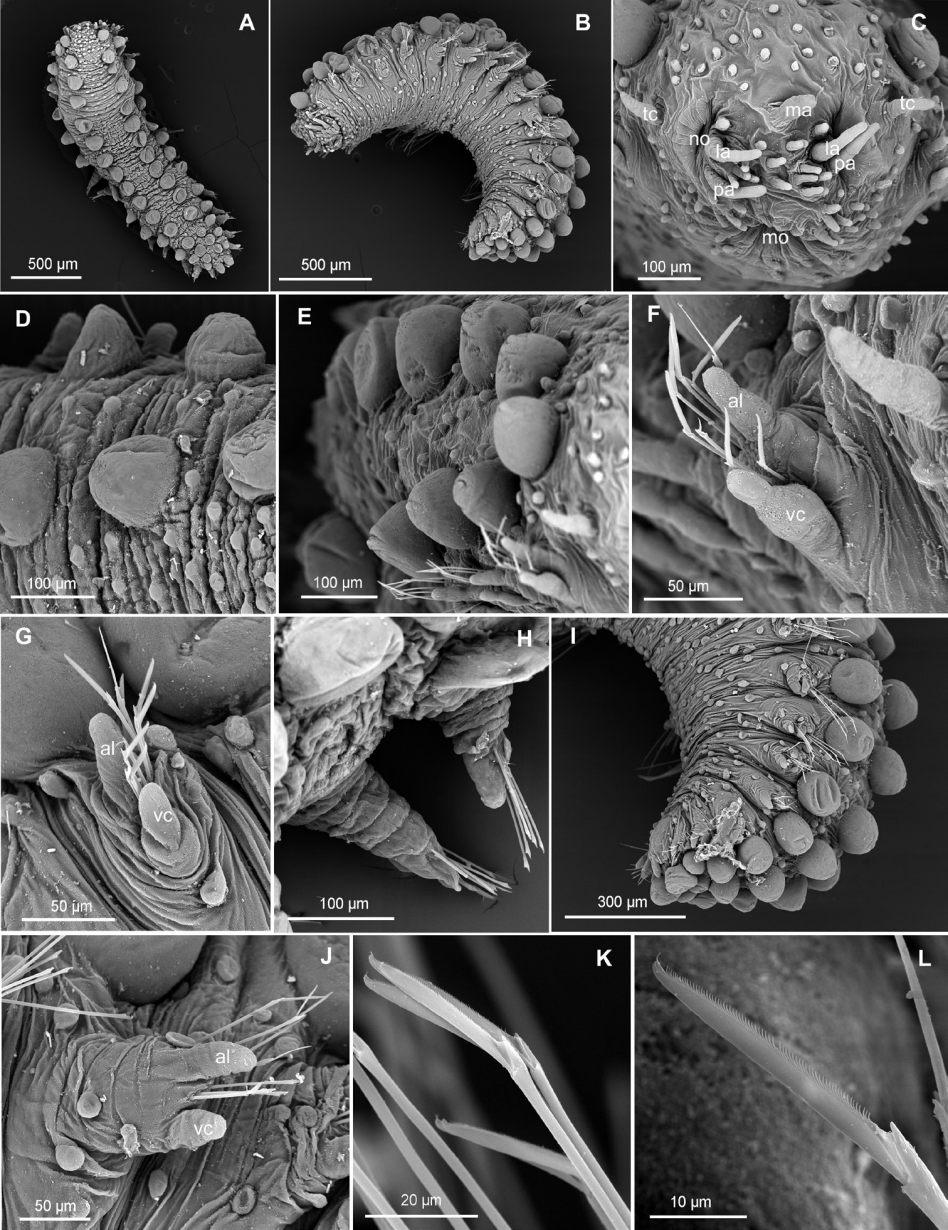
*Sphaerephesiaponsi* sp. n., scanning electron micrographs (Paratype, SMF 24841, SPH047). **A** Complete specimen, dorsal view **B** same, lateral view **C** head, anterior view **D** mid-body macrotubercles, frontal view **E** anterior chaetigers, frontal view **F** parapodium, chaetiger 1, anterior view **G** parapodium, chaetiger 2, side view **H** posterior parapodia, dorsal view **I** posterior chaetigers, lateral view **J** parapodium, chaetiger 7, anterior view **K** chaetae, mid-body chaetigers **L** chaetae, anterior chaetiger, detail.

Figs 5P , 15M, N , 21

##### Type locality.

Irminger Basin, SW of Iceland, North Atlantic Ocean, 63° 0.46'N, 28° 4'W, 1593 m.

##### Examined material.

**Holotype**: SMF 25295, Irminger Basin, SW of Iceland, 63°00.46'N, 28°04.09'W, 1593 m, 8 Sep 2011. **Paratypes**SMF 24841 (1 spec. used for DNA sequencing and SEM, SPH047), Irminger Basin, SW of Iceland, 63°00.46'N, 28°04.09'W, 1593 m, 8 Sep 2011; SMF 25296 (1 spec., on SEM stub), Irminger Basin, SW of Iceland, 63°00.46'N, 28°04.09'W, 1593 m, 8 Sep 2011.

##### Diagnosis.

Body ellipsoid, flattened dorsoventrally. Body unpigmented (fixed specimen). Head appendages smooth and digitiform. Tentacular cirri smaller than prostomial appendages. Dorsum with four longitudinal rows of large, sessile and pear-shaped macrotubercles in a single transverse row per segment, from segment 2. Additional papillae on dorsum and ventrum. Acicular lobe from segment 1. Parapodia with four papillae. Ventral cirri digitiform reaching tip of acicular lobe. Approximately eight compound chaetae with medium length blades (7–9 times as long as wide); unidentate.

##### Description.

*Measurements and general morphology*. Holotype 1.8 mm long, 0.5 mm wide; with 12 chaetigers (Fig. 21A). Body ellipsoid. Segmentation not conspicuous.

*Head.* Head fused to first segment (Fig. 21A–C). Head appendages digitiform (Fig. 21C). Palps and lateral antennae three times longer than wide, smooth, and lacking spurs or basal papillae (Fig. 21C). Median antenna two-thirds the length of lateral antennae, slightly wider (Fig. 21C). Antenniform papillae absent (Fig. 21C). Head papillae elliptical, ca. 20 papillae enclosed by prostomial appendages. Tentacular cirri digitiform, similar in shape and size to lateral antennae (Fig. 21C).

*Tubercles*. Four longitudinal rows of dorsal macrotubercles, in one transverse row per segment, from segment 2 (Fig. 21A–E). Macrotubercles, sessile, large, pear-shaped or with a terminal papilla (Fig. 21A–E). Distance between mid-dorsal rows larger than between these and lateral longitudinal rows of macrotubercles. Additional papillae, rounded, arranged in three or four irregular transverse rows along dorsal and ventral surfaces (Figs 15M, N, 21A). Ventrum with few papillae in holotype.

*Parapodia*. Parapodial conical, as long as wide (Fig. 21F–J). Ventral cirri conical, as long as maximum wide of parapodia, reaching the tip of the acicular lobe, or shorter (Fig. 21G, H, K). Acicular lobe from chaetiger 1. Anterior segments with two parapodial papillae, one ventral, near the base of parapodium and one posterior (Fig. 21F–H). Three or four parapodial papillae in mid-body segments, sub-equal, rounded (one dorsal, one or two anterior, one ventral near base of parapodium) (Figs 5P, 21G, J).

*Chaetae*. All chaetae compound, ca. eight in mid-body parapodia, with medium size blades (7–9 times as long as wide), unidentate, with finely serrated edge (Fig. 21L, M). One straight acicula per parapodia.

*Pygidium*. A pair globular anal cirri, similar to dorsal macrotubercles but smaller and digitiform medio-ventral anal papilla similar in length to lateral cirri (Fig. 21I).

*Internal features*. Holotype with everted proboscis, as long as 5–6 segments. Eyes not observed.

*Reproductive features*. No gametes, sexual structures, or genital pores observed.

##### Variation.

Paratypes 1–2 mm long, 0.3 mm wide, 15–17 segments. Epithelial parapodial papillae are more evident in paratypes, and ventral papillae include of ca. 20 rounded and sub-equal papillae, arranged in four irregular transverse rows per segment (Fig. 15N). Acicular lobe and ventral cirri are almost spherical in holotype while in the paratypes show longer and digitiform parapodial appendages. The rest of features are consistent among specimens studied.

##### Remarks.

*Sphaerephesiaponsi* sp. n. is distinguished from other congeners by the unique combination of following features: head appendages smooth and without spurs or basal papillae, antenniform papillae absent, macrotubercles in four longitudinal rows, pear-shaped and with terminal papillae, parapodial papillae with four spherical papillae, chaetae with blades up to nine times as long as wide. Other *Sphaerephesia* with ca. four parapodial papillae are *S.artabrensis* comb. n., *S.mamalaensis*, *S.longisetis* comb. n., and *S.amphorata*. *Sphaerephesiaamphorata* is clearly distinguished from *S.&nbsp;ponsi*&nbsp;sp.&nbsp;n. in the shape of the macrotubercles, with a long terminal papilla. *Sphaerephesiaponsi* sp. n. is distinguished from *S.artabrensis* comb. n. and *S.longisetis* in the length of chaetal blades, over ten times as long as wide in the former two species and shorter in the new species; and shorter in *S.mamalaensis* (up to six times as long as wide).

##### Etymology.

This new species is dedicated to Joan Pons, a researcher from the Mediterranean Institute of Advanced Studies (IMEDEA), Balearic Islands, colleague, and friend.

##### Distribution.

Only known from type locality, the Irminger Basin in the North East Atlantic.

##### Habitat.

Sediments at ca. 1600 m.

#### Sphaerephesia sibuetae

Taxon classificationAnimaliaPhyllodocidaSphaerodoridae

(Desbruyères, 1980) comb. n.

**Figure 22.**
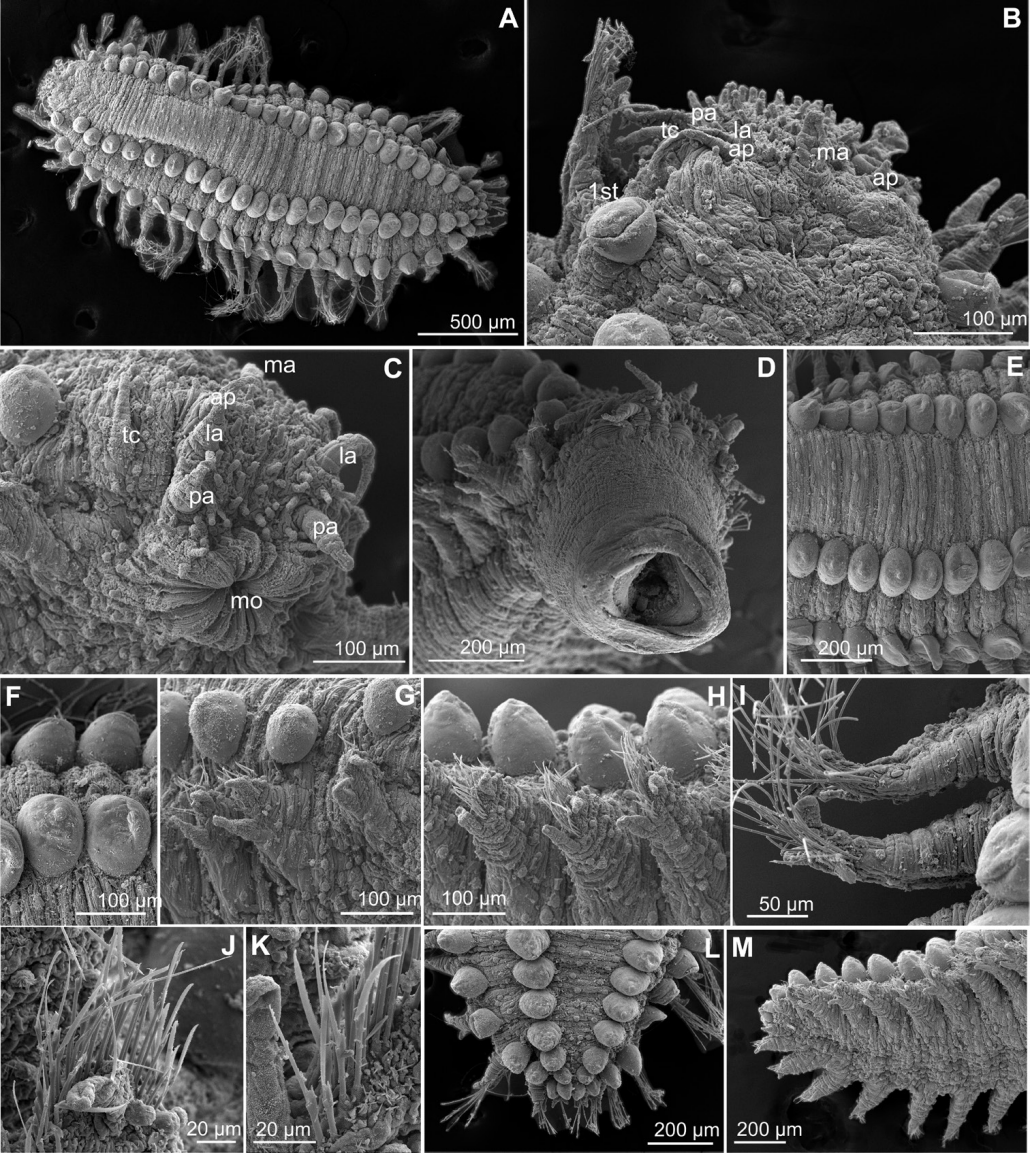
*Sphaerephesiasibuetae* (Paratypes, MNHN TYPE1284), scanning electron micrographs. **A**&nbsp;Complete specimen, dorsal view **B** head, dorsal view **C** head, ventral view **D** anterior end with everted proboscis, ventral view **E** mid-body chaetigers, dorsal view **F** dorsal macrotubercles, detail **G** parapodia, chaetigers 1–3, anterior view **H** mid-body parapodia, anterior view **I** mid-body parapodia, dorsal view **J&nbsp**;chaetal fascicle, mid-body chaetiger **K** chaetae, posterior chaetiger **L** posterior chaetigers, dorsal view **M**&nbsp;posterior chaetigers, ventral view.

Figs 5Q , 15O, P , 22

Sphaerodoropsis sibuetae Desbruyères, 1980: 226–229, Figs 9–10; Moreira et al. 2011: 26–28, Fig. 1.

##### Type locality.

Banc le Danois, Bay of Biscay, 44°5.2'N, 05°19.4'W, 1913 m.

##### Examined material.

**Holotype**: MNHN TYPE1284, Banc Le Danois, Bay of Biscay, 44°05.2'N, 5°19.4'W, 1913 m. **Paratypes**: same sample (6 specs, 2 on SEM stub).

##### Additional material.

(28 specs) **Iceland**, SMF 25297, (16 specs), Irminger Basin, 63°00.46'N, 028°04.09'W, 1593m, 8 Sep 2011; SMF 23906 (1 spec. used for DNA sequencing, SPH273), Irminger Basin, 63°00.46'N, 028°04.09'W, 1593m, 8 Sep 2011; SMF 23906 (1 spec. used for DNA sequencing, SPH 044), Irminger Basin, 63°00.46'N, 028°04.09'W, 1593m, 8 Sep 2011; SMF25298 (22 specs), Irminger Basin, 63°00.46'N, 028°04.09'W, 1593 m, 8 Sep 2011; SMF24855 (1 spec. used for DNA sequencing, SPH273), Irminger Basin, 63°00.46'N, 028°04.09'W, 1593 m, 8 Sep 2011; ZMBN 127325 (8 specs) South Iceland, 61°38.2'N, 16°27.7'W, 2355 m, 5 Jun 1983. **NW Iberian Peninsula**, MNCN 16.01/13268 (1 spec.), 42°31.66'N, 09°40.06'W, 1974–2034 m, 29 Sep 2008.

##### Diagnosis.

Body ellipsoid, flattened dorsoventrally, up to 5 mm long. Head appendages smooth, lacking spurs or basal papillae. Antenniform papillae present. Four longitudinal rows of macrotubercles, in a single transverse row per segment, from segment two. Macrotubercles sessile, pear-shaped or with terminal papilla. Small spherical papillae scattered on dorsal (four irregular transverse rows with ca. 30 papillae per segment) and on ventral surfaces (four irregular transverse rows per segment). Parapodia with acicular lobe from segment 1, with 16–19 rounded sub-equal papillae, apparently randomly arranged. Approximately 20–25 compound chaetae with long blades (9–13 times as long as wide).

##### Re-description of holotype.

*Measurements and general morphology*. Holotype 2.82 mm long, 0.5 mm wide and with 19 segments. Body with rounded anterior end, tapering from segment 6 to pygidium, circular in transverse section. Segmentation not conspicuous and pigmentation absent (Fig. 22A).

*Head.* Head fused to first segment (Fig. 22A, B). Prostomial appendages, palps, lateral antennae, and median antenna conical (Fig. 22B–D). Palps and lateral antennae three times longer than wide, smooth, and lacking spurs or basal papillae (Fig. 22B–D). Median antenna almost one third of the length of lateral antennae, digitiform (Fig. 22B, C). Antenniform papillae present (Fig. 22C). Head papillae rounded, numerous and randomly arranged (over 20 papillae enclosed by prostomial appendages). Tentacular cirri digitiform, shorter than lateral antennae and longer than median antenna (Fig. 22B, C).

*Tubercles*. Four longitudinal rows of dorsal macrotubercles, in one transverse row per segment, from segment 2 (Figs 15O, 22A). Macrotubercles, sessile, large pear-shaped or with a terminal papilla (Fig. 22A, E–H, L). Additional papillae, rounded, arranged in three or four irregular transverse rows along dorsal and ventral surfaces (Figs 15O, P, 22E, M).

*Parapodia*. Parapodial conical or cylindrical, three or four times longer than wide at mid-body (Fig. 22G–I). Ventral cirri digitiform, as long as maximum wide of parapodia, reaching the tip of the acicular lobe (Fig. 22H, I). Acicular lobe from chaetiger 1. Parapodial papillae (16–19) rounded, hemispherical and randomly distributed over their surface, being one, dorsal to acicular lobe, slightly larger than the rest. Three papillae on dorsal surface, 6–7 anterior, 3–4 ventral and 4–5 posterior (Figs 5Q, 22G–I).

*Chaetae*. All chaetae compound, ca. 15–25 in mid-body chaetigers, with long, unidentate compound chaetae with long blades (ca. 9–13 times their width showing some variation within parapodia) and with finely serrated edge (Fig. 22J, K). One straight acicula per parapodia.

*Pygidium*. A pair of globular anal cirri, similar to dorsal macrotubercles but smaller and digitiform medio-ventral anal papilla similar in length to lateral cirri (Fig. 22L, M).

*Internal features*. Muscular pharynx present through segments 1–4. Nuchal organs openings behind lateral antennae.

*Reproductive features*. Sexual structures or genital pores not observed in holotype.

##### Variation.

Size range of material examined: 2–5 mm long; 0.8–1.5 mm wide; with 17–31 chaetigers. Sexual dimorphism or reproductive features not observed in paratypes or additional material examined. Everted pharynx bare (Fig. 22D).

##### Remarks.

The species was originally considered as belonging to *Sphaerodoropsis* (Desbruyères 1980) even though the original description and iconography revealed that specimens have pear-shaped macrotubercles or with distal papilla; this was corroborated with the new scanning electron micrographs of paratypes provided herein (e.g., Fig. 22A).

*Sphaerephesiasibuetae* comb. n. differs from other species reported as presenting dorsal pear-shaped macrotubercles or with terminal papilla in the following combination of features: ellipsoid in shape, flattened dorsoventrally; head appendages smooth, without spurs or basal papillae; four irregular transverse rows of rounded papillae in both dorsum and ventrum, with ca. 30 papillae per segment; parapodia with ca. 16–19 papilllae and 15–25 compound chaetae with blades up to 13 times longer than wide in mid chaetigers. Other NEA similar congeners include *Sphaerephesiamultichaeta* sp. n., distinguished from *S.sibuetae* in the chaetal morphology (with shorter blades, up to seven times longer than wide) while *S.sibuetae* have longer blades (7–13 times as long as wide).

##### Distribution.

Newly recorded in southern Iceland (present study). Previously reported in Bay of Biscay and NW Iberian Peninsula (Moreira et al. 2011).

##### Habitat.

Sediments at 1400–2000 m (Desbruyères 1980, Moreira et al. 2011, present study).

#### Sphaerephesia stellifer

Taxon classificationAnimaliaPhyllodocidaSphaerodoridae

(Aguirrezabalaga & Ceberio, 2005) comb. n.

Fig. 5R , 15Q, R

Sphaerodoropsis stellifer Aguirrezabalaga & Ceberio, 2005: 13–16, Figs 3–4.

##### Type locality.

Capbreton Canyon, Bay of Biscay, 43°42.01'N, 2°18.52'W, 990 m.

##### Material examined.

**Holotype**: MNCN 16.01/9051, Bay of Biscay, Capbreton Canyon, 43°42.01'N, 2°18.52'W, 990 m; **Paratype**: MNHN, apparently lost.

##### Diagnosis.

Body ellipsoid, flattened dorsoventrally, up to 3.2 mm long. Head appendages digitiform, lacking spurs. Median antenna shorter than palps and lateral antennae. Antenniform papillae present. Dorsum with four longitudinal rows of macrotubercles, in a single transverse row per segment, from segment 2. Macrotubercles sessile pear-shaped. Additional small spherical papillae on dorsum, arranged in 3–4 transverse rows per segment, each with ca. ten papillae. Ventrum with papillae, in 3–4 transverse rows per segment. Parapodia conical, with 7–10 sub-equal papillae uniformly distributed. Ventral cirri digitiform not surpassing acicular lobe tip. Approximately 6–12 compound chaetae with medium length blades (near six times their width), showing small gradation within fascicles; unidentate and subtle spinulation along its cutting margin.

##### Variation.

According to the original description, the range of variation of material examined is 18–20 chaetigers, 1.7–3.1 mm long and 0.6–0.8 mm wide.

##### Remarks.

*Sphaerephesiastellifer* comb. n. was described as a new species due to the star-shaped epithelial papillae, instead of spherical, oval of hemispherical, typical from other sphaerodorids. Revision of the holotype did not ensure this particular condition any longer, and instead it has the characteristic pear-shaped dorsal macrotubercles, resembling other *Sphaerephesia* species. The original drawings even include a close up of a macrotubercle with a small terminal papilla. Therefore, we suspect the shape described by Aguirrezabalaga and Ceberio (2005) as ‘*macrotubercles with funnel-like distal invaginations*’ can be attributable to temporary collapse of the tubercles and it has now reverted to the typical pear-shaped tubercles. Collapsed epithelial tubercles has been observed in specimens of different species, indicating it may be related to particular fixation procedures and making it a not reliable diagnostic feature.

A combination of features may allow distinguishing *S.stellifer* comb. n. from other NEA *Sphaerephesia*. These include the ellipsoid body shape, presence of pear-shaped macrotubercles, 3–4 transverse rows of additional papillae over dorsum and ventrum, conical parapodia with ca. ten papillae, and 6–12 chaetae, near six times as long as wide. Nevertheless, *Sphaerephesialaureci* comb. n. is a similar species with a more elongated body and also longer parapodia (Desbruyères, 1980); further studies should conclude if these features are enough to separate representatives of these species, or if *S.stellifer* comb. n. is in fact a junior synonym of *S.laureci* comb. n.

##### Distribution.

Capbreton Canyon, Bay of Biscay (Aguirezzabalaga and Ceberio 2005).

##### Habitat.

Soft bottoms at depths of between 990–1040 m.

#### Sphaerephesia

Taxon classificationAnimaliaPhyllodocidaSphaerodoridae

sp. 1

Fig. 8E

##### Diagnosis.

Body ellipsoid, flattened dorsoventrally, up to 3 mm long. Body unpigmented; macrotubercles in live specimens orange, or brownish in fixed material. Head appendages smooth, digitiform. Tentacular cirri smaller than prostomial appendages. Dorsum with four longitudinal rows of large, spherical, or pear-shaped sessile macrotubercles in a single transverse row per segment, from segment 2. Additional papillae on dorsum. First parapodia short and digitiform, with two rounded papillae on dorsal surface. Acicular lobe from segment 1. Eight to ten parapodial papillae. Ventral cirri digitiform shorter than acicular lobe tip. Approximately eight compound chaetae with medium length blades (ca. ten times as long as wide); unidentate.

##### Examined material.

(5 specs) **Iceland Sea**, SMF 23908 and 23907 (2 spec, used for DNA sequencing, SPH056 and SPH057, respectively), NE Iceland, 66°32.63'N, 12°52.48'W, 317.2 m, 22 Sep 2011; **Svalbard**, ZMBN 127327 (1 spec. used for DNA sequencing, SPH 296), Svalbard, 81°0.066'N, 19°17.802'E, 168 m, 01 Sep 2009; **Barents Sea**, ZMBN 127326 (1 spec. used for DNA sequencing, SPH292); Finnmark, 72°18.59'N, 32°20.48'E, 312 m, 04 Aug 2013; **Skagerrak**, ZMBN 125432 (1 spec. used for DNA sequencing SPH 297 photographed alive Fig. 8D), 58°40.806'N, 10°23.982'E, 238 m, 16 May 2009; ZMBN 127328 (1 spec. used for DNA sequencing, SPH 298.) 58°40.806'N, 10°23.982'E, 237 m, 16 May 2009.

##### Remarks.

These specimens were initially identified as *Sphaerephesiaphilippi* comb. n., as there seems to be no noticeable morphological differences between them. However, analyses of molecular data recovered some of the specimens forming a different lineage, sister group to a clade containing *S.discolis* and *S.sibuetae* (Fig. 1). We would like to corroborate this information with further data before describing it as a new species.

##### Distribution.

Iceland Sea, Svalbard, Barents Sea, and Skagerrak.

##### Habitat.

Continental shelf sediments (100–350 m).

#### Sphaerodoridium

Taxon classificationAnimaliaPhyllodocidaSphaerodoridae

Lützen, 1961

Sphaerodoridium Lützen, 1961; Fauchald 1974: 270 (in part).
Sphaerodoropsis Hartman & Fauchald, 1971: 69 (in part); Fauchald 1974: 261 (in part); Borowski 1994: 23 (in part); Moreira 2012: 30 (in part); Capa et al. 2014: 17 (in part).

##### Type species.

*Sphaerodorumclaparedii* Greeff, 1866.

##### Diagnosis.

Body short and ovoid, some forms slender. Prostomial appendages short, spherical or digitiform; median antenna shorter or as long as lateral antennae; antenniform papillae absent or present. Macrotubercles sessile or stalked; smooth, without terminal papilla, arranged in more or less clear longitudinal rows, one transverse row per segment, with at least seven macrotubercles each. Microtubercles absent. Additional papillae over body surface and parapodia. Parapodia with compound chaetae; stout hooks in anterior chaetigers absent.

##### Remarks.

In the present study, a clade was recovered containing species previously assembled under *Sphaerodoropsis* Group 2 (Borowski 1994) (that is with sessile dorsal macrotubercles arranged in six or more longitudinal rows, and only one transverse row per segment), nested among other previously considered as *Sphaerodoridium* (that is with stalked macrotubercles) with more than seven longitudinal rows of macrotubercles (Fig. 1). This provides evidence that the macrotubercle morphology (i.e., sessile or stalked dorsal macrotubercles) is not a valid character separating reciprocally monophyletic groups. Instead, members of this clade should be considered as one genus containing sphaerodorids with more than six longitudinal rows of macrotubercles, arranged in a single transverse row per segment. Note that Borowski (1994) included one species bearing six macrotubercles in the first chaetiger under the artificial Group 2, like others in this group, but otherwise more than six. It is therefore here interpreted as belonging to the newly interpreted *Sphaerodoridium*: that is with more than six longitudinal rows of macrotubercles.

The type species of the genus *Sphaerodoridium*, *S.claparedii*, and the type species of *Sphaerodoropsis*, *Sphaerodoropsissphaerulifer* share this feature. However, *Sphaerodoridium* was erected previous (Lützen 1961) to *Sphaerodoropsis* (Hartman and Fauchald 1971). Therefore, we consider this clade should bear the genus name *Sphaerodoridium*.

The diagnosis of the genus *Sphaerodoridium* differs from the original concept. In order to accommodate these, several systematic (all and only sphaerodorid species presenting seven or more longitudinal rows of macrotubercles, arranged in one transverse row per segment) are consider belonging to this group and nomenclatural changes are required.

Species currently considered within *Sphaerodoridium* are:

*Sphaerodoridiumaestuarum* (Averincev, 1990), comb. n.

Type locality: Laptev Sea, 3–6.5 m.

*Sphaerodoridiumamoureuxi* Aguirrezabalaga & Cebeiro, 2005

Type locality: Capbreton Canyon, Bay of Biscay, 984–1029 m.

*Sphaerodoridiumandamanense* (Bakken, 2002), comb. n.

Type locality: Off Phi Phi Island, Andaman Sea, Thailand, 29 m.

*Sphaerodoridiumauranticum* (Capa & Rouse, 2015), comb. n.

Type locality: Yonge Reef, Great Barrier Reef, Australia, 4–12 m.

*Sphaerodoridiumbalticum* (Reimers, 1933), comb. n.

Type locality: Kiel, Baltic Sea, 6–8 m.

*Sphaerodoridiumbengalorum* (Fauchald, 1974), comb. n.

Type locality: Porto Novo, Madras, India, 1.5 m.

*Sphaerodoridiumbenguellarum* (Day, 1963), comb. n.

Type locality: Off eastern coast of South Africa, 172 m.

*Sphaerodoridiumcampanulata* Borowski, 1994.

Type locality: Peru Basin, Pacific Ocean, 4163 m.

*Sphaerodoridiumclaparedii* Greeff, 1866

Type locality: Dieppe, English Channel, France.

*Sphaerodoridiumevgenovi* Gagaev, 2015

Type locality: Barents Sea, 226 m.

*Sphaerodoridiumgudmunduri* (Moreira & Parapar, 2012), comb. n.

Type locality: North of Iceland, 97 m.

*Sphaerodoridiumguerritai* Moreira & Parapar, 2015

Type locality: North of Iceland, 600 m.

*Sphaerodoridiumkatchemakensis* (Kudenov, 1987), comb. n.

Type locality: Alaska, USA, 10 m.

*Sphaerodoridiumkolchaki* Gagaev, 2015

Type locality: Barents Sea, 290 m.

*Sphaerodoridiumkupetskii* Gagaev, 2015

Type locality: Canada Basin, 1004 m.

*Sphaerodoridiumjaponicum* Ozolin’sh, 1987

Type locality: Sea of Japan, 33–62 m.

*Sphaerodoridiumlutzeni* Kudenov, 1987

Type locality: E Florida, Gulf of Mexico, 34 m.

*Sphaerodoridiumminutum* (Webster & Benedict, 1887)

Type locality: Off New England, USA, shelf depths.

*Sphaerodoridiumoctopapillatum* (Hartmann-Schröder, 1965), comb. n.

Type locality: Off Galera, Chile, 260 m.

*Sphaerodoridiumpolypapillatum* (Hartmann-Schroder & Rosenfeldt,1988), comb. n.

Type locality: King George Island, Antarctica, 263 m.

*Sphaerodoridiumsphaerulipher* (Moore, 1909), comb. n.

Type locality: Monterey Bay, California, USA.

*Sphaerodoridiumuzintunensis* (Kudenov, 1987), comb. n.

Type locality: Alaska, USA, 3 m.

#### Sphaerodoridium amoureuxi

Taxon classificationAnimaliaPhyllodocidaSphaerodoridae

(Aguirrezabalaga & Ceberio, 2005) comb. n.

Figs 5S , 23A, B

Sphaerodoropsis amoureuxi Aguirrezabalaga & Ceberio, 2005: 10–13, figs 1, 2; Moreira et al. 2011: 28–29, fig. 2; Moreira 2012, 36–39, fig. 11.

##### Type locality.

Capbreton Canyon, Bay of Biscay, 984–1029 m.

##### Material examined.

**Holotype**: MNCN 16.01/8925, Capbreton Canyon, Bay of Biscay, 984–1029 m. **Paratype**: not found in MNHN, seems to be missing.

##### Additional material.

(1 spec) **NW Iberian Peninsula**: MNCN 16.01/13269 (1 spec.), 43°36.22'N, 08°52.84'W, 200 m, 26 Sep 2004.

##### Diagnosis.

Body short and cylindrical, less than 3.5 mm long. Palps, lateral antennae, and tentacular cirri, with spurs or basal papillae (lateral antennae 6–8). Median antenna smooth, shorter than other head appendages. Antenniform papillae present. Dorsal macrotubercles sessile, almost spherical, arranged in eight longitudinal rows in a single transverse row per segment, from segment 3. Dorsum with additional large spherical papillae, arranged between macrotubercles in more or less clear transverse rows, 25–30 papilla per segment. Ventrum with ca. 20 small spherical papillae per segment in midbody, arranged in 3–4 transverse rows per segment. Parapodia with three papillae; two sub-equal spherical papillae on ventral and anterior surfaces, and one postchaetal terminal papilla. Acicular lobe from segment 3. Approximately 6–8 compound chaetae with medium blades (up to seven times as long as wide), showing slight intra-fascicle variation in size.

##### Remarks.

This species has been described in detail (Aguirrezabalaga and Ceberio 2005) and some additional intraspecific variation has been reported (Moreira et al. 2011) and incorporated in the diagnosis. The antenniform papillae can be contracted and not evident in some specimens; variation regarding the number of parapodial papillae with three instead of 2–4. The postchaetal lobes mentioned in the original description and by Moreira et al. (2011) are herein considered a distal postchaetal papilla. Some macrotubercles seem to have a short stalk (or at least they are almost spherical) in the types re-examined. *Sphaerodoropsisamoureuxi* comb. n. belongs to the clade of sphaerodorids with over six longitudinal rows of macrotubercles arranged in a single segmental transverse row. Females with visible eggs, sexual or sexual structures not observed.

**Figure 23.**
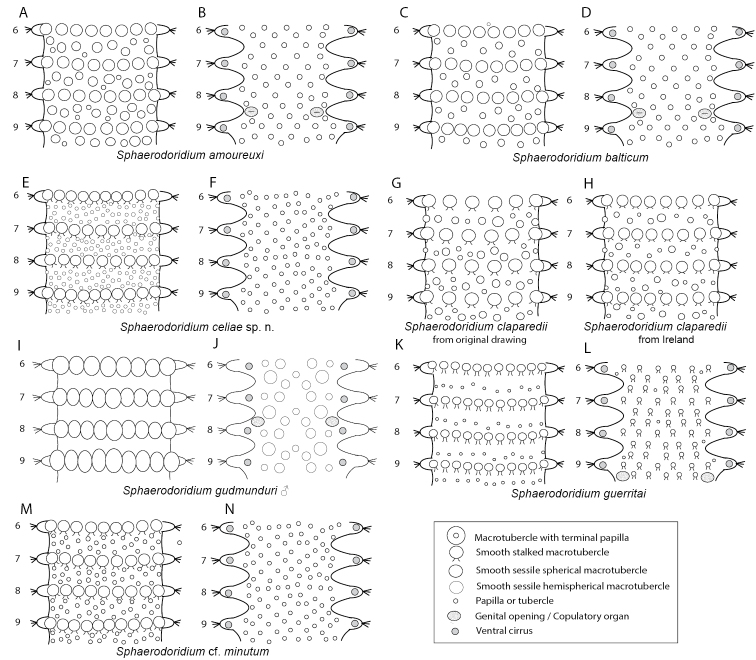
Stylized drawings of selected dorsal and ventral segments of species of *Sphaerodoridium*, showing number and arrangement of epithelial tubercles and papillae.

*Sphaerodoridiumamoureuxi* comb. n. differs from other congeners in the number of macrotubercles (up to eight, Fig. 23A), presence of spurs and basal papillae in paired head appendages, and shape, number of papillae per segment (spherical, 24–30, Fig. 5S) and chaetal morphology, with blades up to seven times as long as wide. The description of this species was justified based in the number of head appendages (Aguirrezabalaga and Ceberio 2005), a feature that seems to show some variability (Moreira et al. 2011), and the number of parapodial lobes (Aguirrezabalaga and Ceberio 2005), but these could have a different misinterpretation and be considered as parapodial papillae (Moreira et al. 2011 and herein).

##### Distribution.

Capbreton Canyon, Bay of Biscay (Aguirrezabalaga and Ceberio 2005); NW Iberian Peninsula (Moreira et al. 2011).

##### Habitat.

Soft bottoms between 100–1030 m (Moreira 2012).

#### Sphaerodoridium balticum

Taxon classificationAnimaliaPhyllodocidaSphaerodoridae

(Reimers, 1933) comb. n.

**Figure 24.**
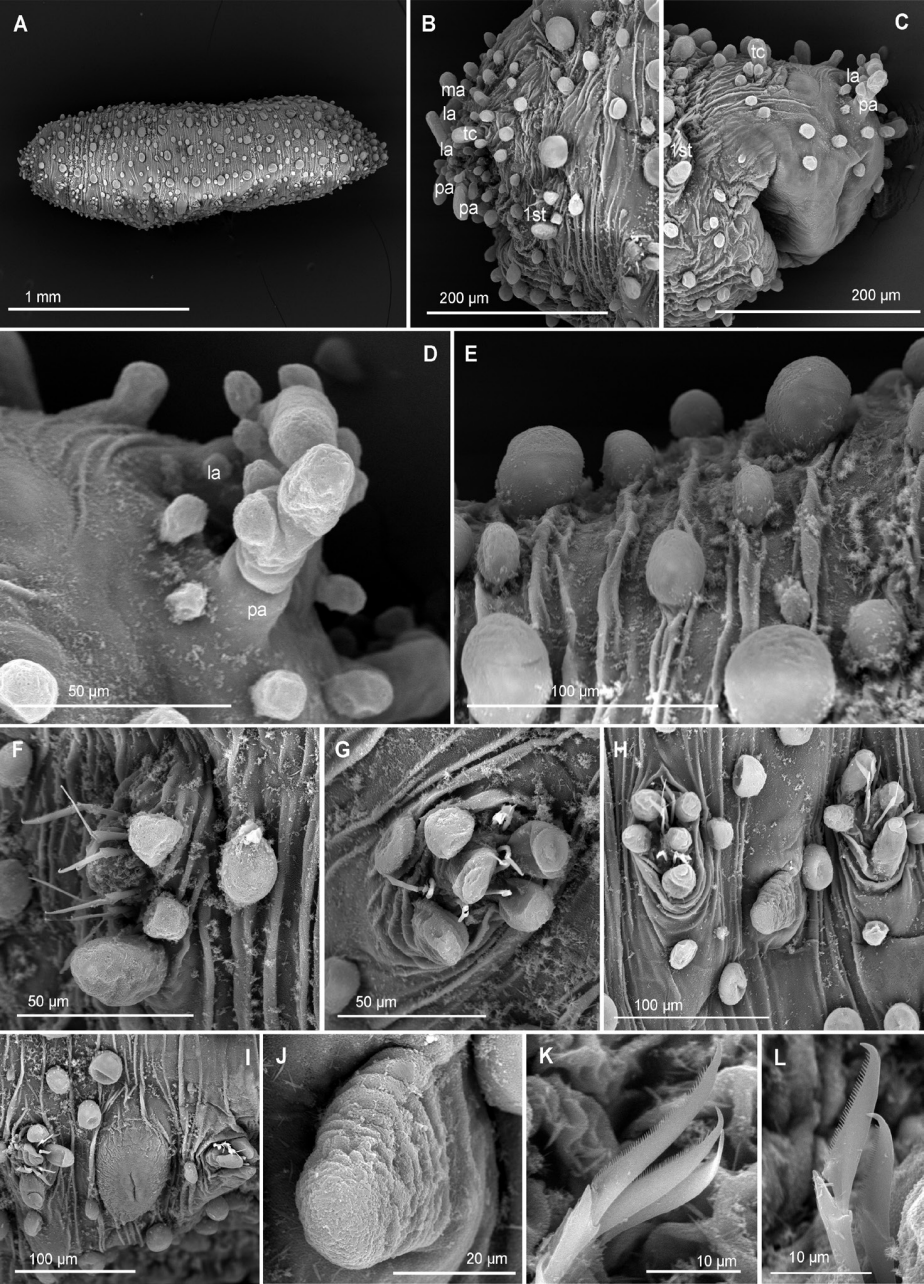
*Sphaerodoridiumbalticum*, from Norwegian Sea (ZMBN 129499 , SPH281), scanning electron micrographs. **A** Complete specimen, dorsal view **B** head, lateral view **C** palps with spurs and lateral antennae (behind), detail **D** epithelial tubercles over dorsum **E** parapodium, chaetiger 1 **F** parapodium, chaetiger 4 **G** parapodia, chaetigers 7 and 8, with a sexual structure in between (male) **H** genital pores between parapodia chaetiger 7 and 8 (female) **I** male genital organ, detail **J** chaetae, chaetiger 1 **K, L**&nbsp;chaetae, mid-body parapodia.

Figs 5T , 23C, D , 24

Sphaerodorum balticum Reimers, 1933: 41–110, 45 figs

##### Type locality.

Kiel, Baltic Sea, 6–8 m.

##### Material examined.

(16 specs) **Barents Sea**: ZMBN 127332 (1 spec. used for DNA sequencing, SPH 280), 70°17.79'N, 31°18.83'E, 217 m, 18 Aug 2013; **Norwegian Sea**: ZMBN 127331 (11 spec.), Mørebanken, 62°45.5'N, 5°31.56'E, 91 m, 04 Oct 2012; ZMBN 127329 (1 spec. used for DNA sequencing, SPH281), Mørebanken, 62°45.5'N, 5°31.56'E, 91 m, 04 Oct 2012; ZMBN 127330 (1 spec. used for DNA sequencing SPH282), Mørebanken, 62°45.5'N, 5°31.56'E, 91 m, 04 Oct 2012; ZMBN 129499 (1 spec. on SEM stub), Mørebanken, 62°45.5'N, 5°31.56'E, 91 m, 04 Oct 2012. **Kattegat**: NHMD 302862 (1 spec.), Kattegat, Samsø, NW of Bosserne, 23–26 m, 18 Jul 1969; NHMD 302861 (1 spec.), Kattegat, Samsø, NW Møgelskår, Nordby, 14 Jul 1979.

##### Diagnosis.

Body short and ellipsoid. Prostomial appendages digitiform, smooth, longer than wide. Palps and lateral antennae with basal spurs or basal papillae, the later with 3–4 spurs. Median antenna as long as or slightly shorter than other head appendages. Antenniform papillae absent. Eight to nine longitudinal rows of spherical and sessile macrotubercles in one transverse row per segment. Additional spherical papillae arranged in three transverse rows per segment, in dorsum and ventrum. Parapodia with acicular lobe from chaetiger 3, digitiform; ventral cirri digitiform, projecting well beyond acicular lobe; four spherical parapodial papillae. Compound chaetae with medium length blades (6–7 times as long as wide), showing little dorso-ventral gradation; unidentate, finely serrated.

##### Re-description of NEA material.

*Measurements and general morphology.* Body ellipsoid, with rounded anterior and posterior ends, with convex dorsal surface and slightly flat ventrum; cross section almost circular (Fig. 24A). Segmentation inconspicuous and pigmentation absent. Measuring 1.3–2.2 mm in length and near 0.5 mm wide; with 10–18 chaetigers.

*Head.* Prostomium fused to first segment. Prostomial appendages including palps and lateral antennae digitiform, similar in shape and size (Fig. 24B). Palps and lateral antennae with 3–4 basal papillae or spurs (Fig. 24C, D). Median antenna shorter than lateral appendages, or similar to other rounded prostomial papillae, without spurs. Tentacular cirri, similar in size and shape to median antenna (Fig. 24C).

*Tubercles*. Dorsal macrotubercles, sessile, spherical, smooth. Arranged in 8–9 longitudinal rows from second chaetiger to posterior segments, and one transverse dorsal row per segment (Fig. 24A). All macrotubercles similar in size. Additional spherical dorsal papillae arranged in two or three irregular transverse rows, ca. 20 papillae in mid-body segments (Figs 23C, 24A). Ventral papillae, similar to dorsal in shape and size, arranged in three transverse rows per segment (Figs 23D, 24A).

*Parapodia*. Parapodia conical, as long as wide. Ventral cirri digitiform. Acicular lobe similar or slightly shorter, similar in shape and size to parapodial papillae. Four parapodial papillae, two dorsal and two posterior, one behind the chaetal fascicle and another one closer to the base (Figs 5T, 24F–I).

*Chaetae*. Five to seven compound chaetae on each parapodium, with fine and medium size blades (ca. 5–8 times as long as wide), unidentate, finely serrated (Fig. 24F–H, K, L). A single straight acicula per parapodium.

*Pygidium*. Pygidium with paired anal cirri resembling macrotubercles and medio-ventral digitiform anal papilla.

*Internal features.* Two dark eyes visible dorsally inside the head. Pharynx extending over two chaetigers.

*Reproductive features.* Sexual structures between parapodia of chaetigers 8 and 9. They resemble a large ventral cirrus or a hemispherical knob (Fig. 24A, H, J).

##### Remarks.

This species has been widely recorded across the North and Baltic seas (Hartmann-Schröder 1996) but giving the similarity with other species also present in the area these records require revisions. Other similar species reported from European waters are Sphaerodoridiumcf.minutum (Webster & Benedict, 1887) (see below) that bears 10–14 stalked macrotubercles per segment. One of the diagnostic features of this species is the presence of spurs in palps and lateral antennae. These are only clearly recognised in specimens with a relaxed head and appendages (e.g., Fig. 24C, D) but are not obvious on specimens with contracted anterior end (e.g., Fig. 24B)

##### Distribution.

New records for the Barents Sea, Norwegian Sea, and Kattegat. Reported in the North and Baltic seas (Hartmann-Schröder 1996).

##### Habitat.

Sandy and muddy sediments, 6–200 m (Hartmann-Schröder 1996, present study).

#### Sphaerodoridium celiae

Taxon classificationAnimaliaPhyllodocidaSphaerodoridae

Moreira, Capa & Parapar sp. n.

http://zoobank.org/DDFF262D-F131-4DC3-8A3B-68362EF256ED

**Figure 25.**
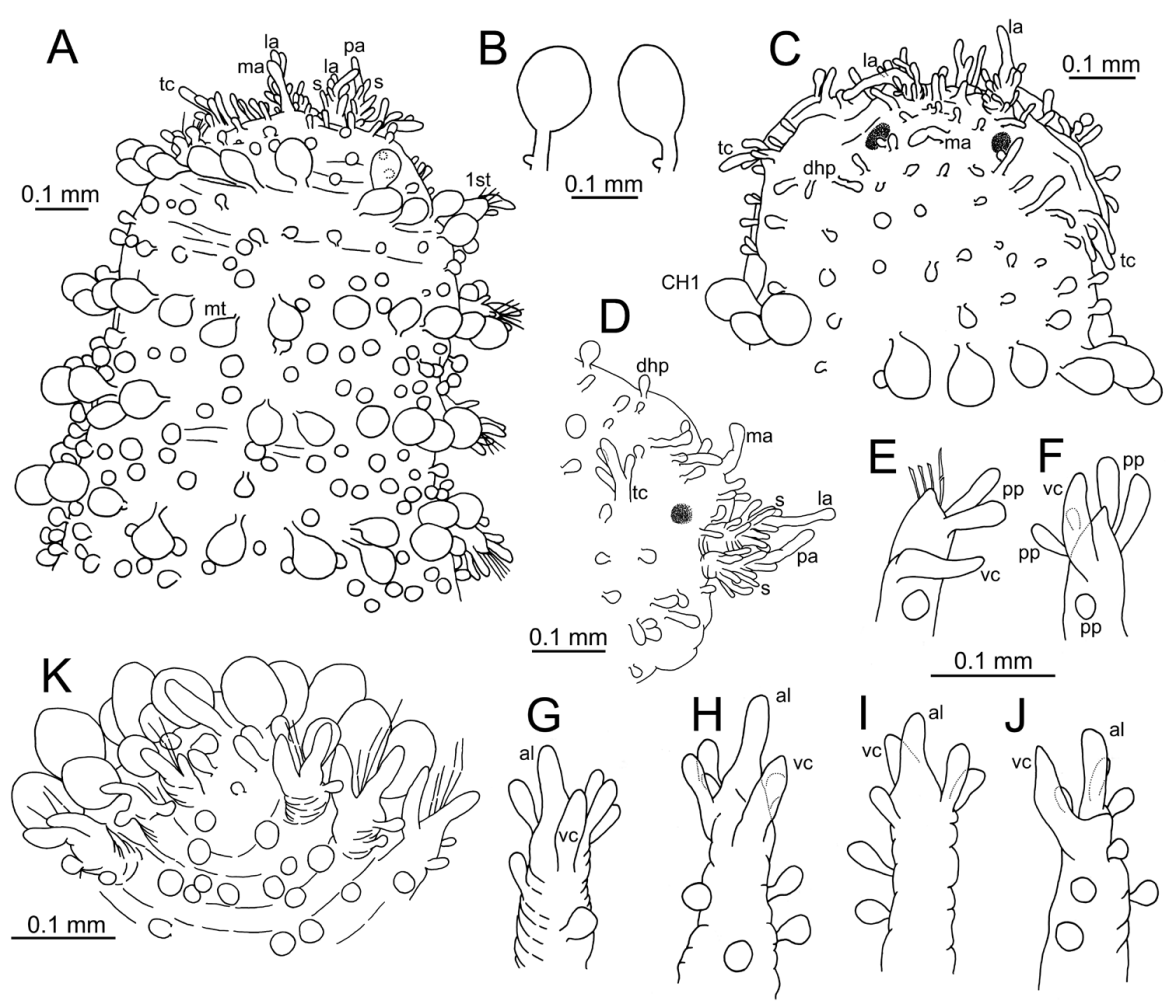
*Sphaerodoridiumceliae* sp. n., line drawings (holotype, IINH 38817,: **A, B, E–K**; paratype, IINH 38812: **C, D**). **A** Anterior end, dorsal view **B** macrotubercles, chaetigers 7 and 19 **C, D** anterior end, dorsal and lateral view, respectively **E–J** parapodia **E** chaetiger 1, left side, ventral view **F** chaetiger 2, left side, ventral view **G** chaetiger 4, left side, ventral view **H** chaetiger 9, right side, ventral view **I**&nbsp;chaetiger 11, right side, dorsal view **J** chaetiger 23, right side, ventral view **K** posterior end, ventral view.

**Figure 26.**
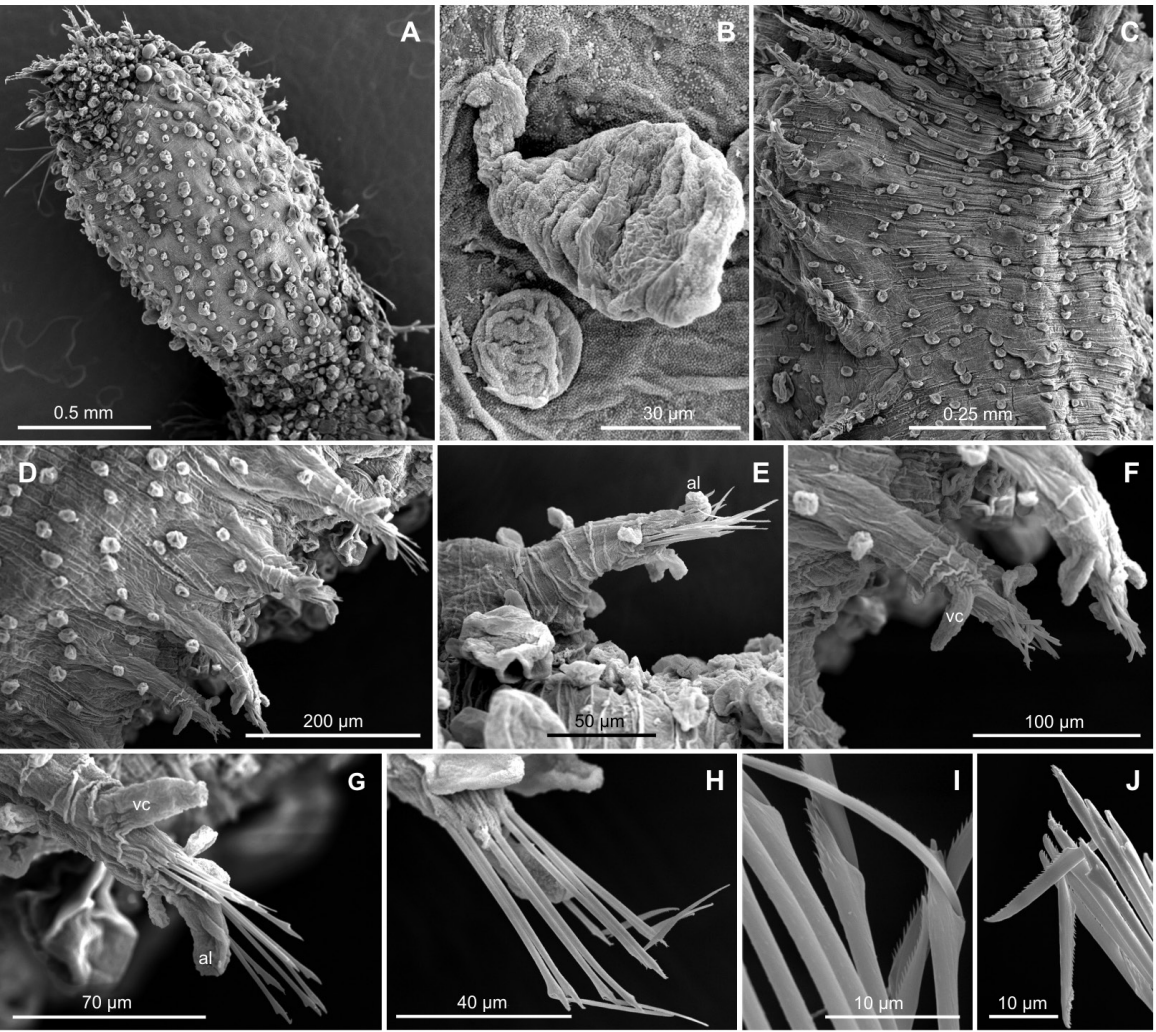
*Sphaerodoridiumceliae* sp. n. (IINH 38795), scanning electron micrographs. **A** Anterior end, dorsal view **B** macrotubercle and stalk, detail **C** mid-body chaetigers, ventral view **D** parapodia, mid-body chaetigers, ventral view **E** parapodium, mid-body chaetiger, dorsal view **F** parapodia, mid-body chaetigers, ventro-lateral view **G** parapodium, mid-body chaetigers, detail of ventral cirrus and chaetae disposition **H** chaetal fascicle, mid-body chaetiger **I** chaetae, detail of shaft **J** chaetae, detail of blades.

**Figure 27.**
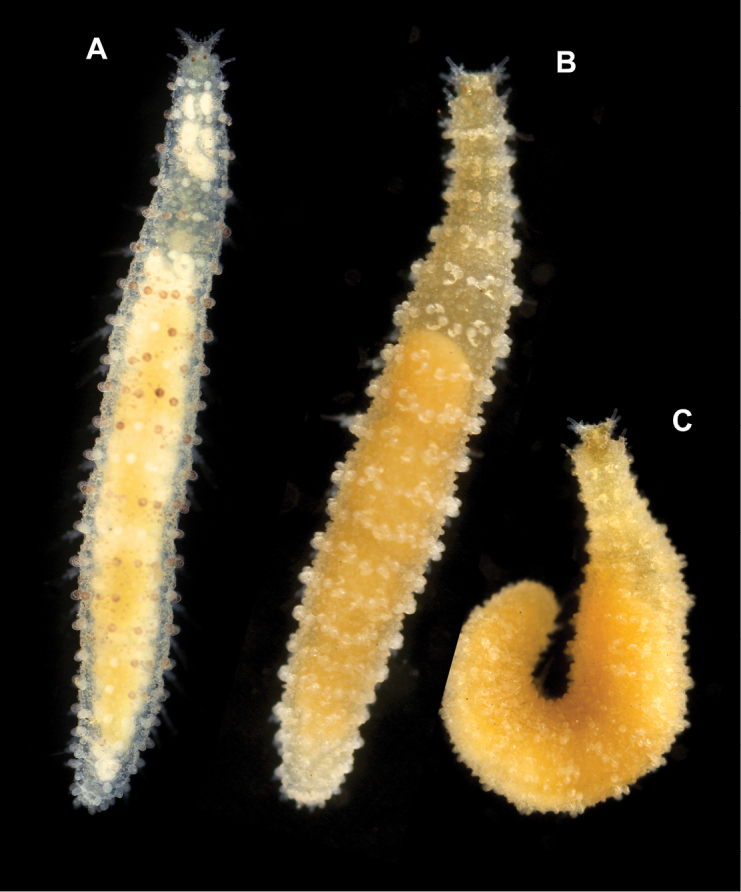
Photographs of live specimens, dorsal view. **A***Sphaerodoridiumceliae* sp. n., from Skagerrak (ZMBN 125434, SPH 316) **B, C**Sphaerodoridiumcf.minutum from the UK (ZMBN 127346 SPH 320, ZMBN 127347 SPH 321, respectively).

Figs 5U , 23E, F , 25 , 26 , 27A

##### Type locality.

NW Iceland, 67°30.76'N, 24°10.03'W, 1012 m.

##### Material examined.

Type series: **Holotype**: IINH 38817, Iceland, 67°30.72'N, 24°10.03'W, 1012 m, 25 Aug 1999. Paratypes (535 specs).: IINH 38795 (6 specs on SEM stub), 63°08.60'N, 22°14.80'W, 248 m, 30 June 1996; IINH 38796 (1 spec.), 67°0.25'N, 17°25.01'W, 248 m, 10 July 1994; IINH 38797 (1 spec.), 67°55.91'N, 15°21.29'W, 1098 m, 13 July 1994; IINH 38798 (3 specs), 66°50.20'N, 16°15.74'W, 227 m, 15 July 1994; IINH 38799 (2 specs), 66°43.92'N, 16°50.54'W, 150 m, 15 July 1994; IINH 38800 (1 spec.), 68°01.13'N, 20°39.28'W, 970 m, 31 July 1995; IINH 38801 (34 specs), 63°15.00'N, 17°59.40'W, 175 m, 24 Aug 1995; IINH 38802 (26 specs), 63°30.12'N, 17°42.07'W, 120 m, 25 Aug 1995; IINH 38803 (20 specs), 63°25.06'N, 16°50.40'W, 272 m, 25 Aug 1995; IINH 38804 (22 specs), 62°20.17'N, 16°59.40'W, 2074 m, 28 Aug 1995; IINH 38805 (6 specs), 65°21.22'N, 27°25.43'W, 513 m, 24 Aug 1996; IINH 38806 (19 specs), 65°31.14'N, 26°13.11'W, 157 m, 28 Aug 1996; IINH 38807 (25 specs), 65°39.90'N, 26°11.33'W, 166 m, 28 Aug 1996; IINH 38808 (5 specs), 65°42.18'N, 25°16.99'W, 160 m, 29 Aug 1996; IINH 38809 (31 specs), 65°08.01'N, 23°36.17'W, 120 m, 30 Aug 1996; IINH 38810 (207 specs), 63°45.60'N, 14°50.60'W, 216 m, 5 July 1997; IINH 38811 (33 specs), 67°11.02'N, 21°45.68'W, 230 m, 21 Aug 1999; IINH 38812 (42 specs); IINH 38813 (9 specs), 66°10.23'N, 12°00.94'W, 243 m, 14 July 2001; IINH 38814 (11 specs), 65°50.34'N, 12°01.27'W, 192 m, 14 July 2001; IINH 38815 (1 spec.), 68°00.92'N, 009°14.78'W, 1727 m, 16 July 2004; IINH 38816 (30 specs), 66°31.42'N, 20°56.69'W, 200 m, 27 July 2004.

##### Additional material.

(17 specs) **Barents Sea**: ZMBN 127338 (1 spec.), Finnmark 71°16.53'N, 27°0.94'E, 278 m, 16 Apr 2011; ZMBN 127336, (1 spec. used for DNA sequencing, SPH 279), Finnmark, 71°20.262'N, 25°13.17'E, 297 m, 23 Apr 2011. ZMBN 127337 (1 spec. used for DNA sequencing, SPH013), Finnmark 71°16.53'N, 27°0.94'E, 278 m, 16 Apr 2011; **Skagerrak**: ZMBN 127335 (4 spec.), 58°35.254'N, 10°19.395'E, 274 m, 14 May 2009; ZMBN 127334 (3 spec.), 58°33.795'N, 10°23.725'E, 254 m, 14 May 2009; ZMBN 103136 (1 spec. used for DNA sequencing, SPH008), 58°35.254'N, 10°19.395'E, 274 m, 14 May 2009; ZMBN 127333 (1 spec. used for DNA sequencing, SPH014.), Skagerrak 58°33.795'N, 10°23.725'E, 254 m, 14 May 2009; ZMBN 125434 (1 spec. used for DNA sequencing, SPH316 photographed alive, Fig. 27A), 58°30.733'N, 10°25.109'E, 275 m, 14 May 2009; ZMBN 127339 (1 spec. used for DNA sequencing, SPH317), 58°30.733'N, 10°25.109'E, 275 m, 14 May 2009; ZMBN 127340 (1 spec. used for DNA sequencing, SPH318), 58°30.733'N, 10°25.109'E, 275 m, 14 May 2009; ZMBN 127341 (1 spec. used for DNA sequencing, SPH319), 58°30.733'N, 10°25.109'E, 275 m, 14 May 2009.

##### Diagnosis.

Body ellipsoid with strongly convex dorsum and flat ventrum, up to 6 mm long. Median antenna and head appendages digitiform, elongated. Median antenna smooth, shorter than other head appendages. Lateral antennae and palps similar, with 4–10 papillae (spurs) on proximal half. Antenniform papillae absent. Tentacular cirri digitiform, with 2–3 elongated papillae on proximal third. Dorsal macrotubercles stalked, without terminal papilla, arranged in 10–12 longitudinal rows in mid-body chaetigers; stalk half as long as tubercle, with 0–1 small papilla on proximal half. Dorsum with up to additional 50–60 spherical-oval papillae with short stalk, in front of each row of macrotubercles, somewhat arranged in 3–4 irregular transverse rows roughly following a zig-zag pattern. Ventrum with ca. 20 papillae per segment in mid-body, arranged in at least four more or less defined transverse rows in a zig-zag pattern. Parapodia with digitiform acicular lobe from chaetiger 3; large ventral cirri, not surpassing the length of acicular lobe; mid-body parapodia with 7–8 papillae. Chaetae blades showing slight gradation in length between chaetigers, slightly shorter in posterior chaetigers; ca. 8–9 times longer than maximum width.

##### Description.

*Measurements and general morphology.* Holotype 5.5 mm long, 0.8&nbsp;mm wide; with 29 segments (Figs 25A, 27A). Body ellipsoid with strongly convex dorsum and flat ventrum. Segmentation not distinct. Pigmentation absent (Fig. 27A).

*Head.* Prostomium with five digitiform elongated appendages, including a pair of palps and lateral antennae, similar in size and shape, and a shorter median antenna (Fig. 25C, D). Lateral antennae and palps with ca. 8–10 papillae (spurs) on proximal half. Tentacular cirri shorter than lateral antennae and palps, with three papillae on proximal third. Many rounded to digitiform small papillae scattered around head appendages (Fig. 25C, D).

*Tubercles.* First chaetiger with 12 dorsal macrotubercles (Fig. 25A); following chaetigers each with one transverse row of 12 (sometimes 11 or 13) dorsal macrotubercles, last chaetiger with ten macrotubercles. Macrotubercles spherical to club-shaped with a stalk near half-length of macrotubercle; first six chaetigers with smooth stalk, from chaetiger 7 backwards stalk provided with small basal papilla (Figs 25B, 26B); all macrotubercles mostly similar in shape and size (Fig. 23E). Additional spherical-oval papillae in different sizes over dorsum, with short stalk, somewhat arranged in 3–4 irregular transverse rows per chaetiger roughly following a zig-zag pattern; ranging from 40 to 60 papillae on each mid-body chaetiger (Fig. 23E). Ventral surface with spherical papillae with short stalk, arranged in four transverse rows in a zig-zag pattern, with ca. 20 papillae per segment in mid-body; numbers decreasing towards posterior end (Figs 23F, 26C).

*Parapodia.* Parapodia sub-conical, increasing in size towards chaetiger 3–4 (Fig. 25E–G), ca. 2–2.5 times longer than wide, some with wrinkled appearance (Figs 25E, 26D–G). Acicular lobe anterior to chaetae, digitiform, longer than parapodial papillae and projecting distally (Figs 25G–J, 26G). Ventral cirri digitiform projecting 1/2 to 2/3 as long as acicular lobe on anterior mid-body segments, almost as long as in posterior segments (Figs 25I, J, 26D–G). First three chaetigers with parapodia provided with 3–5 spherical to clavate papillae: one on antero-dorsal surface, one on antero-lateral surface, one on medio-ventral surface, and two on posterior surface opposite to acicular lobe (Fig. 25E, F); following chaetigers through mid-body with up to three additional papillae: one on posterior surface opposite to acicular lobe, one on antero-lateral/lateral surface and one on ventro-basal position (Fig. 5U); last 3–4 chaetigers lacking some of aforementioned papillae.

*Chaetae.* All parapodia with 8–10 compound chaetae, arranged in a curved transverse row around acicular lobe (Figs 5U, 26G, H). Shaft distal end with thin spinulation (Fig. 26I). Serrated, long blades, 8–9 times longer than maximum width, with a curved tip (Fig. 26H, J), blades slightly shorter in posterior chaetigers.

*Pygidium.* Pygidium terminal, with one mid-ventral digitiform anal cirrus projecting beyond last parapodia, flanked by four spherical papillae (2+2) and one pair of clavate anal cirri at base (Fig. 25K).

*Internal features.* Eyes not discernible in holotype. Pharynx extending over three chaetigers.

*Reproductive features.* Sexual structures or genital pores not observed in holotype. Several oblong eggs visible by transparency ca. 170 µm in length.

##### Variation.

Paratypes measuring 1.1–6.0 mm long, 0.4–0.9 mm wide, with 16–30 chaetigers. Most specimens measuring ca. 2–4 mm in length, 0.4–0.7 mm in width with 20–25 chaetigers. Two dark dorsal eyes behind lateral antennae observed in many paratypes. Some variation occurring in number of papillae and spurs on head appendages: lateral antennae and palps with at least four spurs and tentacular cirri with two short papillae near base. Macrotubercles numbering 7–11 on first chaetiger and usually 10–12 in mid-body. Small papilla at base of macrotubercle stalk not distinguished in all specimens, not related to size or degree of contraction of stalks or body. Short stalk of body papillae (dorsum and ventrum) not always distinguished. Variation in number and distribution of body, ventrum, and parapodial papillae similar to holotype. Pharynx extending over 3–4 chaetigers. Sexual dimorphism not observed in paratypes or additional material examined; several females with oocytes observed.

##### Remarks.

*Sphaerodoridiumceliae* sp. n. is characterized by the unique combination of following features: head appendages with up to ten spurs or basal papillae, 10–12 stalked macrotubercles per mid-body chaetiger, many body papillae among rows of macrotubercles (up to 50 per chaetiger), ventrum of each mid-body chaetiger with at least 20 papillae, and chaetae with blades up to 8–9 times as long as wide.

Sphaerodoridiumcf.minutum, from European waters (see below), also presents a similar range of variation in the number of macrotubercles, many dorsal additional papillae between consecutive rows of macrotubercles and ca. 20 papillae per chaetiger on ventrum. However, *Sphaerodoridiumceliae* sp. n. bears more dorsal papillae per chaetiger showing a more “crowded” appearance (up to 50–60) and parapodial papillae are more numerous (7–8 vs. 3). *Sphaerodoridiumguerritai* is also similar to *Sphaerodoridium*celiae sp. n. in general body appearance and size but dorsal body papillae are less numerous being dorsal side of chaetigers more “smooth”; stalk of macrotubercles are usually provided with at least a small papilla (sometimes up to three) while in *Sphaerodoridiumceliae* sp. n. the presence of the only papilla is more variable across specimens or at least harder to distinguish. The number of parapodial papillae is similar between both species but they differ in their distribution, mostly in the presence in *S.guerritai* of one papilla on the anterior lateral surface that is lacking in *Sphaerodoridiumceliae* sp. n.; the former presents, in turn, one anterior papilla that is present instead on the dorsal surface (cf. Fig. 5U).

The three species recently described from Arctic waters (*S.evgenovi* Gagaev, 2015; *S.kolchaki* Gagaev, 2015; *S.kupetskii* Gagaev, 2015) also present up to 10–14 macrotubercles per chaetiger and dorsal body papillae. However, the original description does not mention explicitly how many papillae are between two consecutive rows of macrotubercles. Furthermore, the drawings of the stalk of the macrotubercles of the three species show a small basal papilla that is not mentioned in the description, and is similar to that present in *S.guerritai* and *Sphaerodorodiumceliae* sp. n. The aforementioned species differ, however, from *Sphaerodoridiumceliae* sp. n. in the number and distribution of parapodial papillae (only 2–3). On the other hand, Gagaev (2015) characterizes *S.evgenovi*, *S.kolchaki* and *S.kupetskii* according to the relative length of anal cirri, macrotubercle stalk and body length but these characters may show variation according to the state of contraction of specimens. Otherwise, they are morphologically close to *S.guerritai* and a comparative review of the four species would be desirable (Capa et al. 2016b).

##### Etymology.

This new species is dedicated to Celia Moreira, in regard of her support and friendship to her brother, JM.

##### Distribution.

Around Iceland and coastal Norwegian waters from the Skagerrak in the south to Finnmark in the north.

##### Habitat.

Soft bottoms, from gravelly sand to silt, at depths of 120–2074 m.

#### Sphaerodoridium claparedii

Taxon classificationAnimaliaPhyllodocidaSphaerodoridae

(Greeff, 1866)

Fig. 5V , 23G, H , 28

Sphaerodorum claparedii Greeff, 1866: 338–350, Taf 6. figs 1–14. Southern 1914: 89–90; Fauvel 1923: 379–380; Amoureux et al. 1978: 88.
Sphaerodoridium claparedii Lützen, 1961: 415; Fauchald 1974: 270. ? Campoy 1982: 464–465; ? Moreira 2012: 26–28, Fig. 5; ? Wehe and Fiege 2002: 138.

##### Type locality.

Dieppe, France, English Channel.

##### Material examined.

(3 specs). **Ireland**: NMINH: 1908.77 (1 spec.), St. Ballynakill xxviii, 0.2 m, 10 Apr 1899; NMINH:1914.313 (1 spec.), St. W236, Blacksod Bay, Co. Mayo, 1.8 m, on 25 Sept 1911; NMINH:1914.313, (1 spec.) Station W181, Blacksod Bay, Co. Mayo, 5.5 m, on 15 Mar 1911.

##### Diagnosis.

Body ellipsoid. Prostomial appendages digitiform, elongated. Median antenna smooth, shorter than lateral antennae. Lateral antennae similar in length to palps, with 3–4 basal papillae (spurs). Tentacular cirri digitiform, smooth, slightly shorter than lateral appendages. Dorsal macrotubercles stalked, smooth, arranged in eight longitudinal rows in mid-body chaetigers; stalk as long as or shorter than tubercle. Dorsum with additional 10–12 rounded papillae between transverse rows of macrotubercles somewhat arranged in a zig-zag pattern. Ventrum with 3–4 transverse rows of papillae per segment. Parapodia with digitiform, large ventral cirri, and acicular lobe; parapodia without papillae, or with a spherical papilla in anterior surface. Approximately six chaetae per parapodium, with short blades (ca. four times as long as wide).

##### Description of Irish material.

**Figure 28.**
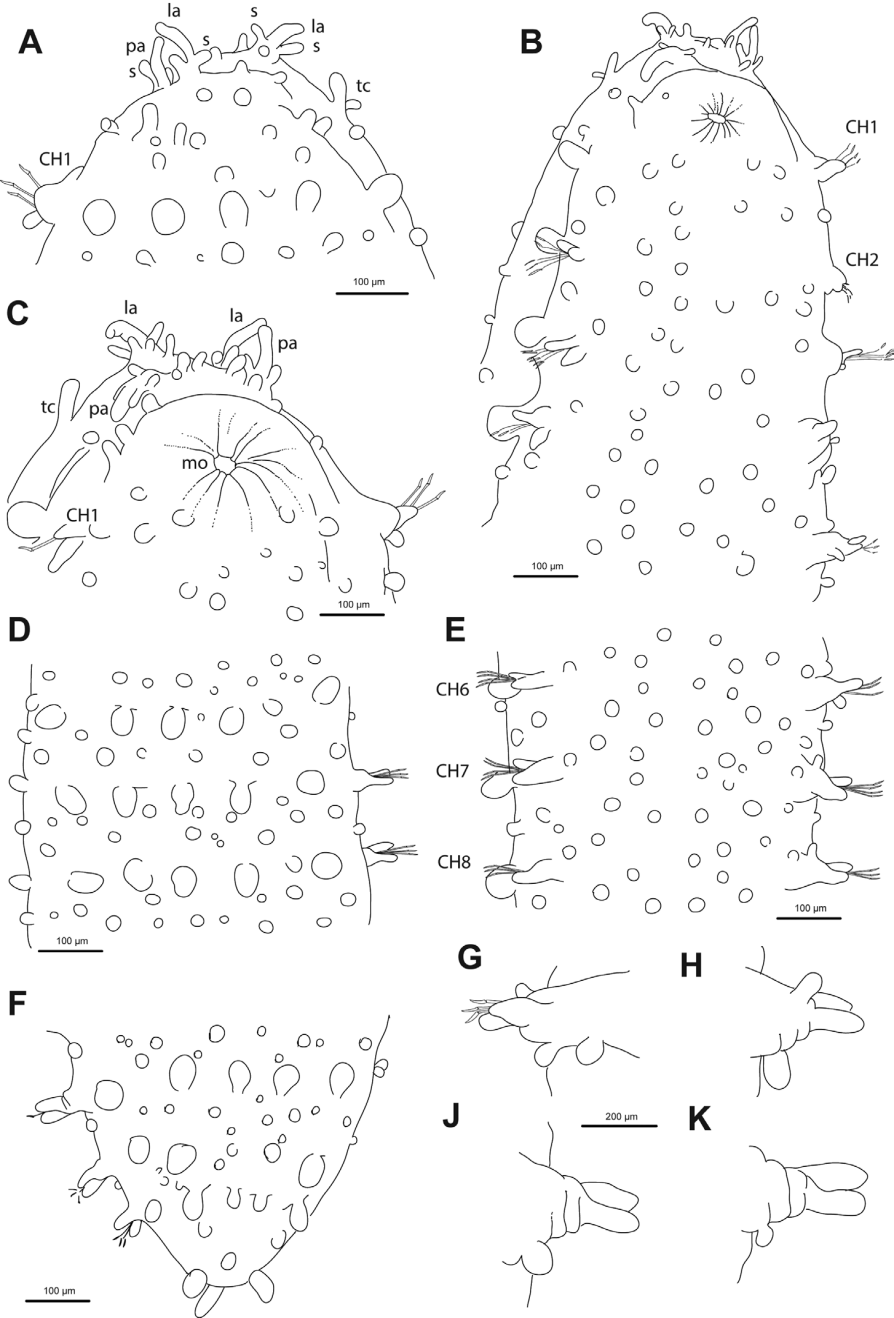
*Sphaerodoridiumclaparedii*, line drawings (MNINH 1908.77.31). **A** Anterior end, dorsal view **B** anterior end, ventral view, detail **C** detail of anterior end, ventral view **D** mid-body chaetigers, dorsal view **E** same, ventral view **F** posterior end, dorsal view **G** chaetiger 5, left side, dorsal view **H**&nbsp;chaetiger 5, right side, ventral view **I** chaetiger 6, left side, dorsal view **K** chaetiger 8, left side, dorsal view

*Measurements and general morphology.* Body ellipsoid, with rounded anterior and posterior ends, with convex dorsal surface and flat ventrum. Segmentation inconspicuous and pigmentation absent (Fig. 28A, B).

*Head.* Prostomium fused to first segment (Fig. 28A, B). Prostomial appendages including palps and lateral antennae similar in shape and size, digitiform and longer than wide, except for median antenna that is shorter and similar to other rounded prostomial papillae. Lateral antennae with 3–4 basal papillae or spurs (Fig. 28C). Tentacular cirri, similar in size and shape to prostomial appendages (Fig. 28B).

*Tubercles.* Dorsal macrotubercles, spherical, smooth, and with a short stalk, arranged in eight longitudinal rows from second chaetiger (six in first chaetiger), and one transverse dorsal row per chaetiger (Figs 23H, 28A, C, D). All macrotubercles similar in size. Approximately 10–12 spherical papillae per segment over dorsum (less in anterior and posterior segments), arranged in three irregular transverse rows (Fig. 28A, D). Ventral papillae hemispherical, arranged in four longitudinal rows (Fig. 28B).

*Parapodia.* Parapodia conical, slightly longer than wide (Fig. 28G–J). Ventral cirri digitiform, similar on shape and size to the acicular lobe (Fig. 28G–J). Parapodial papillae absent or in some parapodia, one spherical papilla in anterior surface, close to the base of parapodium (Figs 5V, 28G–J).

*Chaetae.* Four to six compound chaetae on each parapodium, with short and wide blades (ca. four times as long as wide), unidentate, finely serrated; all similar in size and shape. A single straight acicula per parapodium.

*Pygidium.* Pygidium with paired anal cirri resembling macrotubercles and medio-ventral digitiform anal papilla.

*Internal features.* Two dark eyes visible dorsally inside the head of holotype. Pharynx extending over two chaetigers.

*Reproductive features.* Not described. Sexual structures not observed in Irish material.

##### Variation.

The species was described as bearing six rows of longitudinal and stalked macrotubercles (Fig. 23G). The number of longitudinal rows of macrotubercles counted in the Irish material is eight (Fig. 23H), but two of the lateralmost rows were difficult to assess given the strongly convex dorsum of specimens, that hide them below the animal, in specimens flattened by the microscopy slides (Fig. 28A, C). This could also be the case in the holotype. Some features were not addressed in the original description but found in the Irish specimens (Southern 1914). These are the presence of a short median antenna, and a pygidial median papilla. These attributes seem to be the norm in other sphaerodorids and therefore considered as present in *S.claparedii* comb. n. Irish specimens were smaller than the holotype, all (including mature adults) with less than 18 chaetigers and measuring up to 1.8 mm long. An important note on the variation observed among the studies reported in the literature concerns the number of ventral papillae. While the species was described with four transverse rows of papillae in the ventrum (Greeff 1866), the material collected in Claire Island has small papillae scattered in the ventrum, resembling the dorsal papillae (Southern 1914). Parapodia were described without papillae but in the Irish specimens, a spherical papilla is present in the anterior side of some parapodia.

##### Remarks.

This species has only been reported twice, from Dieppe, France (Greeff 1866, original description), and Claire Island, Ireland (Southern 1914, complementary morphological features). There are reports of this species across European coasts (Atlantic and Mediterranean) and the Red Sea but no description was provided (e.g., Saldanha 1974, Amoureux et al. 1978, Méndez and Cardell 1996). For instance, Mòllica (1995) considers the presence of this species in Italy as doubtful.

*Sphaerodoridiumclaparedii* is distinguished from other congeners by the presence of only eight rows of stalked macrotubercles, a feature that is shared by *S.amoureuxi* comb. n. (if macrotubercles are considered with a short stalk), *S.campanulata* comb. n. and *S.guerritai*. *Sphaerodoridiumguerritai* is clearly distinguished from *S.claparedii* in the presence of stalked macrotubercles with papillae prostomial appendages also with spurs, and 5–6 parapodial papillae. *Sphaerodoridiumcampanulata* comb. n. was described as bearing different sized bell-shaped macrotubercles, not arranged in clear longitudinal or transverse rows (Borowski 1994), unlike the turgid and in line dorsal tubercles of *S.claparedii*.

##### Distribution.

English Channel and western coast UK. ? Atlantic coast of Iberian Peninsula, ? Mediterranean, ? Red Sea (Campoy 1982, Wehe and Fiege 2002).

##### Habitat.

Among algae and shallow sediments (1–5 m). Also collected in planktonic samples (Southern, 1914).

#### Sphaerodoridium gudmunduri

Taxon classificationAnimaliaPhyllodocidaSphaerodoridae

(Moreira & Parapar, 2012) comb. n.

**Figure 29.**
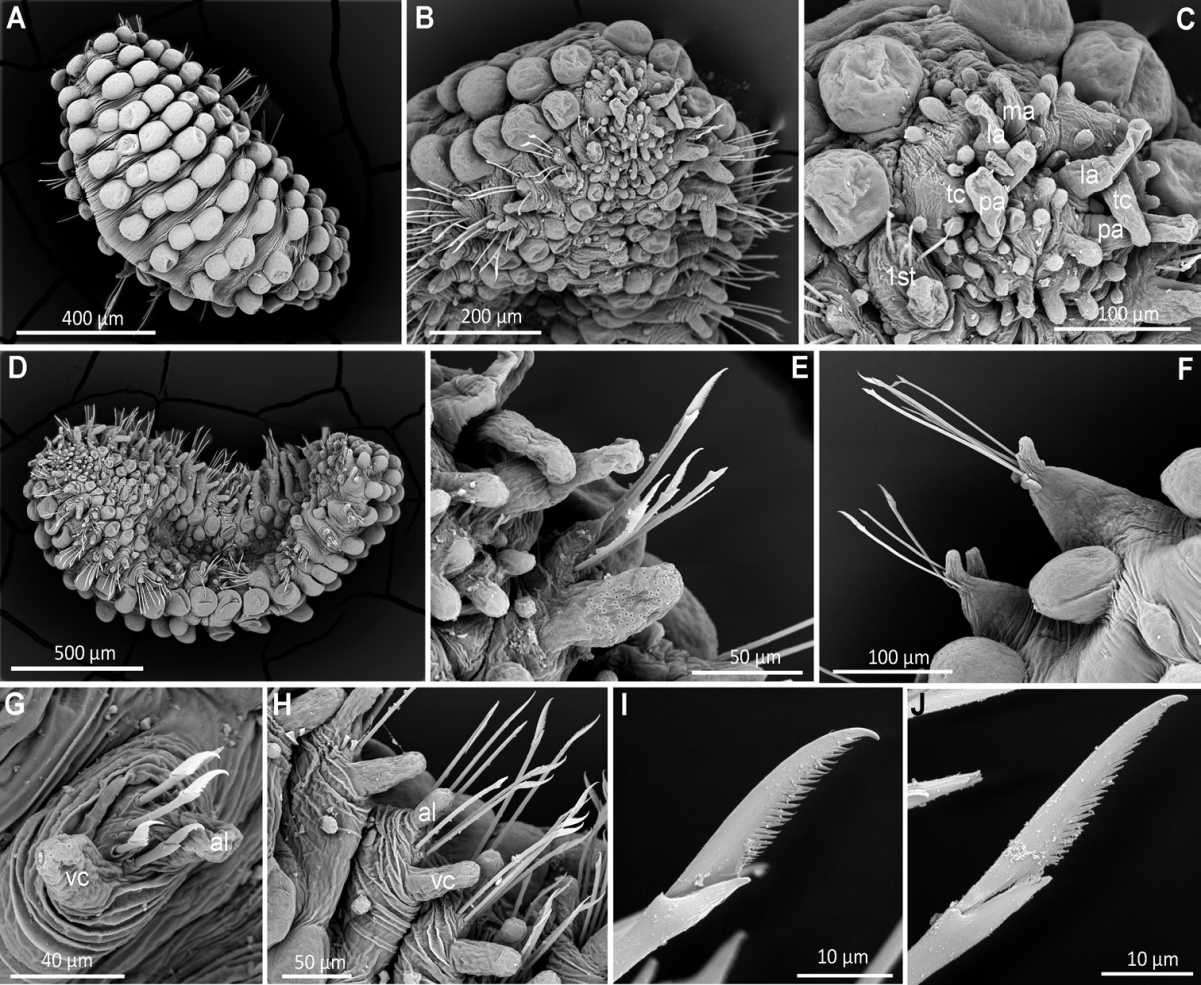
*Sphaerodoridiumgudmunduri* (NTNU-VM 74199), scanning electron micrographs. **A** Complete specimen, dorsal view **B** anterior end, frontal view **C** detail of head, frontal view **D** complete specimen, ventral view **E** parapodium, chaetiger 1, anterior view **F** parapodium, anterior chaetiger, dorsal view **G** parapodium, mid-body chaetiger, side view **H** parapodia, mid-body chaetigers, anterior view **I, J** detail of chaetae, mid-body chaetigers.

Figs 5W , 23I, J , 29

Sphaerodoropsis gudmunduri Moreira & Parapar, 2012: 585–588, figs 1A, 2–3, 6A–C.

##### Type locality.

Northwest Iceland, 66°33.95'N, 20°00.71'W, 97 m.

##### Material examined.

(11 specs) **South Greenland**: ZMBN 127350 (5 spec.), 63°07'N, 52°17'W, 162.5 m, 06 Nov 2002; NTNU-VM 74199 (2 specs on SEM stub), 63°07'N, 52°17'W, 162.5 m, 06 Nov 2002. **Norwegian Sea**: ZMBN 127349 (6 spec.) 64°26.1'N, 11°10.2'W, 400 m, 07 Jun 1983.

##### Diagnosis.

Body short and ellipsoid, less than 2 mm long. Prostomial appendages smooth, lacking spurs. Median antenna as long or slightly shorter than other prostomial appendages. Lateral antennae, palps, and tentacular cirri of similar shape and length. Antenniform papillae absent. 8–12 longitudinal rows of large spherical and sessile macrotubercles in one transverse row per mid-body segment. Dorsum without papillae; ventrum with seven (♂) or 9 (♀) spherical papillae per segment. Parapodia with 1–4 papillae. Acicular lobe from chaetigers 3–4, digitiform. Ventral cirri digitiform, projecting well beyond acicular lobe. Compound chaetae with short blades (less than five times its maximum width), showing little dorso-ventral gradation; unidentate and fine spinulation along its cutting margin.

##### Remarks.

This is the first report of this species from southern Greenland. Specimens show some minor variations to original description. The species was described with 20–25 chaetigers but specimens with less segments have been found (16–19, Fig. 29A), measuring 1.5–2 mm long, 0.5–0.8 mm wide, and with maximum number of macrotubercles in mid-dorsal segments of eight (Fig. 29A) instead of the 10–12 previously described (Moreira and Parapar 2012). This variation could be size related, since the specimens examined for this study are smaller and have less segments than those from original description. Females with small oocytes visible through epidermis. Males observed with a pair of digitiform and distally opened sexual structures between parapodia or segments 7–8 (Fig. 23J), as in original description (Moreira and Parapar 2012). Other diagnostic features are shared by the North East and South Greenland specimens.

Differences between this species and other congeners are the large, sessile, and spherical macrotubercles, arranged in one single transversal row of up to 12 per segment, and absence of any other dorsal papillae (Fig. 29A). Other members of *Sphaerodoridium* with sessile macrotubercles bear also additional papillae between transverse rows. In addition, *S.gudmunduri* comb. n. presents large papillae in ventrum, especially close to the lateral edges, nearby the parapodia (Fig. 29D). All other *Sphaerodoridium* bear smaller ventral papillae.

##### Distribution.

From East Iceland to South Greenland, Norwegian Sea (Moreira and Parapar 2012, present study).

##### Habitat.

Silty sand, at depths of 88–400 m (Moreira and Parapar 2012, this study).

#### Sphaerodoridium guerritai

Taxon classificationAnimaliaPhyllodocidaSphaerodoridae

Moreira & Parapar, 2015

Figs 5X , 23K, L

Sphaerodoridium guerritai Moreira & Parapar, 2015: 93–103, figs 1–6.

##### Type locality.

Northern Iceland, 67°16.86'N, 16°37.77'W, 600 m.

##### Material examined.

(51 specs) **Iceland**, SMF 25299 (8 specs), North-East Iceland, Norwegian Sea, 66°32.63'N, 012°52.48'W, 317,2 m; SMF 23900 (1 spec. used for DNA sequencing SPH 058), North-East Iceland, Norwegian Sea, 66°32.63'N, 12°52.48'W, 317,2 m; SMF 24845 (1 spec. used for DNA sequencing, SPH 059), North-East Iceland, Norwegian Sea, 66°32.63'N, 12°52.48'W , 317,2 m; **Svalbard**, NTNU-VM 73794 (2 specs), Svalbard, Hinlopen, 79°43.1'N, 18°19.9'E, 433 m, 17 Aug 2003; NTNU-VM 73795 (1 spec.), Svalbard, Hinlopen trench, 80°23.8'N, 16°11.9'E, 420 m, 29 Aug 2003; ZMBN 127357 (3 specs), 80°9.144'N, 16°56.124'E, 340 m, 28 Aug 2009; ZMBN 127356 (1 spec. SPH278), 80°9.144'N, 16°56.124'E, 340 m, 28 Aug 2009; ZMBN 127358 (1 spec. used for DNA sequencing, SPH 327), 80°9.144'N, 16°56.124'E, 340 m, 01 Sep 2009; **Norwegian Sea**: ZMBN 127351 (9 specs), 62°29.5'N, 01°43.3'E, 604 m, 21 Jan 1982; ZMBN 127352 (2 specs), 62°35.1'N, 1°47.6'E, 656 m, 23 May 1984; ZMBN 127353 (9 specs), 63°02.9'N, 00°47.8'E, 1293 m, 12 Jan 1985; ZMBN 127354 (1 spec.), 62°50.6'N, 1°25.9'E, 951 m, 15 Aug 1986; ZMBN, 127355 (>10 specs), 63°2.23'N, 4°41.34'E, 760 m, 30 Sep 2013; SMF 25300 (1 spec.), East Iceland, 66°18.060'N, 012°22.380'W, 730,8 m, 22 Sep 2011.

##### Diagnosis.

Body ellipsoid, up to 8 mm long. Median antenna and prostomial appendages digitiform, elongated. Median antenna smooth, near half of length of lateral antennae. Lateral antennae longer than palps, with 6–10 basal papillae (spurs). Antenniform papillae absent. Tentacular cirri digitiform, with two elongated papillae on proximal third. Dorsal macrotubercles stalked, without terminal papilla, arranged in 11–12 longitudinal rows in mid-body chaetigers; stalk as long as or slightly longer than tubercle, with 1–3 small papillae along proximal half. Dorsum with additional 10–16 hemispherical spherical papillae in front of each row of macrotubercles, somewhat arranged in two irregular transverse rows following a zig-zag pattern. Ventrum with 10–18 papillae per chaetiger, arranged in three more or less defined transverse rows. Parapodia with digitiform acicular lobe from chaetiger 3; large ventral cirri, not surpassing the length of acicular lobe; midbody parapodia with 5–6 papillae. Chaetae blades showing gradation in length within and between chaetigers, slightly shorter in mid-body to posterior chaetigers; ca. six times longer than maximum width.

##### Remarks.

The description of this species is complete and no amendments or comments are needed.

##### Distribution.

First record of the species in Svalbard and Norwegian Waters. Already reported around Iceland (Moreira and Parapar 2015).

##### Habitat.

Mostly in muddy sediments (sandy silt and silt), 49–1253 m depth (Moreira and Parapar 2015, present study).

#### Sphaerodoridium cf. minutum

Taxon classificationAnimaliaPhyllodocidaSphaerodoridae

(Webster & Benedict, 1887)

**Figure 30.**
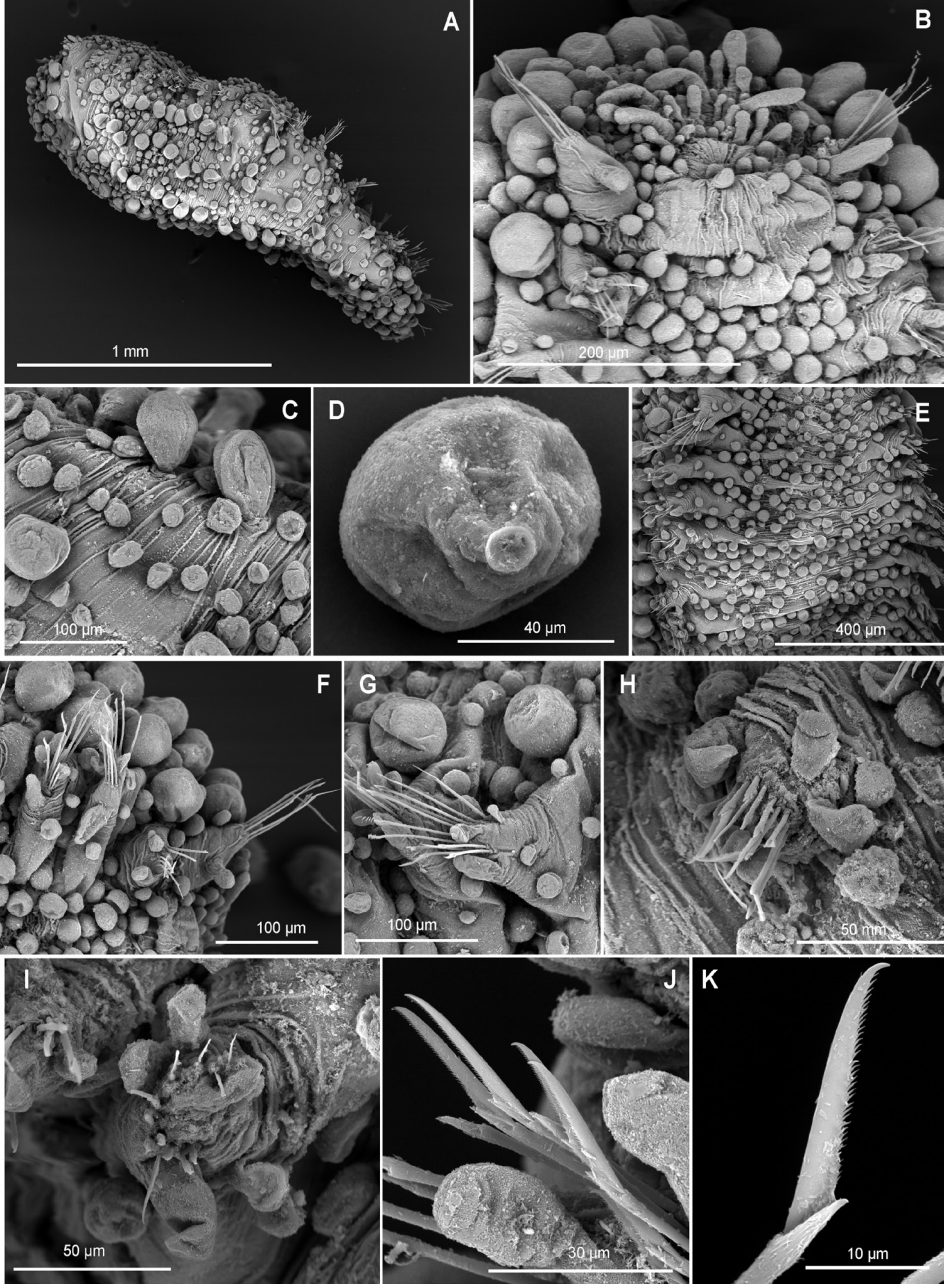
Sphaerodoridiumcf.minutum, from Barents Sea (ZMBN 129500), scanning electron micrographs. **A** Complete specimen, dorsal view **B** anterior chaetigers, ventral view **C** dorsal macrotubercles (slightly stalked) and epithelial papillae, mid-body chaetigers **D** detached macrotubercle, showing short peduncle **E** ventral papillae, mid-body chaetigers **F** parapodia, chaetigers 1–4, side view **G** parapodium, chaetiger 5, anterior view **H** parapodium, chaetiger 3, posterior view **I** parapodium, chaetiger 5 **J** chaetal fascicle, mid-body chaetiger **K** chaetae, detail.

Figs 5Y , 23M, N , 27B, C , 30

Ephesia minuta Webster & Benedict, 1887: 728–729, pl. IV, figs 64–66.
Sphaerodoropsis minuta .– Imajima 1969: 153–154, fig. 2; Hartmann-Schröder 1996: 237; Moreira 2012: 39–41, fig. 13.
Sphaerodorum minutum .– Berkeley and Berkeley 1948: 27–28, fig. 34.
Sphaerodoridium minutum .– Lützen 1961: 415; Capa et al. 2016b: 19–23, fig. 7.

##### Type locality.

Off Maine, United States, North Atlantic Ocean, shelf depths.

##### Material examined.

**Lectotype**: USNM 393, Eastport, Maine, United States, North Atlantic Ocean, coll. Webster, H. E; **Paralectotypes**: USNM 1407984 (11 specs and 4 slides), Eastport, Maine, United States, North Atlantic Ocean, coll. Webster, H. E. Paratypes: USNM 22873 (29 specs, 3 for SEM) Eastport, Maine, United States, North Atlantic Ocean, coll. Webster, H. E.

##### Additional material.

(13 specs) **South Greenland**, ZMBN 127345 (1 spec.), 63°21'N, 52°35'W, 105,5 m, 07 Nov 2002; **Svalbard**, ZMBN 127344 (1 spec. for DNA sequencing SPH277), 79°43.434'N, 11°5.55'E, 216 m, 27 Aug 2009; **Barents Sea**, ZMBN 129500 (1 spec. in SEM stub), Finnmark, 71°20.262'N, 25°13.17'E, 297 m, 23 Apr 2011. **Great Britain**: ZMBN 127346 (1 spec. for DNA sequencing SPH 320), Plymoouth, Mount Sant Michelle, 50°7.148'N, 5°28.419'W, 15 m, 16 Mar 2011; ZMBN 127347 (1 spec. for DNA sequencing SPH 321), The Sound, Plymoouth, 50°21.5'N, 4°8.9'W, 15 m, 16 Mar 2011; ZMBN 127348 (1 spec. for DNA sequencing SPH 322), The Sound, Plymoouth, 50°21.5'N, 4°8.9'W, 15 m, 16 Mar 2011.

##### Diagnosis.

Body short and ellipsoid. Prostomial appendages digitiform, smooth, lacking spurs; median antenna as long as or slightly shorter than other head appendages. Antenniform papillae absent. Ten to twelve longitudinal rows of spherical and stalked macrotubercles in one transverse row per segment, in mid-body segments. Additional spherical papillae arranged in three transverse rows per segment, in dorsum and ventrum. Parapodia with acicular lobe from chaetiger 3, digitiform; ventral cirri digitiform, projecting well beyond acicular lobe; four spherical parapodial papillae. Compound chaetae with medium length blades (6–7 times as long as wide), showing little dorso-ventral gradation.

##### Description of NEA material.

*Measurements and general morphology.* Body with oval contour strongly convex dorsum and flat ventrum. Size range of material examined 20–27 chaetigers; 2–5 mm long; 0.8–0.9 mm wide. Segmentation not distinct. Pigmentation absent in live or fixed material (Figs 27B, C, 30A).

*Head.* Prostomium with five short and digitiform appendages, including a pair of palps and lateral antennae, similar in size and shape, and a shorter median antenna (Fig. 30B). Tentacular cirri shorter than lateral antennae and palps. A few rounded small papillae scattered around head appendages (Fig. 30B).

*Tubercles.* First chaetiger with eight dorsal macrotubercles; following chaetigers each with one transverse row of dorsal macrotubercles increasing to 10–12 tubercles per segment from chaetiger 5 (Fig. 23M). Macrotubercles spherical to club-shaped with a short and smooth stalk (Fig. 30C, D); all macrotubercles similar in shape and size. Additional spherical and sessile papillae in different sizes over dorsum, arranged in 2–3 irregular transverse rows per chaetiger; 20–30 papillae on each mid-body chaetiger (Fig. 30A, C). Ventral surface with spherical papillae in different sizes, arranged in 2–3 transverse rows in a zig-zag pattern, with ca. 20 per segment in mid-body (Fig. 23N); numbers decreasing towards posterior end (Fig. 30E, F).

*Parapodia.* Parapodia sub-conical, increasing in size towards chaetiger 3, ca. 2 times longer than wide (Fig. 30F, G). Acicular lobe anterior to chaetae, digitiform to clavate, longer than parapodial papillae and projecting distally (Fig. 30G, H). Ventral cirri digitiform projecting 1/2 to 2/3 as long as acicular lobe on anterior and mid-body segments, almost as long as in posterior segments (Fig. 30 F–I). Parapodia with three spherical to clavate papillae: one on antero-dorsal surface, one on antero-basal position, and one on the posterior surface (Fig. 30 F–I).

*Chaetae.* All parapodia with 4–7 compound chaetae, arranged in a curved transverse row around acicular lobe (Fig. 30G–I). Serrated, long blades, 4–5 times longer than maximum width, with a curved tip (Fig. 30J, K), similar throughout.

*Pygidium.* Pygidium terminal, with one mid-ventral digitiform anal cirrus projecting beyond parapodia, and one pair of clavate anal cirri, at base on median cirrus.

*Internal features.* Specimens are all opaque after fixation and preservation and internal features not observable.

*Reproductive features.* Sexual structures or eggs not seen in type specimens.

##### Remarks.

*Sphaerodoridiumminutum* (as re-described by Capa et al. 2016b) is characterized by having up to 10–12 macrotubercles in mid-body, a parapodium that bears three (sometimes four) parapodial papillae and compound chaetae with blades 4–5 times as long as maximum width on mid-body chaetigers. Furthermore, the macrotubercles have a short stalk that was overlooked until Capa et al. 2016b reported their morphology. This species was originally described from NW Atlantic coasts (Webster and Benedict 1887) and since then has been reported worldwide (see Capa et al. 2016b for a comparison with related species). However, it is likely that many records from other oceans might refer to other similar, yet undescribed species. Specimens examined from NE Atlantic reported here differ from those of NW Atlantic in having chaetae with slightly longer blades (6–7 times as long as wide) and four parapodial papillae. They might represent a new species but to truly assess this possibility more material from other European localities (both Atlantic and Mediterranean) should be examined. Anyway, NE Atlantic specimens could be distinguished from *S.celiae* sp. n. and *S.guerritai* because the latter bear prostomial appendages with spurs, the stalk of the macrotubercles are longer and parapodia are provided with more papillae (6–7 and 7–8 respectively).

##### Distribution.

Reported as a common species in the North Atlantic and Arctic. However, some records should be reviewed as they could be misidentifications (e.g., Moreira 2012).

##### Habitat.

Shelf or slope depths (Moreira 2012, Capa et al. 2016b).

### Identification key to genera and NEA sphaerodorid species

**Table:** 

1	Body elongate, with somewhat parallel sides (except for blunt anterior and tapering posterior ends). Two longitudinal rows of dorsal macrotubercles (large tubercles) with terminal papilla (one pair per segment)	**2**
–	Body ellipsoid (sometimes elongate). Dorsal tubercles different	**3**
2	Parapodia with only simple chaetae	*** Sphaerodorum ***
–	Parapodia with only compound chaetae (except of, sometimes chaetiger 1)	*** Ephesiella ***
–	Parapodia with both compound and simple chaetae	*** Ephesiopsis *^1^**
3	Dorsum with four longitudinal rows of sessile macrotubercles, in a single transverse row per segment	**4**
–	Dorsum with more than four longitudinal rows of tubercles	**5**
4	All chaetae simple, unidentate, enlarged subdistally. Macrotubercles and dorsal papillae small, most of dorsal surface smooth	*** Commensodorum commensalis ***
–	All chaetae compound. Large macrotubercles, covering most of dorsal surface. Additional papillae often present… *Sphaerephesia*	**12**
5	Dorsal macrotubercles stalked, without terminal papilla, arranged in up to six longitudinal rows, one transverse row per segment…. *Clavodorum*	**6**
–	Dorsal macrotubercles (sometimes not clearly larger than other dorsal papillae) stalked or sessile, arranged in more than six longitudinal rows or in more than one transverse row per segment	**7**
6	Dorsum with additional 10–12 papillae per segment, in two irregular transverse rows, following a zig-zag pattern. One or two parapodial papillae	*** Clavodorum fauchaldi ***
–	Dorsum with additional epithelial papillae other than the six longitudinal rows of macrotubercles absent. Three parapodial papillae	***Clavodorumkristiani* comb. n., nom. n.**
7	Dorsal tubercles small and of similar size (difference in size between them less than twice), in several transverse rows per segment. All chaetae simple unidentate, enlarged subdistally… *Euritmia*	**8**
–	Dorsal tubercles include macrotubercles and papillae (less than half of the size of macrotubercles)	**9**
8	Dorsal tubercles in four irregular transverse rows per segment. Parapodia with a large dorsal papilla	*** Euritmia hamulisetosa ***
–	Dorsal tubercles in three irregular transverse rows per segment. Parapodia without papilla	***Euritmianordica* sp. n.**
9	Tubercles sessile, arranged in two transverse rows per segment *Geminofilum* gen. n	**10**
–	Tubercles sessile or stalked, arranged in one single transverse row per segment *Sphaerodoridium*	**19**
10	Dorsal macrotubercles sessile, hemispherical, arranged in two transverse rows per segment, with five and six macrotubercles each, from segment 2	**11**
–	Dorsal macrotubercles sessile, almost spherical, arranged in two transverse rows per segment, with six and seven macrotubercles each, from segment 3	***Geminofilumhalldori* comb. n.**
11	Dorsum with 4–6 additional papillae per segment in mid-body. Parapodia without papillae	***Geminofilumdistichum* comb. n.**
–	Dorsum with additional five papillae per segment in mid-body. Parapodia with one papilla on anterior surface	***Geminofilumgarciaalvarezi* comb. n.**
12	Dorsal macrotubercles hemispherical, clearly wider than high	***Sphaerephesiamartinae* comb. n.**
–	Dorsal macrotubercles spherical, pear-shaped or with a terminal papilla	**13**
13	?Dorsal papillae star-shaped	***Sphaerephesiastellifer* comb. n., nomen dubium**
–	Dorsal papillae rounded (spherical, hemispherical, ellipsoid)	**14**
14	Parapodia with more than 10 papillae	**15**
–	Parapodia with less than 10 papillae	**16**
15	Parapodia with ca. 12–14 papillae. Compound chaetae, 10–15, with medium length blades (ca. 6–8 times as long as wide)	***Sphaerephesialaureci* comb. n.**
–	Parapodia with 20–40 spherical papillae. Compound chaetae, up to 40, with medium length blades (3–8 times as long as wide)	***Sphaerephesiamultichaeta* sp. n.**
–	Parapodia with ca. 16–19 papillae. Chaetae, 20–25, with long blades (9–13 times as long as wide)	***Sphaerephesiasibuetae* comb. n.**
16	Parapodia with more than five papillae	**17**
–	Parapodia with less than five papillae	**18**
17	Parapodia with 7–8 papillae, larger papilla in dorso-distal position. Approximately 20–25 compound chaetae with long blades (ca. 8–12 times as long as wide)	***Sphaerephesialongipapillata* comb. n.**
–	Eight to ten parapodial papillae. Approximately eight compound chaetae with medium length blades (ca. ten times as long as wide)	***Sphaerephesiaphilippi* comb. n. (inc. *Sphaerephesia* sp. 1)**
18	Parapodia with 3–4 sub-equal papillae. Compound chaetae with long blades (8–20 times as long as wide)	***Sphaerephesiaartabrensis* comb. n.**
–	Parapodia with four papillae. Ventral cirri digitiform reaching tip of acicular lobe. About eight compound chaetae with medium length blades (ca. 7–9 times as long as wide); unidentate	***Sphaerephesiaponsi* sp. n.**
19	Dorsal macrotubercles sessile	**20**
–	Dorsal macrotubercles stalked	**22**
20	Dorsum with additional papillae between transverse rows of macrotubercles	**21**
–	No additional papillae covering dorsum	***Sphaerodoridiumgudmunduri* comb. n.**
21	Lateral antennae with 6–8 spurs	***Sphaerodoridiumamoureuxi* comb. n.**
–	Lateral antennae with 3–4 spurs	***Sphaerodoridiumbalticum* comb. n.**
22	Eight macrotubercles in mid-body segments	***Sphaerodoridiumclaparedii* comb. n.**
–	Ten to 12 macrotubercles in mid-body segments	**23**
23	Lateral antennae and palps with spurs. Dorsal macrotubercles with stalk about half as long as tubercle, with 0–1 small papilla on proximal half. Parapodia with 7–8 papillae. Chaetae with blades about 8–9 times longer than wide	***Sphaerodoridiumceliae* sp. n.**
–	Lateral antennae and palps with spurs. Dorsal macrotubercles with stalk as long as or slightly longer than tubercle, with 1–3 small papillae along proximal half. Parapodia with 5–6 papillae. Chaetae with blades ca. six times longer than wide	*** Sphaerodoridium guerritai ***
–	Prostomial appendages lacking spurs. Dorsal macrotubercles with stalked shorther than tubercle, without basal papillae. Parapodia with four spherical parapodial papillae. Chaetae with blades 6–7 times as long as wide	** Sphaerodoridium cf. minutum **

## Summary and discussion

The North East Atlantic holds a large diversity of species belonging to the family Sphaerodoridae compared with other worldwide regions (26 before this study, 22 of which are regarded as short-bodied forms). This is probably due to historic and economic reasons: European taxonomists have been thoroughly working along the coastline and in deeper waters for more than two centuries. Seven of these species were described after 2000 (Moreira et al. 2004, Aguirrezabalaga and Ceberio 2005, Moreira and Parapar 2007, 2012, 2015). This is related to the number of contemporary expeditions in this geographic area, focusing on continental shelf and slope environments, where sphaerodorids seem to be most abundant (Fauchald 1974, Borowski 1994, Capa et al. 2014). However, there was still material waiting to be studied in museum collections and other institutions that has nurtured the present project.

The present integrative taxonomic study, including morphological examination and DNA analyses of specimens, has allowed us to assess the presence of 25 species of short-bodied sphaerodorids including four new species: *Euritmianordica* Capa & Bakken, sp. n., *Sphaerephesiamultichaeta* Capa, Moreira & Parapar, sp. n., *Sphaerephesiaponsi* Capa, Parapar & Moreira, sp. n., and *Sphaerodoridiumceliae* Moreira, Capa & Parapar, sp. n. In addition, the synonymisation of *S.chardyi* is herein proposed and the presence of *S.parva* in the area (Desbruyères 1980) has not been confirmed.

Some of the most revealing outcomes of the present study are the results obtained after analyses of the DNA sequences of selected specimens. After some recent papers, the family Sphaerodoridae was regarded to contain six genera (Capa et al. 2016b), although there was indication that some of them (at least *Sphaerodoridium* and *Sphaerodoropsis*) were not monophyletic (Capa et al. 2016a). The analyses carried out herein are far from being a comprehensive revision of the family, but they allow confirming paraphyly of most of the groups equivalent to genus level in current classification (Read and Fauchald 2018) and involve nomenclatural changes to accommodate these results.

The phylogenetic hypothesis presented herein, is congruent with that presented by Capa et al. (2016), with *Sphaerodoropsis* and *Sphaerodoridium* (*sensu* Read & Fauchald, 2018) being paraphyletic, but since it increases the number of taxa considered, offers more details regarding the relationships and content of some groups. The results also impulses the erection of a new genus to accommodate the species previously considered as *Sphaerodoropsis* with two transverse rows of dorsal macrotubercles per segment herein named as *Geminofilum* gen. nov. Nevertheless, further analyses considering type species of traditional genera are required in order to confirm the diagnosis and delimitation of these groups. Incorporation of members of *Euritmia* and *Sphaerodoropsis* Group 4 (according to Borowski 1994) will also allow to test their position in the sphaerodorid radiation and if they are closely related, as suggested by Capa et al. (2016b).

The newly proposed classification suggests that the main feature characterising genera is the number of longitudinal and transverse rows of dorsal macrotubercles, and not so much the shape of these macrotubercles (as per Fauchald 1974).

## Supplementary Material

XML Treatment for Clavodorum

XML Treatment for Clavodorum fauchaldi

XML Treatment for Clavodorum kristiani

XML Treatment for Commensodorum

XML Treatment for Commensodorum commensalis

XML Treatment for Euritmia

XML Treatment for Euritmia hamulisetosa

XML Treatment for Euritmia nordica

XML Treatment for Geminofilum

XML Treatment for Geminofilum distichum

XML Treatment for Geminofilum halldori

XML Treatment for Geminofilum garciaalvarezi

XML Treatment for Sphaerephesia

XML Treatment for Sphaerephesia artabrensis

XML Treatment for Sphaerephesia laureci

XML Treatment for Sphaerephesia longipapillata

XML Treatment for Sphaerephesia martinae

XML Treatment for Sphaerephesia multichaeta

XML Treatment for Sphaerephesia philippi

XML Treatment for Sphaerephesia ponsi

XML Treatment for Sphaerephesia sibuetae

XML Treatment for Sphaerephesia stellifer

XML Treatment for Sphaerephesia

XML Treatment for Sphaerodoridium

XML Treatment for Sphaerodoridium amoureuxi

XML Treatment for Sphaerodoridium balticum

XML Treatment for Sphaerodoridium celiae

XML Treatment for Sphaerodoridium claparedii

XML Treatment for Sphaerodoridium gudmunduri

XML Treatment for Sphaerodoridium guerritai

XML Treatment for Sphaerodoridium cf. minutum

## Footnotes

Not reported in NEA waters
